# Four-Electron Reduction of O_2_ Using Distibines in the Presence of *ortho*-Quinones

**DOI:** 10.1021/jacs.3c02223

**Published:** 2023-06-12

**Authors:** Benyu Zhou, François P. Gabbaï

**Affiliations:** Texas A&M University, Department of Chemistry, College Station, Texas 77843, United States

## Abstract

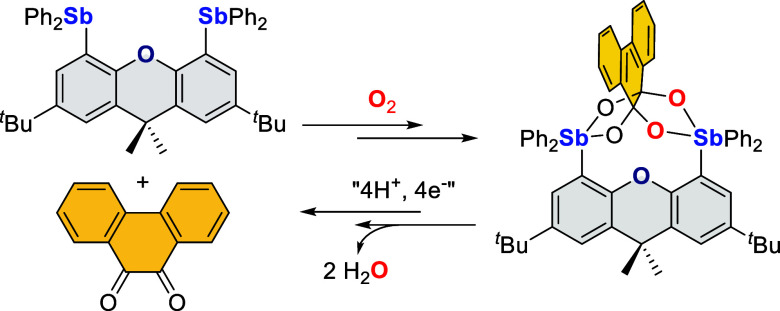

This study, which aims to identify atypical platforms for the reduction of dioxygen, describes the reaction of O_2_ with two distibines, namely, 4,5-bis(diphenylstibino)-2,7-di-*tert*-butyl-9,9-dimethylxanthene and 4,5-bis(diphenylstibino)-2,7-di-*tert*-butyl-9,9-dimethyldihydroacridine, in the presence of an *ortho*-quinone such as phenanthraquinone. The reaction proceeds by oxidation of the two antimony atoms to the + V state in concert with reductive cleavage of the O_2_ molecule. As confirmed by ^18^O labeling experiments, the two resulting oxo units combine with the *ortho*-quinone to form an α,α,β,β-tetraolate ligand that bridges the two antimony(V) centers. This process, which has been studied both experimentally and computationally, involves the formation of asymmetric, mixed-valent derivatives featuring a stibine as well as a catecholatostiborane formed by oxidative addition of the quinone to only one of the antimony centers. Under aerobic conditions, the catecholatostiborane moiety reacts with O_2_ to form a semiquinone/peroxoantimony intermediate, as supported by NMR spectroscopy in the case of the dimethyldihydroacridine derivative. These intermediates swiftly evolve into the symmetrical bis(antimony(V)) α,α,β,β-tetraolate complexes via low barrier processes. Finally, the controlled protonolysis and reduction of the bis(antimony(V)) α,α,β,β-tetraolate complex based on the 9,9-dimethylxanthene platform have been investigated and shown to regenerate the starting distibine and the *ortho*-quinone. More importantly, these last reactions also produce two equivalents of water as the product of O_2_ reduction.

## Introduction

O_2_ reduction is an essential biological reaction that enables life-sustaining processes, including energy conversion, metabolism, and the synthesis of important biomolecules.^1^ All of these processes rest on the involvement of enzymes that activate the otherwise inert triplet ground state of molecular O_2_ while also channeling its reaction along specific pathways. Most of these enzymes feature transition metals at their active sites,^2^ although metal-free systems such as flavoenzymes^3^ also exist. The study of these naturally occurring systems has inspired the development of numerous synthetic derivatives that mimic the oxygenase or oxidase reactivity found in nature.^4^ While most synthetic systems contain transition metals, recent efforts have explored the use of p-block derivatives as alternative, potentially metal-free, platforms for oxygen fixation reactions.^5−10^

**Scheme 1:**
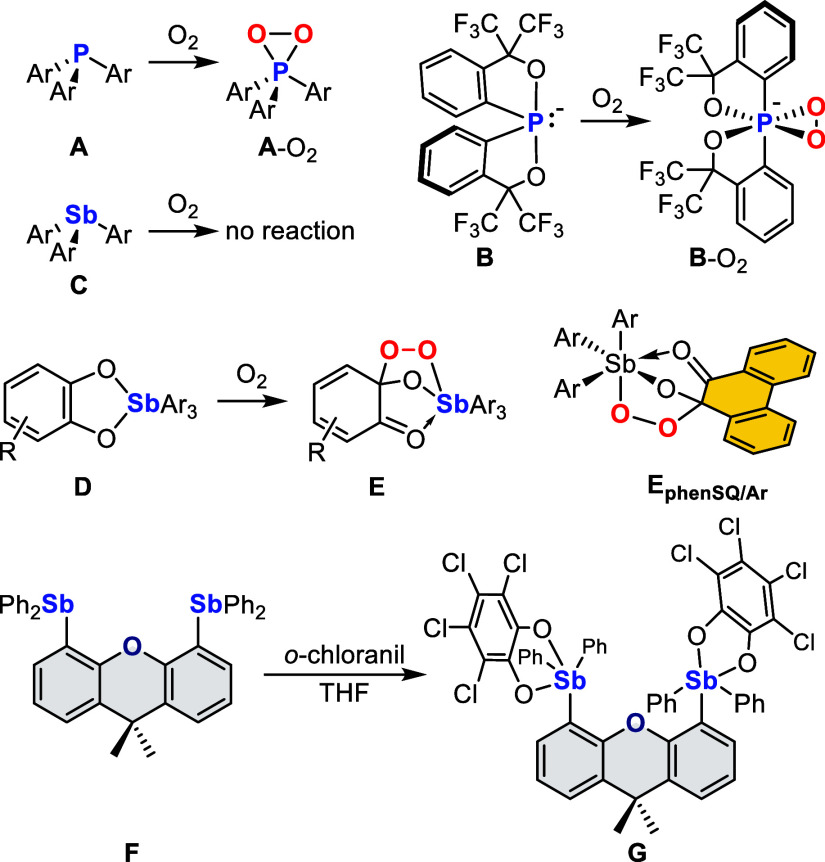
Selected Reactions of Group 15 Compounds with O_2_

Among the various p-block derivatives investigated in this chemistry, those containing group 15 elements (Scheme 1) are particularly interesting because of their rich redox chemistry and their reactions with O_2_.^11^ Phosphines are well known to undergo rapid oxidation to afford the corresponding phosphine oxides, which has been proposed to involve dioxaphosphiranes of type **A** as intermediates.^12^ The closest structurally characterized analogue of such species is an anionic complex (**B**–O_2_) obtained by oxidation of the corresponding phosphoranide **B**.^13^ In agreement with electrochemical data,^14^ the oxidation of heavier triarylpnictines is less favorable. In fact, triarylarsines and triarylstibines (**C**) are air stable and necessitate more potent reagents to access the pentavalent state.^15^ Examples of such reagents include *ortho*-quinones that are particularly well-adapted to the conversion of stibines into the corresponding catecholatostiboranes (**D**).^16^ Interestingly, when carried out in air, this chemistry may also involve O_2_. Indeed, as demonstrated by Cherkasov et al., stiboranes resulting from the reaction of Ph_3_Sb with certain *ortho*-quinones may fixate O_2_ to form semiquinone (SQ) peroxide adducts of type **E**, in which the O_2_^2–^ unit bridges the antimony center and one of the carbon atoms of the ligand.^17^ One of the simplest examples of such a compound is **E**_**phenSQ/Ph**_, obtained by the reaction of Ph_3_Sb and 9,10-phenanthraquinone (phenQ) in air. This chemistry, which has also been observed with iminoquinones,^17b,17c,18^ illustrates the potential of group 15 compounds as platforms for the two-electron reduction of O_2_. Given that the four-electron reduction of O_2_ is often regarded as more complicated due to the possible formation of stable intermediates and the occurrence of radical side reactions,^19^ we have now decided to test whether distibines,^20^ aided by an *ortho*-quinone, could spontaneously reduce and split O_2_. Inspired by the prevalence of bifunctional 9,9-dimethylxanthene-based constructs in artificial oxygen reduction catalysts,^21^ we have decided to focus on distibines related to **F** (Scheme 1), which, as per our prior investigations, readily reacts with the electron-poor *ortho*-quinone *o*-chloranil to afford the corresponding distiborane **G**.^20b^ In this article, we show that such distibines, when in the presence of an electron-rich *ortho*-quinone, can reduce O_2_, broadening the type of redox reactions that group 15 systems can mediate.^22^

## Results and Discussion

### Synthesis of the Distibines and Reactivity toward *ortho*-Quinones

**Figure 1:**
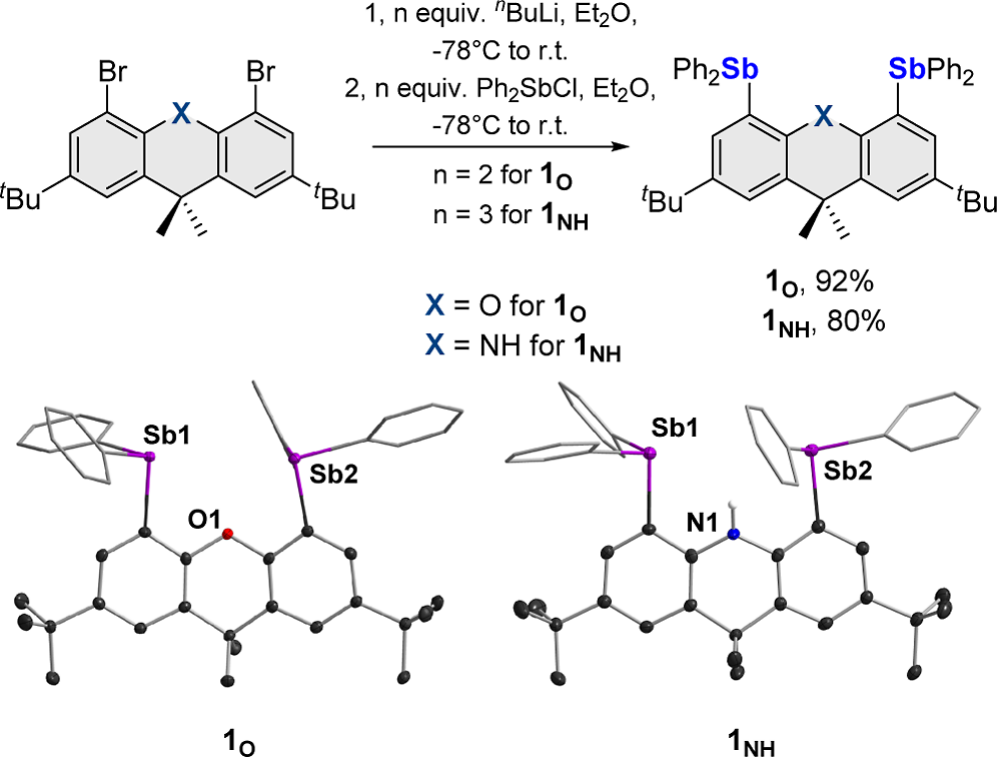
Synthesis and solid-state structures of **1**_**O**_ or **1**_**NH**_.

To work on a platform with better NMR spectroscopic handles, we targeted the 2,7-di-*tert*-butyl analogue of **F**. Toward this end, 4,5-dibromo-2,7-di-*tert*-butyl-9,9-dimethylxanthene was dilithiated^23^ and allowed to react with two equivalents of Ph_2_SbCl (Figure 1). This reaction afforded the distibine **1**_**O**_ as a colorless air-stable solid whose properties resemble those of **F**. The ^1^H NMR spectrum features easily identifiable resonances corresponding to the methyl and *tert*-butyl groups. Solutions of **1**_**O**_ in CDCl_3_ remain intact for several hours, indicating that this compound resists oxidation. A single-crystal X-ray diffraction analysis shows that the two antimony atoms are separated by a distance of 4.1717(5) Å (Figure 1), which is very close to the distance of 4.1517(4) Å measured in **F**.^20b^ Altogether the structure of **1**_**O**_ is similar to that displayed by phosphorus, arsenic, and bismuth-containing analogues.^24^

**Figure 2:**
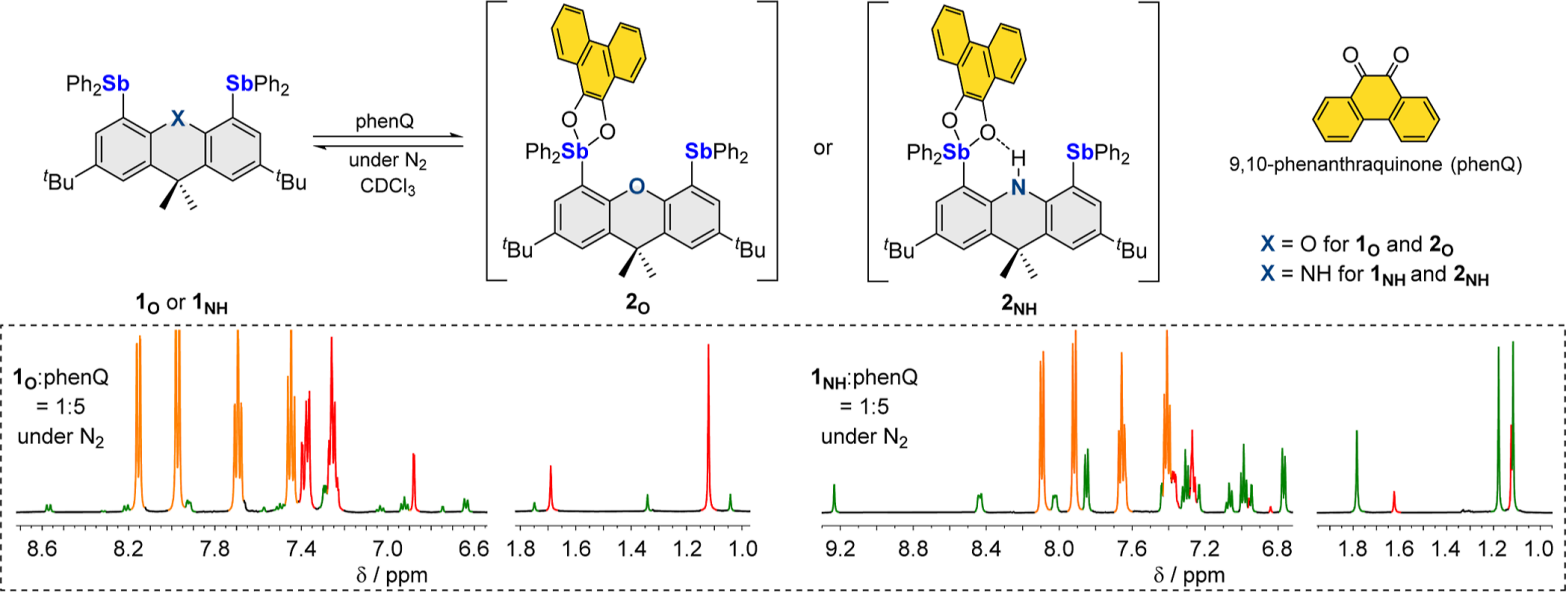
Top: reactions of distibines **1**_**O**_ and **1**_**NH**_ with phenQ. Bottom: portions of the ^1^H NMR spectra (red trace: **1**_**O**_ or **1**_**NH**_; orange trace: phenQ; and green trace: **2**_**O**_ or **2**_**NH**_) of 1:5 mixtures of **1**_**O**_ (left) or **1**_**NH**_ (right) and phenQ in CDCl_3_ under N_2_.

With compound **1**_**O**_ at our disposal, we turned our attention to its reaction with phenQ, which, like several *ortho*-quinones,^16a−16q^ is known to oxidatively add to Ph_3_Sb.^17b^ We were also interested in employing this quinone, given that its reaction under aerobic conditions with Ph_3_Sb yields the semiquinone peroxide adduct of type **E**_**phenSQ/Ph**_.^17b^ We first studied this reaction in the absence of oxygen. Interestingly, the ^1^H NMR spectrum recorded after mixing **1**_**O**_ and phenQ in a 1:1 molar ratio displays a set of resonances readily assignable to **1**_**O**_ and phenQ, along with new signals of extremely weak intensities (see the Supporting Information). Increasing the concentration of phenQ leads to an increase in the intensity of these new signals, suggesting the formation of a new species. Analysis of the *tert*-butyl resonance is particularly convenient to monitor this process. Indeed, the *tert*-butyl groups that appear as a single resonance in **1**_**O**_ give rise to two new, equally intense signals as the concentration of phenQ is increased. The appearance of these two signals suggests that phenQ adds oxidatively to only one of the antimony centers. Interestingly, formation of this new product remained incomplete, even in the presence of five equivalent of phenQ (Figure 2). Carrying out this experiment at varying concentrations of phenQ affords a formation constant of only 1.2 ± 0.13 M^–1^ for this new product, referred to as **2**_**O**_. The existence of an equilibrium between **1**_**O**_ and phenQ is in contrast with the facile formation of distiboranes such as **G** when electron-poor *ortho*-quinones are used.^20b,25^ Aiming to influence the formation of this adduct via secondary interactions, we decided to prepare **1**_**NH**_, an analogue of **1**_**O**_ featuring a central NH group which, unlike the oxygen atom of **1**_**O**_, could act as a hydrogen bond donor functionality. Emulating an approach used for the synthesis of a related diphosphine,^26^ 4,5-dibromo-2,7-di-*tert*-butyl-9,9-dimethyl-9,10-dihydroacridine was triply lithiated. The resulting organolithium reagent was treated with three equivalents of Ph_2_SbCl, which, after aqueous workup, afforded the corresponding distibine **1**_**NH**_ (Figure 2). Like **1**_**O**_, **1**_**NH**_ is an air-stable solid whose structure can easily be verified using ^1^H NMR spectroscopy. In addition to resonances corresponding to the methyl and *tert*-butyl groups, its spectrum also features a signal at 6.83 ppm corresponding to the NH group. In the crystal, the two antimony atoms of **1**_**NH**_ are separated by 4.5634(3) Å (Figure 1). This value exceeds that measured in **1**_**O**_ (4.1717(5) Å), which, we propose, originates from the larger covalent size of nitrogen when compared to oxygen.

Next, we turned our attention to the reaction of this new distibine with phenQ. Interestingly, while 1:1 mixtures of **1**_**O**_ and phenQ only display the color of the individual components, mixing **1**_**NH**_ with phenQ in the same molar ratio affords a dark green solution, indicating the occurrence of a more complete reaction (Figure 2). As shown in Figure 2, the ^1^H NMR resonances of this new species (**2**_**NH**_), which become prominent at high phenQ concentrations, indicate oxidative addition of the *ortho*-quinone to only one of the two antimony centers as in the case of **1**_**O**_. The formation constant of 19.1 ± 5.0 M^–1^ for **2**_**NH**_ is significantly larger than that of **2**_**O**_ (1.2 ± 0.13 M^–1^), which can be correlated to the existence of a hydrogen bond between the NH group of **2**_**NH**_ and an oxygen atom of the newly formed 9,10-phenanthrenediolate ligand. This proposal is consistent with the N*H*^1^H NMR resonance of **2**_**NH**_, which appears at 9.20 ppm, downfield from that of **1**_**NH**_ at 6.83 ppm. The lesser electronegativity of N versus O and the larger Sb–Sb separation in the dihydroacridine derivative may be the other contributing factors. Efforts to isolate **2**_**O**_ and **2**_**NH**_ proved unsuccessful, possibly due to their low formation constants and the necessary presence of excess phenQ.

### Reaction of the Distibines with *ortho*-Quinones under Aerobic Conditions

**Figure 3:**
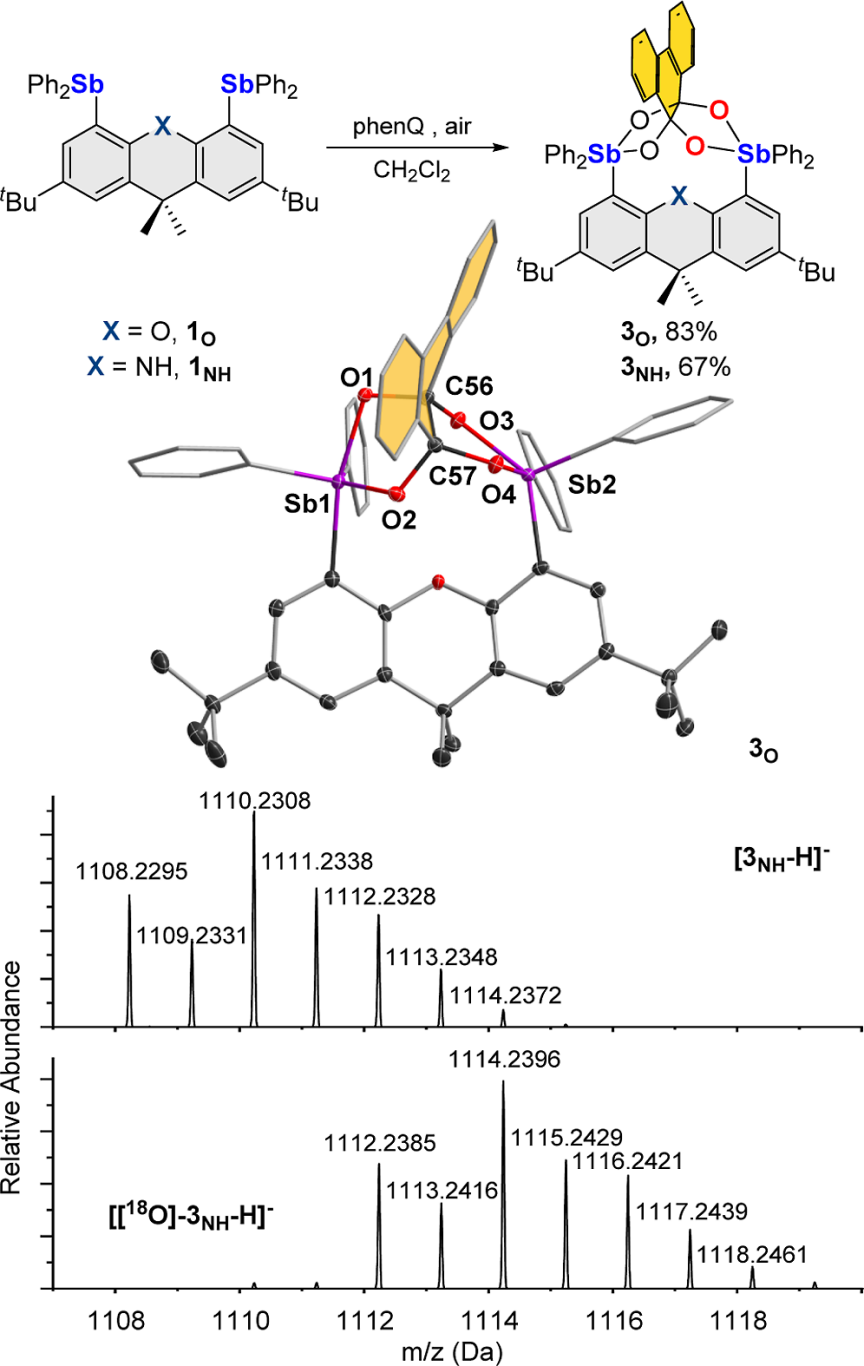
Reaction of **1**_**O**_ and **1**_**NH**_ with phenQ in air, leading to the formation of **3**_**O**_ and **3**_**NH**_. The crystal structure of **3**_**O**_ is also displayed along with the ESI mass spectrum of unlabeled and ^18^O-labeled **3**_**NH**_**.**

With this reactivity baseline established, we decided to carry out the reaction of **1**_**O**_ and **1**_**NH**_ with phenQ under oxygen. Toward this end, we simply combined the distibines with one equivalent of phenQ in CH_2_Cl_2_ under aerobic conditions (Figure 3). Over the course of 2 h, the intense color from the quinone gradually faded to afford a yellow solution. After a simple workup, compounds **3**_**O**_ and **3**_**NH**_ could be isolated as air-stable solids in 83 and 67% yields, respectively, indicating that they are the primary products. The ^1^H NMR spectra of these new species indicate the formation of symmetrical species, easily identifiable by a single *tert*-butyl resonance at 1.15 ppm for **3**_**O**_ and 1.13 ppm for **3**_**NH**_, when recorded in CDCl_3_. These spectra also indicate the presence of a single phenQ unit per distibine. In the case of **3**_**NH**_, the NH resonance appears at 11.72 ppm, significantly downfield from that of **1**_**NH**_ or **2**_**NH**_, suggesting its involvement in the hydrogen bonding interaction. Intrigued by these features, we decided to subject these compounds to ESI–mass spectrometry. While the mass spectrum of **3**_**O**_ was inconclusive and did not display the molecular ion peak, that of **3**_**NH**_, in the negative mode, showed an intense peak corresponding to [**2**_**NH**_ + O_2_ – H^+^]^−^. The detection of this peak suggests that **3**_**NH**_ is the dioxygen addition compound of its corresponding precursor **2**_**NH**_. To confirm that the two additional oxygen atoms originate from dioxygen, we repeated the synthesis of **3**_**NH**_ under an atmosphere of ^18^O_2_. Analyses of the isotopically labeled compound showed a peak at *m*/*z* 1112.239 Da, which is shifted by 4 Da when compared to that of **3**_**NH**_.

**Figure 4:**
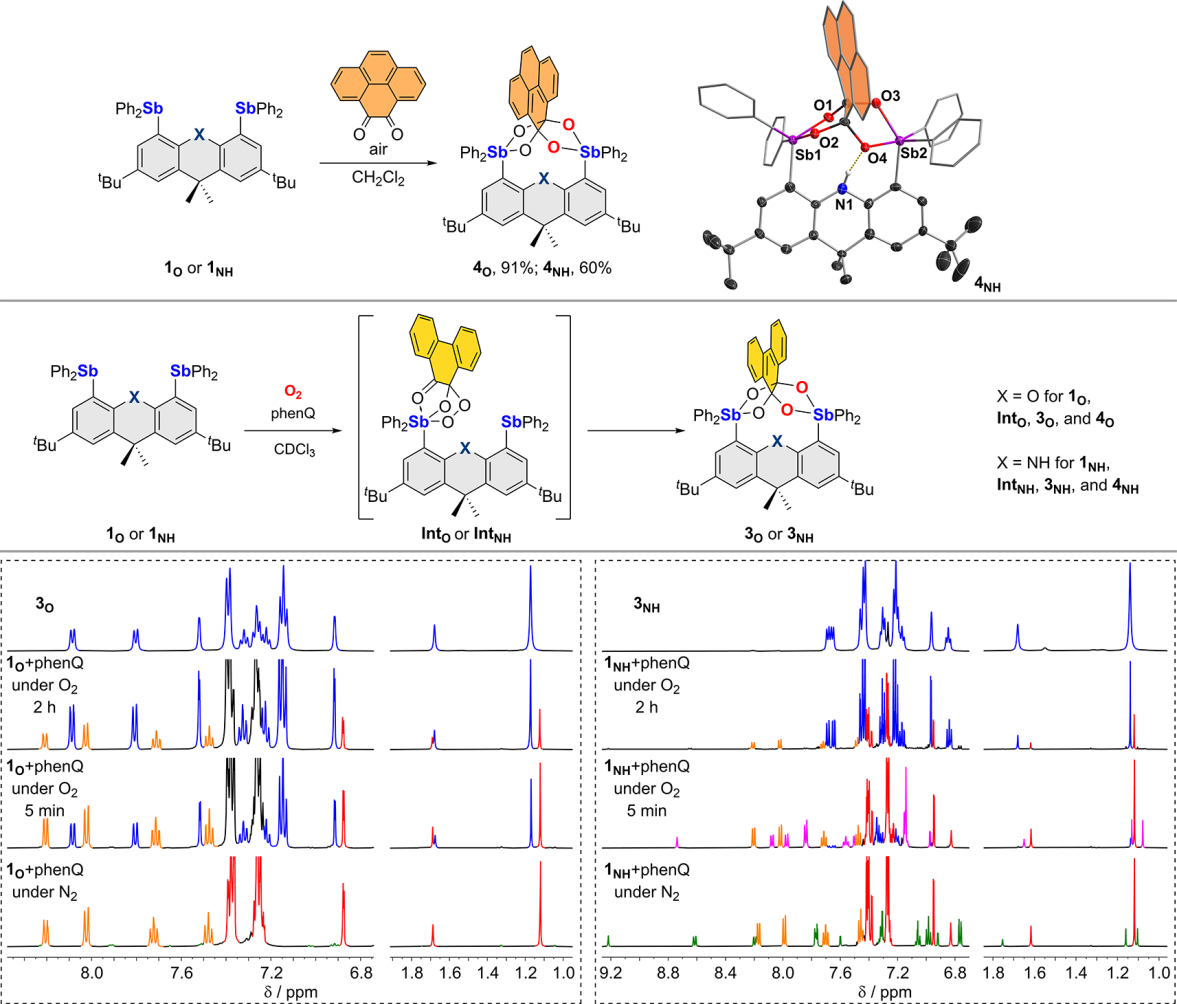
Top: synthesis of **4**_**O**_ and **4**_**NH**_ by the reaction of **1**_**O**_ and **1**_**NH**_, respectively, with pyrene-4,5-dione in air. One of the independent molecules in the crystal structure of **4**_**NH**_ is also shown. Middle: reactions of **1_O_** and **1_NH_** with phenQ under O_2_ monitored by ^1^H NMR. Bottom: portions of the ^1^H NMR spectra (red trace: **1_O_** or **1_NH_**; orange trace: phenQ; green trace: **2_O_** or **2_NH_**; blue trace: **3_O_** or **3_NH_**; magenta trace: **Int_NH_**) of 1:1 mixtures of phenQ and **1_O_** (left) or **1_NH_** (right) in CDCl_3_ under a N_2_ atmosphere, 5 min and 2 h after exposure to O_2_. The spectra of pure **3_O_** and **3_NH_** in CDCl_3_ are also included for reference.

Because mass spectrometry data were not available for **3**_**O**_, we attempted the isolation of this compound in a single crystalline form. Single crystals of **3**_**O**_, which could be easily obtained, were subjected to X-ray diffraction analysis, revealing an unusual phenanthrene-9,9,10,10-tetraolate ligand bridging the two antimony centers, as shown in Figure 3. Formation of this new unit confirms the incorporation of two additional oxygen atoms, thus corroborating the mass spectrometry results obtained for **3_NH_**. There are no anomalies in the resulting C–O and Sb–O distances which all fall in the expected range for single bonds. To the best of our knowledge, such a ligand has been observed in the single case of a tetranuclear molybdenum complex obtained by the reaction of phenQ with a high valent [Mo_2_O_7_]^2–^ salt.^27^ In this case, however, the two newly formed C–O bonds originate from the terminal oxo ligands rather than O_2_. It is interesting to note that, in **3**_**O**_, the redox state of the phenQ unit is not changed as the tetraolate ligand present can be regarded as a doubly hydrated and four-time deprotonated version of phenQ. However, the two antimony atoms of **3**_**O**_ are pentavalent versus trivalent in **1**_**O**_. This change indicates a two-electron oxidation of each antimony atom upon formation of **3**_**O**_. Single crystals of **3**_**NH**_ could not be obtained. Yet, based on the similarity of the spectroscopic attributes, we speculate that it also features a bridging phenanthrene-9,9,10,10-tetraolate ligand. This assumption is borne out by the isolation and structural characterization of a close analogue of **3**_**NH**_, namely, **4**_**NH**_ obtained by the aerobic combination of **1**_**NH**_ with pyrene-4,5-dione instead of phenQ. As shown in Figure 4, **4**_**NH**_ possesses a pyrene-4,4,5,5-tetraolate ligand coordinated to both antimony atoms, leading to a central Sb_2_O_4_C_2_ motif closely related to that in **3**_**O**_. The structure of **4**_**NH**_ also indicates the presence of an NH···O hydrogen bond [N–O distance = 2.645(10) Å and 2.776(9) Å] in both of the independent molecules found in the asymmetric unit of **4**_**NH**_. These interactions are responsible for the downfield shift of the N*H* resonance that appears at 11.81 ppm in the case of **4**_**NH**_, close to the value of 11.72 ppm measured for **3**_**NH**_. As expected, **4**_**O**_ was also readily obtained by combining **1**_**O**_ with pyrene-4,5-dione in air, as confirmed by NMR spectroscopy and X-ray analysis (see the Supporting Information).

**Figure 5:**
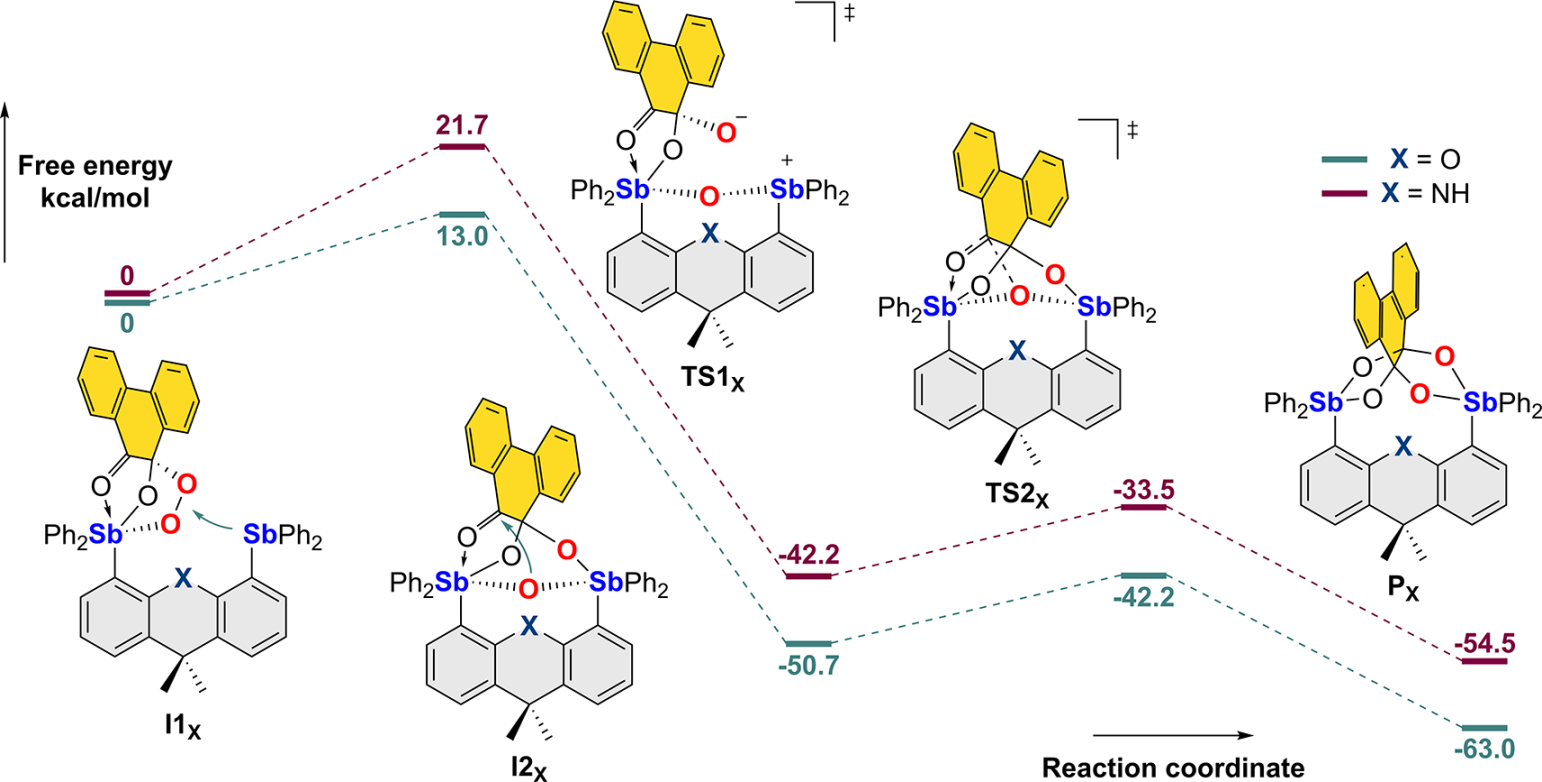
Computed pathway for the isomerization of the putative phenSQ peroxide intermediate into the corresponding phenanthrene-9,9,10,10-tetraolate. Gas-phase optimization and frequency computations of all structures were performed with M06-2X functional and mixed basis sets: def2-svp for C, H, N, O, and aug-cc-pVTZ-PP for Sb. Single-point energy calculations were carried out on the gas-phase-optimized structures using the RI-PWPB95-D3(BJ)/def2-tzvpp method with the SMD solvation model using CH_2_Cl_2_ as the solvent.

**Chart 1:**
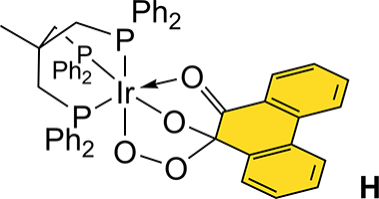
Structure of Complex H

To identify possible intermediates in the reactions leading to **3**_**O**_ or **3**_**NH**_, we decided to monitor their formation using *in situ*^1^H NMR spectroscopy (Figure 4). Toward this end, a 1:1 mixture of phenQ and **1**_**O**_ or **1**_**NH**_ in CDCl_3_ was loaded in a sealed J. Young NMR tube under N_2_ and subsequently exposed to O_2_ (25 psi) to initiate the reaction. Interestingly, upon contact with O_2_, the previously mentioned intermediates **2**_**O**_ or **2**_**NH**_ disappeared immediately, leading to the appearance of the final products **3**_**O**_ or **3**_**NH**_ (see Figure 4, blue trace). When **1**_**O**_ is employed as the starting distibine, no intermediates aside from **2**_**O**_ could be detected. By contrast, in the reaction involving **1**_**NH**_, a new species, referred to as **Int**_**NH**_, was clearly observed in the early stages of the reaction (see Figure 4, magenta trace). This new species is characterized by two ^1^H NMR *tert*-butyl resonances, indicating a different environment for the two antimony atoms. The resonance of the nitrogen-bound proton of this new species is observed at 8.74 ppm, suggesting the continued involvement of the NH functionality in a hydrogen bond motif. We also note that only three resonances are observed for the phenQ (or phenSQ, *vide infra*), suggesting that one of its signals interferes with other resonances. Given these spectroscopic features and the documented reaction of Ph_3_Sb and phenQ under aerobic conditions, we speculate that **Int**_**NH**_ is an analogue of **E**_**phenSQ/Ph**_ in which only one of the two antimony atoms is involved in the formation of the phenanthrasemiquinone (phenSQ) peroxide species. The formation of such compounds has been discussed before this work and is proposed to proceed by the concomitant electrophilic activation of the O_2_ molecule at the antimony center, followed by transfer of an electron from the catecholate unit to the O_2_ fragment.^17b,18a^ The resulting triplet superoxide derivative undergoes heavy-atom-facilitated intersystem spin crossing, followed by cyclization into the phenSQ peroxide species. A similar sequence of steps was recently discussed in the case of a redox-active tetrapyrrole aluminate complex which undergoes a similar oxygen activation reaction.^7f^ In addition to being observed in compounds of type **E**_**phenSQ/Ar**_, such a phenSQ unit has also been characterized in transition-metal complexes, including the iridium complex **H** (Chart 1).^28^ A relevant aspect of this earlier report concerns the detection of four ^1^H NMR resonances for the phenSQ unit, suggesting that the distal oxygen atom of the peroxo unit rapidly exchanges between the adjacent C(O) positions. The same arguments can be used to explain that **Int**_**NH**_ does not display the eight resonances expected for a static, unsymmetrical phenSQ unit. Finally, we propose that **Int**_**O**_ is also formed as an intermediate leading to **3**_**O**_. However, and possibly due to the absence of a stabilizing hydrogen bond donor NH functionality, this compound (**Int**_**O**_) is not observed and rapidly evolves into the product **3**_**O**_.

### Computational Analysis of the Reaction in the Case of a Simplified Model

To test the relevance of a phenSQ peroxide species as an intermediate involved in the oxygen reduction reaction, we decided to compute a possible reaction profile using 4,5-bis(diphenylstibino)-9,9-dimethylxanthene (**F**), a model derivative that lacks the *tert*-butyl groups at the 2 and 7 positions of the dimethylxanthene backbone (Figure 5). Using this platform, we first computed the structure of the phenSQ peroxide derivative, referred to as **I1**_**O**_, and found that the oxidized antimony atom connected to the bridging peroxide unit and the SQ adopts a structure similar to that determined experimentally in the case of the known **E**_**phenSQ/Ph**_ derivative.^17b^ It is also worth pointing out that the trivalent antimony unit of **I1**_**O**_ is oriented inward toward the peroxide moiety. Based on this unique disposition, we speculated that the trivalent antimony atom of **I1**_**O**_ could easily oxidatively insert into the O–O bond to afford intermediate **I2**_**O**_. The known reactivity of triarylstibines with peroxides, including cyclic peroxides, suggested that such a simple insertion reaction was a reasonable step to consider.^29^ Geometry optimization of the insertion product afforded **I2**_**O**_, an intermediate that is 50.7 kcal/mol more stable than **I1**_**O**_. This structure possesses two antimony(V) centers connected to one another by a single oxo ligand. The formation of such an Sb–O–Sb motif on a dimethyl-xanthene platform is reminiscent of the Sb–F–Sb motif formed in the fluoride complex of **G**,^20b^ adding credence to the existence of **I2**_**O**_ as an actual intermediate. The transition state (**TS1**_**O**_) connecting **I1**_**O**_ and **I2**_**O**_ lies only 13.0 kcal/mol over **I1**_**O**_. With an asymmetrical insertion of the free stibine into the O–O bond of the endoperoxide, **TS1**_**O**_ also appears asynchronous, suggesting that **I2**_**O**_ is formed via a polar oxidative addition mechanism. We next turned our attention to the conversion of **I2**_**O**_ into the final product **P**_**O**_. Inspection of the structure of **I2**_**O**_ suggested that this intermediate may evolve into the product by simple nucleophilic attack of the carbonyl functionality of the phenSQ ligand by the oxygen atom bridging the two antimony atoms. A transition state (**TS2**_**O**_) supporting this possibility was quickly identified and found to lie only 8.5 kcal/mol above **I2**_**O**_. Inspection of this transition state supports our proposal that the bridging oxygen atom indeed approaches the carbonyl carbon atom of the SQ ligand. The low-energy barriers identified by this computational survey are consistent with the fact that intermediate **Int**_**O**_ is not observed during the *in situ* oxygen fixation reaction by the dimethylxanthene systems. We studied the same reaction profile starting from **I1**_**NH**_, the dimethyldihydroacridine analogue of **I1**_**O**_. These calculations indicate that the activation energy of the first step of the reaction increases to 21.7 kcal/mol (Figure 5). The higher barrier measured for the conversion of the phenSQ peroxide species (**I1**_**NH**_) into intermediate **I2**_**NH**_ is consistent with our ability to experimentally detect **Int**_**NH**_ by *in situ* NMR measurements (Figure 4).

### Reactivity of the Phenanthrene-9,9,10,10-Tetraolate Complexes

**Figure 6:**
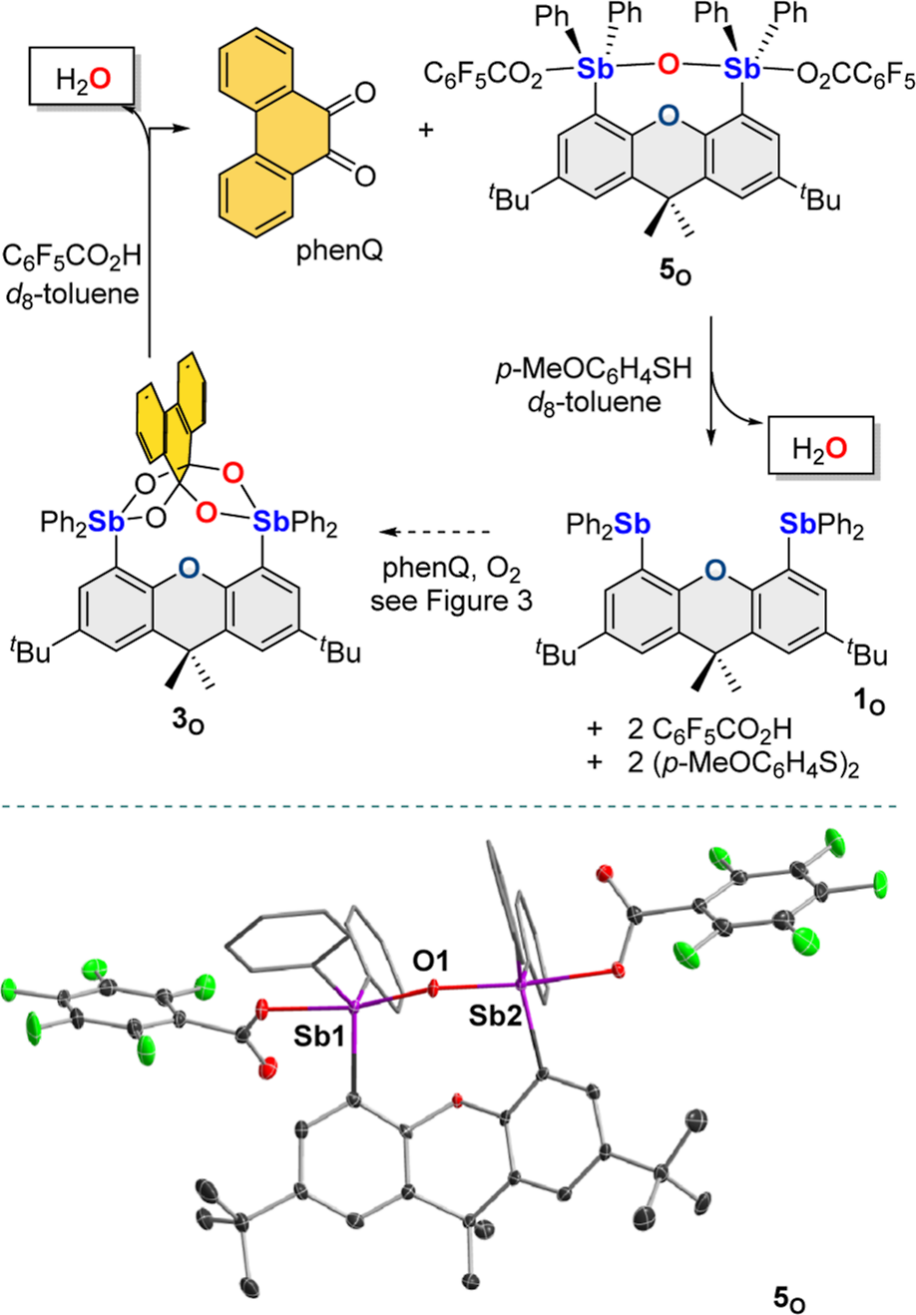
Acidolysis and reduction of **3**_**O**_. The structure of the oxo-bridged species **5**_**O**_, formed in the reaction, is also shown.

To test whether this chemistry presents opportunities for turnover and reduction of O_2_ into water, we decided to investigate the releases of water from the bis(antimony) platforms. Toward this end, **3**_**O**_ and **3**_**NH**_ were treated with C_6_F_5_CO_2_H in *d*_8_-toluene, leading to a color change from light yellow to orange (Figure 6). The ^1^H NMR spectrum collected in the case of **3**_**NH**_ could not be readily interpreted and suggested the formation of a mixture of products. A different outcome was observed in the case of **3**_**O**_. Indeed, the ^1^H NMR spectrum indicated the appearance of a new symmetrical antimony species (**5**_**O**_), accompanied by the release of phenQ and the formation of one equivalent of water observed at 0.71 ppm. Compound **5**_**O**_, identified as the oxygen-bridge species shown in Figure 6, can be independently synthesized starting from **1**_**O**_, ^*t*^BuOOH, and two equivalents of C_6_F_5_CO_2_H in Et_2_O/H_2_O. Complex **5**_**O**_ has been characterized by NMR spectroscopy and ESI–mass spectrometry. Its structure has also been confirmed by single-crystal X-ray diffraction analysis, which shows a bent Sb–O–Sb motif (Sb–O–Sb = 156.29(11)°) in which the oxo ligand is connected to the two antimony atoms via bonds of 1.9634(19) and 1.9682(19) Å. The oxo-bridge motif present in **5**_**O**_ is reminiscent of that in [O(SbPh_3_OTf)_2_],^30^ which displays Sb–O bonds of 1.980(8) and 1.937(10) Å and a more acute Sb–O–Sb angle of 136.5(5)°. The difference observed between the Sb–O–Sb angle of **5**_**O**_ and [O(SbPh_3_OTf)_2_] manifests the influence of the structural constraints imposed by the dimethylxanthene backbone in **5**_**O**_. The structure of **5**_**O**_ indicates that one oxygen atom remains trapped between the two antimony atoms. Fortunately, **5**_**O**_ can be reduced using four equivalents of *p*-methoxy benzenethiol, releasing the second water equivalent and two molecules of C_6_F_5_CO_2_H (Figure 6). The formation of water was again verified by ^1^H NMR spectroscopy which shows a broad signal at 5.2 ppm integrating as four hydrogen atoms, in line with the formation of water and two equivalents of acids. These last two reactions allow us to complete a synthetic cycle which includes the release of two water molecules as the product of oxygen reduction while also leading to the regeneration of the starting distibine and phenQ. Efforts to carry out the reduction of O_2_ catalytically are currently hindered by the reaction of the thiol reducing agent with the quinone.

## Concluding Remarks

The results presented herein introduce a new strategy for the reduction of dioxygen based on a main group element platform. In this approach, we intercept a SQ peroxide adduct of type **E**_**phenSQ/Ar**_ using an adjacent stibine, which we propose originally inserts in the peroxide moiety of the SQ intermediate, thus completing the activation of the original O_2_ molecule. The four-electron O_2_ activation supported by this new platform occurs in two individual reduction steps, each involving the transfer of two electrons. Putting these results in a broader context, we note that SQ peroxide species are pivotal intermediates in the degradation of aromatic compounds promoted by the non-heme iron dioxygenases of certain bacteria.^31^ In this case, these intermediates are formed, en route to C–C bond cleavage reactions that produce ring-opened oxygenated, muconic semialdehyde, or acid derivatives.^32^ In this regard, the reactions described in this contribution provide a new pathway by which the SQ peroxide species can further react without undergoing C–C bond cleavage. Instead, the reaction ultimately produces an α,α,β,β-tetraolate ligand with an intact hydrocarbon core, stabilized by two antimony(V) centers. The net redox reaction leading to this complex involves the two-electron oxidation of each antinomy atom of the platform and the four-electron reduction of O_2_. Finally, we show that the α,α,β,β-tetraolate distiborane derivative can be triggered to release two equivalents of water as the O_2_ reduction product by acidolysis and reduction. These last steps also regenerate the original bis-antimony(III) derivative, presenting opportunities for implementing this chemistry catalytically.

## Supporting Information Available

Experimental and computational details (PDF)
