# Supplementary material for: Four-Electron Reduction of O2 Using Distibines in the Presence of ortho-Quinones
Source: J Am Chem Soc. 2023 Jun 12;145(25):13758–67. doi: 10.1021/jacs.3c02223 (PMC10863049; doi:10.1021/jacs.3c02223)
Supplement: Supplementary file 1 — ja3c02223_si_001.pdf [file ja3c02223_si_001.pdf]

Four-Electron Reduction of O_2 Using Distibines in the Presence of *ortho*-Quinones

Supporting Information

Benyu Zhou and François P. Gabbaï*

Texas A&M University, Department of Chemistry, College Station, Texas 77843, United States

Contents

1 Techniques and methods S2

1.1 NMR spectroscopy S2

1.2 Single crystal X-ray diffraction measurement S2

1.3 Quantum chemical computations S2

2 Experimental Procedures S3

2.1 Syntheses S3

2.2 NMR spectra S10

3 Detection of **2**$_{\mathbf{O}}$ and **2**$_{\mathbf{NH}}$ via *in situ* ^{1}H NMR and determination of their formation constants. S18

4 Formation of **3**$_{\mathbf{O}}$ and **3**$_{\mathbf{NH}}$ under O_2 atmosphere detected via *in situ* ^{1}H NMR S22

5 ^{18}O isotope labeling study using $^{18}O_2$ as the oxygen source S25

6 Reactivities of compounds **3**$_{\mathbf{O}}$ and **5**$_{\mathbf{O}}$. S28

7 Coordinates and energies of all the computed local minima and the transition states. S32

7.1 9,10-phenanthraquinone S32

7.2 O_2 .... S32

7.3 4,5-bis(diphenylstibino)-9,9-dimethylxanthene, **XanSb**$_{\mathbf{2}}$ S33

7.4 4,5-bis(diphenylstibino)-9,9-dimethyl-9,10-dihydroacridine, **AcrSb**$_{\mathbf{2}}$ S34

7.5 Intermediate **I1**$_{\mathbf{O}}$. S35

7.6 Transition state **TS1**$_{\mathbf{O}}$. S36

7.7 Intermediate **I2**$_{\mathbf{O}}$. S37

7.8 Transition state **TS2**$_{\mathbf{O}}$. S38

7.9 Product **P**$_{\mathbf{O}}$. S39

7.10 Intermediate **I1**$_{\mathbf{NH}}$. S40

7.11 Transition state **TS1**$_{\mathbf{NH}}$. S41

7.12 Intermediate **I2**$_{\mathbf{NH}}$ S42

7.13 Transition state **TS2**$_{\mathbf{NH}}$ S43

7.14 Product **P**$_{\mathbf{NH}}$ S44

References S48

1 Techniques and methods

All the air and moisture-sensitive experiments were carried out under a dry nitrogen atmosphere using either the glove box or standard Schlenk techniques. 4,5-Dibromo-2,7-*tert*-butyl-9,9-dimethylxanthene,[1] 4,5-dibromo-2,7-di-*tert*-butyl-9,9-dimethyl-9,10-dihydroacridine,[2] and pyrene-4,5-dione[3] were synthesized following literature procedures. All other chemicals were obtained from commercial suppliers and used without further purification. Solvents were dried by refluxing over Na/K (Et_2O, THF), or CaH_2 (CH_2Cl_2, $CDCl_3$). All other solvents were ACS reagent grade and used as received. Elemental analyses were performed by Atlantic Microlab (Norcross, GA). Mass spectrometry was performed by in-house mass spectrometry facilities.

1.1 NMR spectroscopy

NMR spectra were recorded at room temperature using a Varian Inova 500 FT NMR spectrometer, a Bruker Avance 500 NMR spectrometer, or a Bruker Ascend 400 NMR spectrometer. Chemical shifts are given in ppm. ^{1}H and ^{13}C signals were referenced to residual solvent signals.[4] ^{19}F NMR signals were referenced to $CFCl_3$.

The following abbreviations are used in the compilation of NMR data provided for each compound:

s, singlet; d, doublet; t, triplet; q, quartet; m, multiplet; dd, doublet of doublets; td, triplet of doublets; tt, triplet of triplets; dm, doublet of multiplets.

$^{m}J_{X\text{-}Y}$, coupling constant between nucleus X and Y separated by m bonds.

1.2 Single crystal X-ray diffraction measurement

The crystallographic measurements were performed at 110 K using a Bruker D8 Quest (Mo source) or a Bruker APEX 22 diffractometer equipped with Photon III detectors. Semi-empirical absorption corrections were applied using the Bruker SADABS software package.[5] The structures were solved by direct methods with SHELXT[6] to locate all non-hydrogen atoms. Subsequent refinement using a difference map on F^2 with the SHEXL package[7] allowed for the location of the remaining non-hydrogen atoms which were refined anisotropically. H atoms were added in calculated positions using a riding model. CCDC 2245102-2245107 contain the supplementary crystallographic data for this paper. These data can be obtained free of charge via www.ccdc.cam.ac.uk/data_request/cif, or by emailing data_request@ccdc.cam.ac.uk.

1.3 Quantum chemical computations

All calculations were conducted with the Gaussian 16, revision C.01[8] or ORCA 5.0.2 program.[9] Gas-phase optimizations and frequency calculations were performed using Gaussian 16 with the M06-2X functional[10] and mixed basis sets: def2-svp for C, H, N, O and aug-cc-pVTZ-PP for Sb. Frequency calculations were carried out to verify the nature of the stationary points (no imaginary frequency for the local minima and only one imaginary frequency for the transition states). The connectivity between each transition state and its two neighboring local minima was confirmed by intrinsic reaction coordinate (IRC) calculations. Single point energies were recalculated using ORCA with RI-PWPB95-D3(BJ) functional[11] and def2-tzvpp basis set on the gas-phase optimized structures with SMD solvation model[12] using CH_2Cl_2 as the solvent.

2 Experimental Procedures

2.1 Syntheses

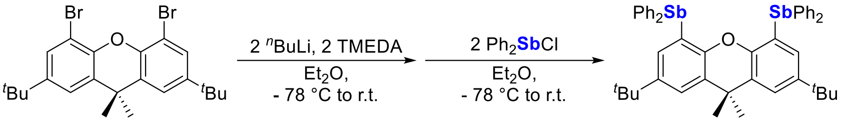

1$_{O}$

4,5-bis(diphenylstibino)-2,7-di-*tert*-butyl-9,9-dimethylxanthene (1$_{O}$): A ^{n}BuLi solution (2.5 M in Hexanes, 3.1 mL, 7.8 mmol) was added dropwise to a suspension of 4,5-dibromo-2,7-di-*tert*-butyl-9,9-dimethylxanthene (1.59 g, 3.31 mmol) and *N*,*N*,*N'*,*N'*-tetramethylethylenediamine (TMEDA, 1.0 mL, 0.78 g, 6.7 mmol) in anhydrous Et_2O (40 mL) cooled to -78 °C. The resulting grey suspension was stirred at -78°C for 1 h, slowly warmed to room temperature, and kept at room temperature for 1 h, affording a light brown solution. This solution was cooled down to -78 °C and treated with Ph_2SbCl (2.42 g, 7.75 mmol) suspended in anhydrous Et_2O (20 mL). The reaction mixture was allowed to slowly warm up to room temperature and kept at this temperature overnight. The resulting mixture was quenched with a sat. aqueous NH_4Cl solution (10 mL). The resulting biphasic mixture was concentrated under reduced pressure, treated with water (40 mL), and extracted with CH_2Cl_2 (60 mL × 3). The organic fractions were combined, dried over anhydrous $MgSO_4$, filtered, and brought to dryness under reduced pressure to afford a light brown solid as the crude product. The crude product was washed with MeOH (15 mL) under sonication, affording **1**$_{O}$ as a white powder (2.65 g, 92%). Single crystals suitable for X-ray diffraction were grown by slow vapor diffusion of Et_2O into a CH_2Cl_2 solution of **1**$_{O}$ in a freezer (-20 °C).

^{1}H NMR ($CDCl_3$, 500.1 MHz): δ 7.39-7.35 (m, 10H, Xan-*H* & Ph-*H*), 7.30-7.21 (partially overlapping with the $CHCl_3$ solvent peak, m, 12H, Ph-*H*), 6.87 (d, $^4J_{H-H}$ = 2.2 Hz, 2H, Xan-*H*), 1.68 (s, 6H, C(CH_3)$_2$), 1.12 (s, 18H, C(CH_3)$_3$).

^{13}C{^{1}H} NMR ($CDCl_3$, 125.8 MHz): δ 151.5 (s), 146.3 (s), 139.2 (s), 136.7 (s), 132.1 (s), 128.7 (s), 128.3 (s), 126.0 (s), 123.7 (s), 35.2 (s, C(*C*H_3)$_2$), 34.7 (s, *C*(CH_3)$_3$), 32.8 (s, *C*(CH_3)$_2$), 31.5 (s, C(*C*H_3)$_3$).

Elemental Analysis: Calculated for $C_{47}H_{48}OSb_2$: C 64.71, H 5.55; Found: C 64.26, H 5.60.

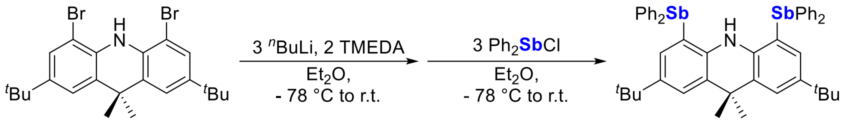

1$_{NH}$

4,5-bis(diphenylstibino)-2,7-di-*tert*-butyl-9,9-dimethyl-9,10-dihydroacrydine (1$_{NH}$): A ^{n}BuLi solution (2.5 M in Hexanes, 5.0 mL, 12.5 mmol) was added dropwise to a suspension of 4,5-dibromo-2,7-di-*tert*-butyl-9,9-dimethyl-9,10-dihydroacridine (1.80 g, 3.76 mmol) and *N*,*N*,*N'*,*N'*-tetramethylethylenediamine (TMEDA, 1.1 mL, 0.85 g, 7.3 mmol) in anhydrous Et_2O (60 mL) at -78 °C. The resulting yellow solution was stirred at -78°C for 1 h, slowly warmed to room temperature, and kept at room temperature overnight. The solution was cooled down to -78 °C and treated with Ph_2SbCl (3.89 g, 12.5 mmol) suspended in anhydrous Et_2O (40 mL). After being allowed to slowly reach room temperature, the reaction mixture was stirred for an additional 36 h. The reaction was then quenched with ^{i}PrOH (~5 mL) and brought to dryness under reduced pressure. The residue was extracted with CH_2Cl_2 (50 mL × 3) and filtered through a pad of silica gel, affording a light green filtrate. Evaporation of the filtrate afforded a light green solid as the crude product. The crude product was washed with a mixture of CH_2Cl_2/Hexanes (0.5 mL/ 20 mL), yielding **1$_{NH}$** as a light green solid (2.63g, 80%). Single crystals suitable for X-ray diffraction were grown by slow evaporation of a CH_2Cl_2/Et_2O solution of **1$_{NH}$**.

^{1}H NMR ($CDCl_3$, 500.1 MHz): δ 7.43-7.39 (m, 8H, Ph-*H*), 7.38 (d, $^{4}J_{H\text{-}H}$ = 2.2 Hz, 2H, Acridine-*H*). 7.30-7.24 (partially overlapping with the $CHCl_3$ solvent peak, m, 12H, Ph-*H*), 6.95 (d, $^{4}J_{H\text{-}H}$ = 2.2 Hz, 2H, Acridine-*H*), 6.83 (s, 1H, N*H*), 1.62 (s, 6H, C(C*H*$_3$)$_2$), 1.12 (s, 18H, C(C*H*$_3$)$_3$).

^{13}C{^{1}H} NMR ($CDCl_3$, 125.8 MHz): δ 143.7 (s), 141.0 (s), 137.1 (s), 136.6 (s), 132.4 (s), 129.0 (s), 128.9 (s),128.8 (s), 122.9 (s), 122.1(s), 37.6 (s, C(*C*H$_3$)$_2$), 34.6 (s, *C*(CH$_3$)$_3$), 31.5 (s, C(*C*H$_3$)$_3$), 30.3 (s, *C*(CH$_3$)$_2$).

Elemental Analysis: Calculated for $C_{47}H_{49}NSb_2$: C 64.78, H 5.67, N 1.61; Found: C 64.50, H 5.53, N 1.69.

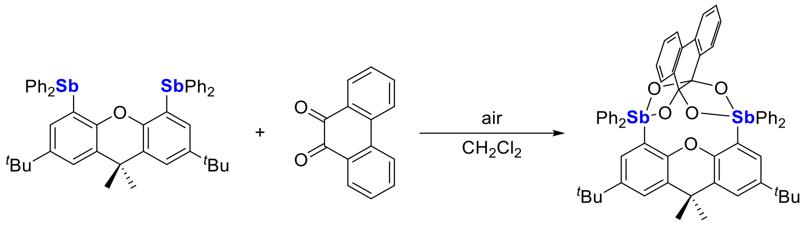

Compound 3_O: Compound **1_O** (100 mg, 0.115 mmol) and 9,10-phenanthraquinone (23.9 mg, 0.115 mmol) were combined in CH_2Cl_2 (5 mL). The resulting orange solution was stirred vigorously in the air for 2 h and subsequently brought to dryness under reduced pressure. The residue was triturated with pentane (20 mL) to afford **3_O** as a pale-yellow powder (106 mg, 0.0952 mmol, 83%). Single crystals suitable for X-ray diffraction were grown by vapor diffusion of Et_2O into a CH_2Cl_2 solution of **3_O** in a freezer (-20 °C).

^{1}H NMR ($CDCl_3$, 500.1 MHz): δ 8.07 (dd, $^3J_{H\text{-}H}$ = 7.6 Hz, $^4J_{H\text{-}H}$ = 1.6 Hz, 2H, Phenanthrene-*H*), 7.79 (dd, $^3J_{H\text{-}H}$ = 7.9 Hz, $^4J_{H\text{-}H}$ = 1.2 Hz, 2H, Phenanthrene-*H*), 7.50 (d, $^4J_{H\text{-}H}$ = 2.2 Hz, 2H, Xanthene-*H*), 7.39-7.34 (m, 8H, Ph-2,6-*H*), 7.31 (td, $^3J_{H\text{-}H}$ = 7.6 Hz, $^4J_{H\text{-}H}$ = 1.6 Hz, 2H, Phenanthrene-*H*), 7.28-7.23 (tt, $^3J_{H\text{-}H}$ = 7.4 Hz, $^4J_{H\text{-}H}$ = 1.4 Hz, 4H, Ph-4-*H*), 7.21 (td, $^3J_{H\text{-}H}$ = 7.5Hz, $^4J_{H\text{-}H}$ = 1.2 Hz, 2H, Phenanthrene-*H*), 7.16-7.10 (m, 8H, Ph-3,5-*H*), 6.89 (d, $^3J_{H,\,H}$ = 2.2 Hz, 2H, Xanthene-*H*), 1.66 (s, 6H, C(C*H*$_3$)$_2$), 1.15 (s, 18H, C(C*H*$_3$)$_3$).

^{13}C{^{1}H} NMR ($CDCl_3$, 125.8 MHz): δ 152.4 (s), 146.6 (s), 138.5 (s), 138.2 (s), 134.9 (s), 132.3 (s), 131.9 (s), 130.2 (s), 129.4 (s), 128.6 (s), 128.5 (s), 128.2 (s), 127.9 (s), 127.6 (s), 123.3 (s), 123.2 (s), 94.1 (s, O*C*O), 36.4 (s, C(*C*H$_3$)$_2$), 34.8 (s, *C*(CH$_3$)$_3$), 31.6 (s, C(*C*H$_3$)$_3$), 29.3 (s, *C*(CH$_3$)$_2$).

Elemental Analysis: Calculated for $C_{61}H_{56}O_5Sb_2$: C 65.85, H 5.07; Found: C 65.97, H 5.15.

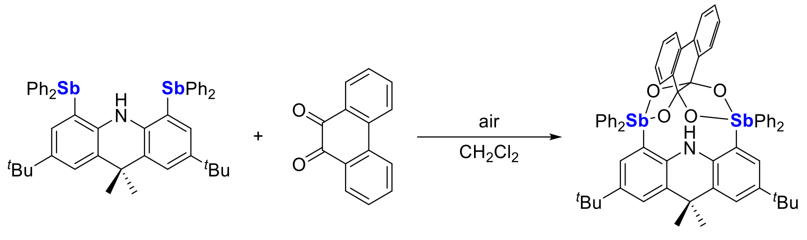

Compound 3_{NH}: Compound **1_{NH}** (200 mg, 0.230 mmol) and 9,10-phenanthraquinone (47.8 mg, 0.230 mmol) were combined in CH_2Cl_2 (10 mL). The resulting orange solution was stirred vigorously in the air for 2 h and subsequently brought to dryness under reduced pressure. The residue was triturated with pentane (20 mL) to afford **3_{NH}** as a pale-yellow powder (172 mg, 0.155 mmol, 67%).

^{1}H NMR ($CDCl_3$, 500.1 MHz): δ 11.72 (s, 1H, N-*H*), 7.68 (d, $^3J_{H\text{-}H}$=7.8 Hz, 2H, Phenanthrene-*H*), 7.65 (d, $^3J_{H\text{-}H}$=7.8 Hz, 2H, Phenanthrene-*H*), 7.50-7.39 (m, 10H, Ph-2,6-*H* & Xanthene-*H*), 7.30 (t, $^3J_{H\text{-}H}$ = 7.4 Hz, 4H, Ph-4-*H*), 7.24-7.18 (m, 8H, Ph-3,5-*H*), 7.16 (t, $^3J_{H\text{-}H}$ = 7.8 Hz, 2H, Phenanthrene-*H*), 6.96 (d, $^4J_{H\text{-}H}$ = 2.0 Hz, 2H, Xanthene-*H*), 6.83 (t, $^3J_{H\text{-}H}$ = 7.5 Hz, 2H, Phenanthrene-*H*), 1.67 (s, 6H, C(CH_3)$_2$, 1.13 (s, 18H, C(CH_3)$_3$.

^{13}C{^{1}H} NMR ($CDCl_3$, 125.8 MHz): δ 143.3 (s), 141.9 (s), 140.7 (s), 137.4 (s), 134.9 (s). 131.4 (s), 131.1 (s), 130.0 (s), 129.3 (s), 128.6 (s), 127.9 (s), 127.4 (s), 124.6 (s), 122.7 (s), 120.9 (s), 93.6 (s, O*C*O), 38.4 (s, C(*C*H_3)$_2$), 34.6 (s, *C*(CH_3)$_3$), 31.5 (s, C(*C*H_3)$_3$), 30.3 (s, *C*(CH_3)$_2$).

Elemental Analysis: Calculated for $C_{61}H_{57}NO_4Sb_2$: C 65.91, H 5.17, N 1.26; Found: C 65.82, H 5.29, N 1.38.

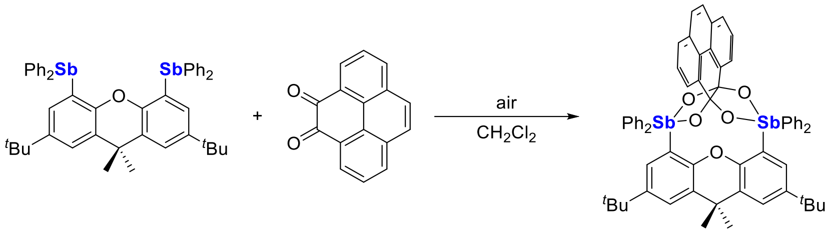

1O   4O

Compound 3O: Compound **1O** (200 mg, 0.229 mmol) and pyrene-4,5-dione (53.7 mg, 0.229 mmol) were combined in CH_2Cl_2 (10 mL). The resulting orange solution was stirred vigorously in the air for 2 h and subsequently brought to dryness under reduced pressure. The residue was triturated with pentane (20 mL) to afford **4O** as a pale-yellow powder (236 mg, 0.208 mmol, 91%). Single crystals suitable for X-ray diffraction were grown by slow vapor diffusion of pentane into a $CHCl_3$ solution of **4O** in a freezer (-20 °C).

^{1}H NMR ($CDCl_3$, 500.1 MHz): δ 8.31 (dd, $^3J_{H-H}$ = 7.3 Hz, $^4J_{H-H}$ = 1.2 Hz, 2H, Pyrene-3,6-*H*), 7.78 (dd, $^3J_{H-H}$ = 7.9 Hz, $^4J_{H-H}$ = 1.2 Hz, 2H, Pyrene-1,8-*H*), 7.72 (s, 2H, Pyrene-9,10-*H*), 7.54 (t, $^3J_{H-H}$ = 7.3 Hz, 2H, Pyrene-2,7-*H*), 7.53 (d, $^4J_{H-H}$ = 2.1 Hz, 2H, Xanthene-*H*), 7.43-7.39 (m, 8H, Ph-2,6-*H*), 7.25 (t, $^3J_{H-H}$ = 7.4 Hz, 4H, Ph-4-*H*), 7.12 (t, $^3J_{H-H}$ = 7.6 Hz, 8H, Ph-3,5-*H*), 6.90 (d, $^4J_{H-H}$ = 2.1 Hz, 2H, Xanthene-*H*), 1.69 (s, 6H, C(C*H*$_3$)$_2$), 1.16 (s, 18H, C(C*H*$_3$)$_3$).

^{13}C{^{1}H} NMR ($CDCl_3$, 125.8 MHz): δ 152.4(s), 146.6 (s), 138.4 (s), 138.2(s), 134.9 (s), 132.2(s), 131.2(s), 130.2(s), 129.4 (s), 128.6 (s), 127.6 (s), 127.2 (s), 126.9 (s), 126.5(s), 125.8(s), 125.2 (s), 123.3 (s), 94.8 (s, O*C*O), 36.4 (s, C(*C*H$_3$)$_2$), 34.8 (s, *C*(CH$_3$)$_3$), 31.6 (s, C(*C*H$_3$)$_3$), 29.4 (s, *C*(CH$_3$)$_2$).

Elemental Analysis: Calculated for $C_{63}H_{56}O_5Sb_2$: C 66.57, H 4.97; Found: C 66.56, H 4.92.

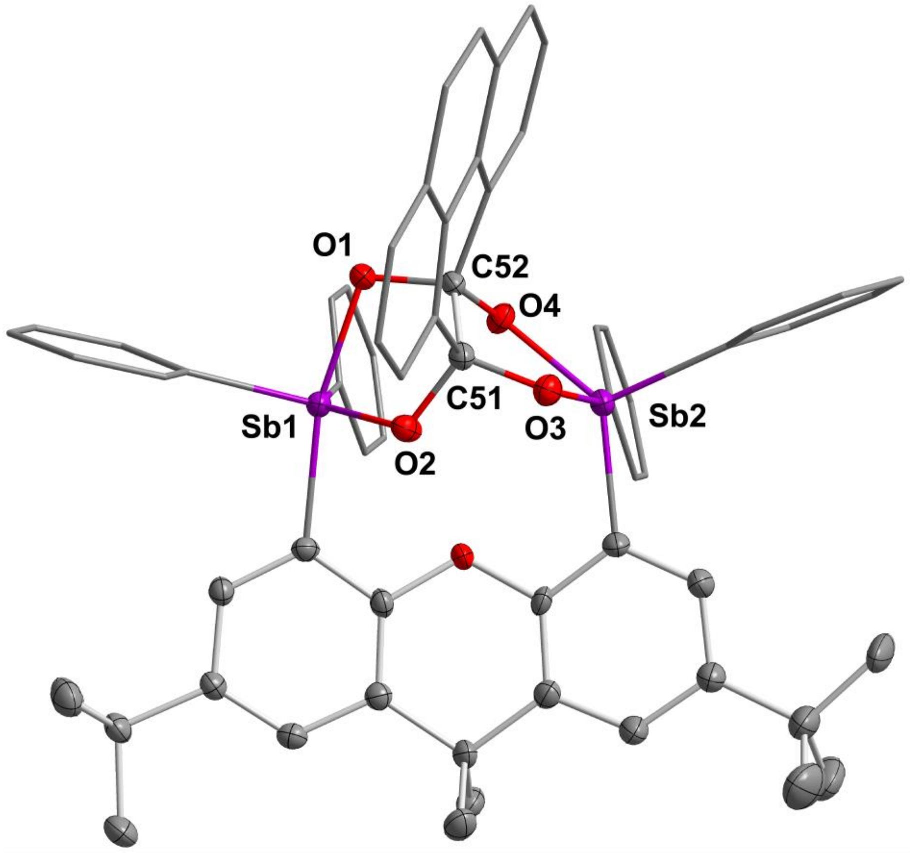

Figure S1. Crystal structure of **4O**.

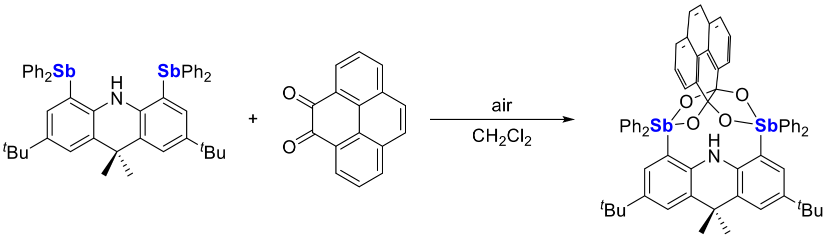

1_{NH} 4_{NH}

Compound 4_{NH}: Compound $\mathbf{1_{NH}}$ (200 mg, 0.230 mmol) and pyrene-4,5-dione (53.8 mg, 0.230 mmol) were combined with CH_2Cl_2 (10 mL). The resulting light green solution was stirred vigorously in the air for 2 h and subsequently brought to dryness under reduced pressure. The residue was triturated with pentane (20 mL), and then washed with Et_2O (20 mL) to afford $\mathbf{4_{NH}}$ as a pale-yellow powder (158 mg, 0.139 mmol, 60%). Single crystals suitable for X-ray diffraction were grown by slow vapor diffusion of pentane into a chlorobenzene solution of $\mathbf{4_{NH}}$.

^{1}H NMR ($CDCl_3$, 500.1 MHz): 11.81 (s, 1H, N-*H*), 7.85 (d, $^3J_{H-H}$ = 7.4 Hz, 2H, Pyrene-3,6-*H*), 7.67-7.61 (m, 4H, Pyrene-9,10-H & Pyrene-1,8-*H*), 7.47 (d, $^4J_{H-H}$ = 2.0 Hz, 2H, Acridine-*H*), 7.44-7.38 (m, 8H, Ph-2,6-*H*), 7.28 (t, $^3J_{H-H}$ = 7.2 Hz, Ph-4-*H*), 7.21-7.14 (m, 10H, Pyrene-2,7-*H* & Ph-3,5-*H*), 6.97 (d, $^4J_{H-H}$ = 2.0 Hz, 2H, Acridine-*H*), 1.69 (s, 6H, $C(CH_3)_2$), 1.13 (s, 18H, $C(CH_3)_3$).

^{13}C{^{1}H} NMR ($CDCl_3$, 125.8 MHz): δ 143.2 (s), 142.2(s), 140.9 (s), 137.1 (s), 134.9 (s), 131.5 (s), 130.7 (s), 130.1 (s), 129.4 (s), 128.6 (s), 127.0 (s), 126.9 (s), 126.4 (s), 126.3 (s), 124.9 (s), 124.6 (s), 120.2 (s), 94.3 (s, O*C*O), 38.4 (s, $C(CH_3)_2$), 34.6 (s, $C(CH_3)_3$), 31.5 (s, $C(CH_3)_3$), 30.0 (s, $C(CH_3)_2$).

Elemental Analysis: Calculated for $C_{63}H_{57}NO_4Sb_2$: C 66.63, H 5.06, N 1.23; Found: C 66.83, H 5.08, N 1.25.

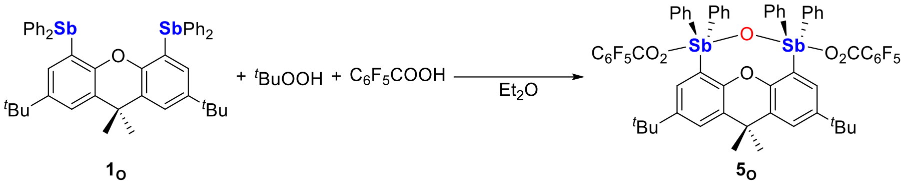

Compound 5$_O$: An aqueous solution of *tert*-butyl hydrogen peroxide (70%, 0.10 mL) was slowly added to a solution of **1$_O$** (200 mg, 0.229 mmol) and C_6F_5COOH (97.2 mg, 0.458 mmol) in Et_2O (10 mL). The resulting mixture was stirred at room temperature overnight and subsequently brought to dryness under reduced pressure. The resulting residue was dissolved in CH_2Cl_2 (10 mL) to afford a solution that was washed with H_2O (10 mL × 3) in order to remove residual *tert*-butyl hydrogen peroxide. The organic phase was dried over anhydrous $MgSO_4$, filtered, and then brought to dryness. The residue was triturated with hexanes (10 mL) to give compound **5$_O$** as a white solid (190 mg, 63%). Single crystals suitable for X-ray diffraction were grown by slowly evaporating a solution of **5$_O$** in a mixture of $CHCl_3$/benzene/hexanes.

^{1}H NMR ($CDCl_3$, 500.1 MHz): 7.95 (d, $^4J_{H\text{-}H}$ = 2.0 Hz, 2H, Xanthene-*H*), 7.77-7.73 (m, 8H, Ph-2,6-*H*), 7.61 (d, $^4J_{H\text{-}H}$ = 2.0 Hz, 2H, Xanthene-*H*), 7.32 (t, $^3J_{H\text{-}H}$ = 7.4 Hz, 4H, Ph-4-*H*), 7.23-7.18 (m, 8H, Ph-3,5-*H*), 1.83 (s, 6H, $C(CH_3)_2$), 1.28 (s, 18H, $C(CH_3)_3$).

^{13}C{^{1}H} NMR ($CDCl_3$, 125.8 MHz): δ 160.7 (s, $C_6F_5CO_2$), 156.0 (s), 149.1 (s), 144.2 (dm, $^1J_{C\text{-}F}$ = 250.8 Hz, C_6F_5), 141.2 (dm, $^1J_{C\text{-}F}$ = 254.3 Hz, C_6F_5), 139.8 (s), 137.4 (dm, $^1J_{C\text{-}F}$ = 252.1 Hz, C_6F_5), 136.9 (s), 133.5 (s), 130.8 (s), 130.2 (s), 129.2 (s), 125.0 (s), 123.5 (s), 113.8 (t, $^2J_{C\text{-}F}$ = 19.3 Hz, C_6F_5-*ipso*-*C*), 38.6 (s, $C(CH_3)_2$), 35.3(s, $C(CH_3)_3$), 31.5 (s, $C(CH_3)_3$), 26.9 (s, $C(CH_3)_2$).

^{19}F NMR ($CDCl_3$, 470.4 MHz): δ -141.6 (dd, $^3J_{F\text{-}F}$ = 22.8 Hz, $^4J_{F\text{-}F}$ = 8.1 Hz, 2F, C_6F_5-2,6-*F*). -155.1 (t, $^3J_{F\text{-}F}$ = 20.6 Hz, 1F, C_6F_5-4-*F*), -162.5 (m, 2F, C_6F_5-3,5-*F*).

HRMS (ESI+): Calculated m/z for $C_{54}H_{48}F_5O_4Sb_2^+$ ([M-C_6F_5COO]$^+$), 1097.1544; Found, 1097.1529.

2.2 NMR spectra

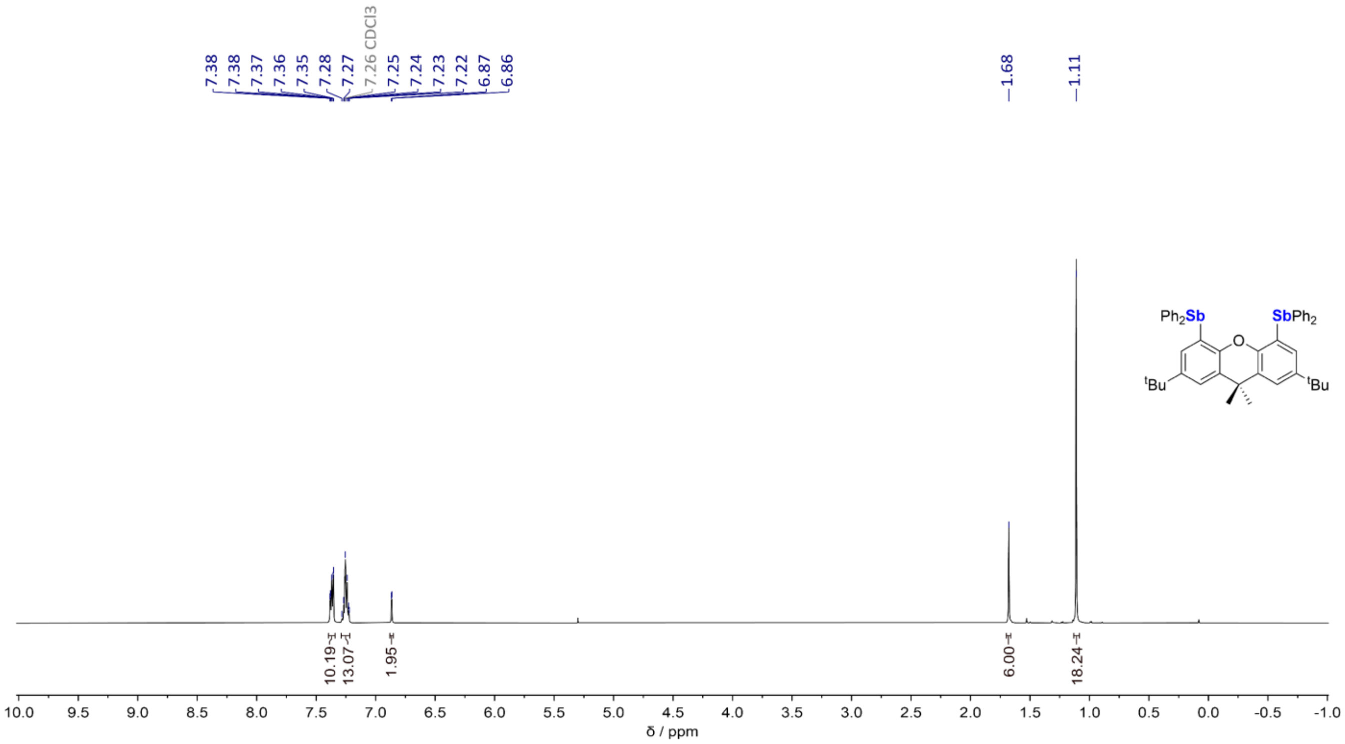

Figure S2. ^{1}H NMR ($CDCl_3$, 500.1 MHz) spectrum of $\mathbf{1_O}$.

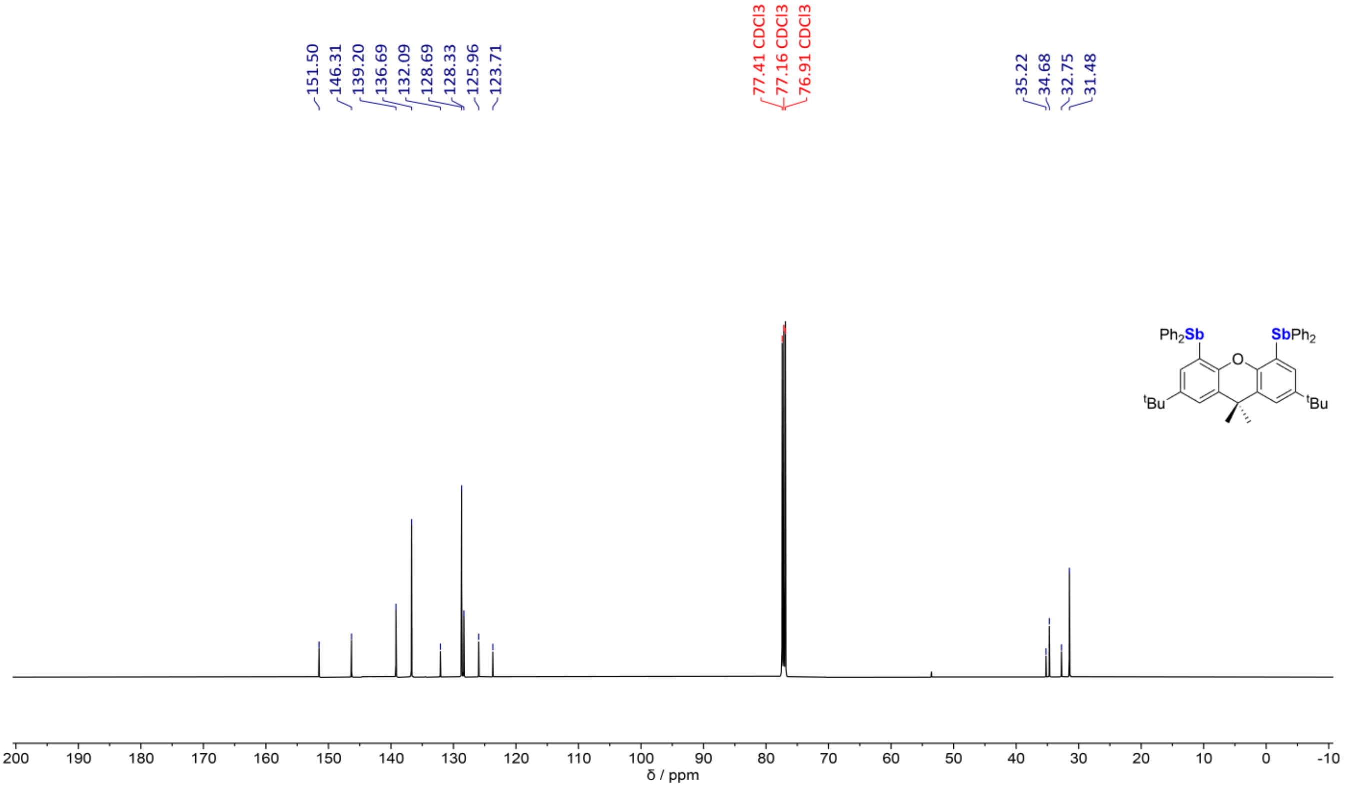

Figure S3. $^{13}C\{^{1}H\}$ NMR ($CDCl_3$, 125.8 MHz) spectrum of $\mathbf{1_O}$.

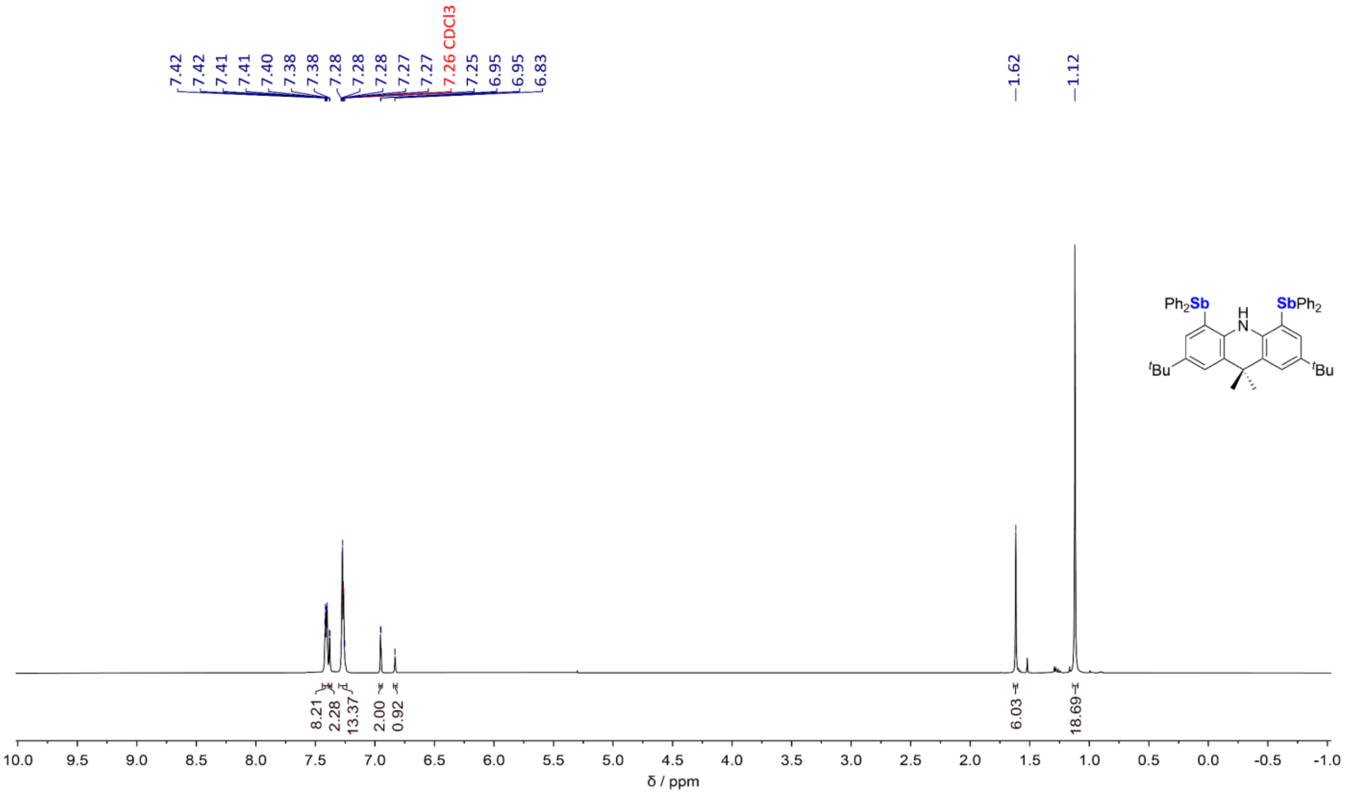

Figure S4. ^{1}H NMR ($CDCl_3$, 500.1 MHz) spectrum of $\mathbf{1_{NH}}$.

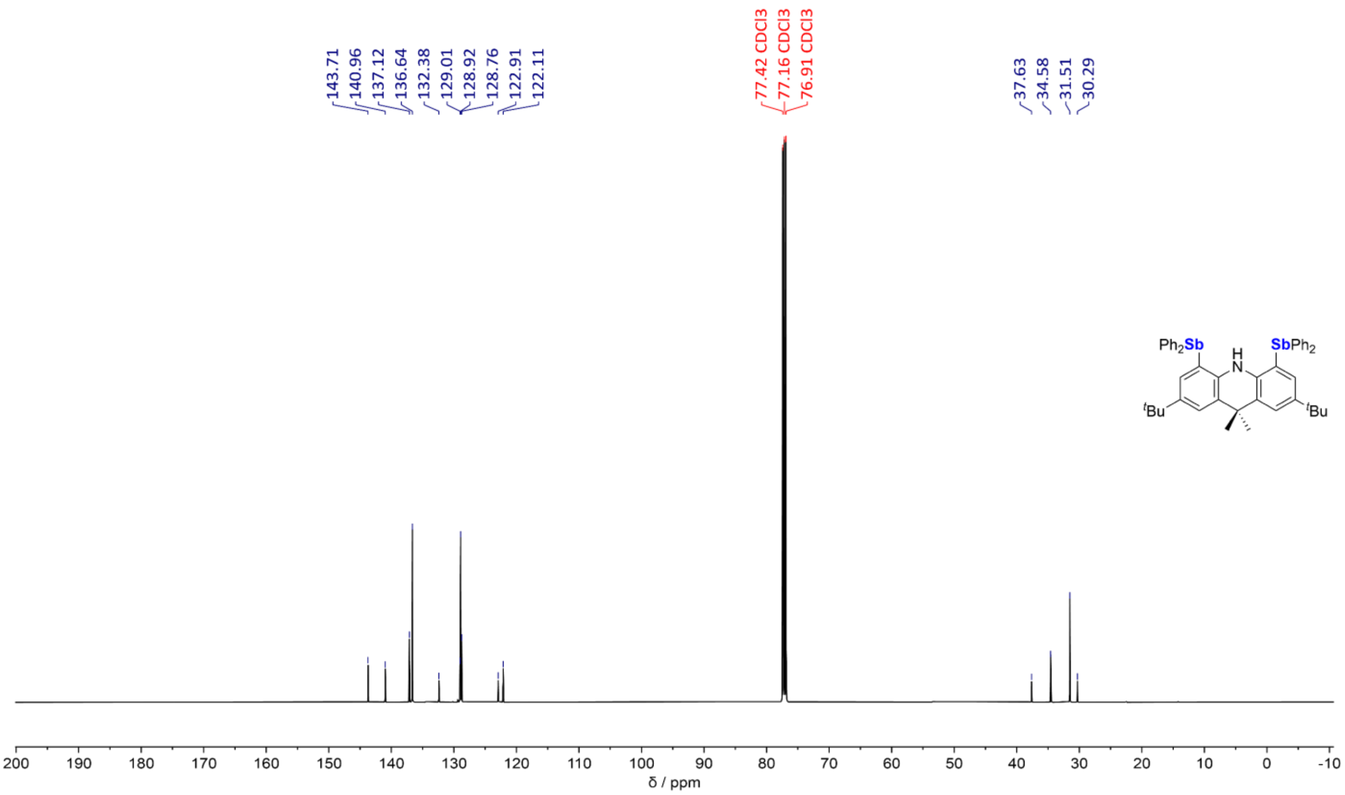

Figure S5. $^{13}C\{^{1}H\}$ NMR ($CDCl_3$, 125.8 MHz) spectrum of $\mathbf{1_{NH}}$.

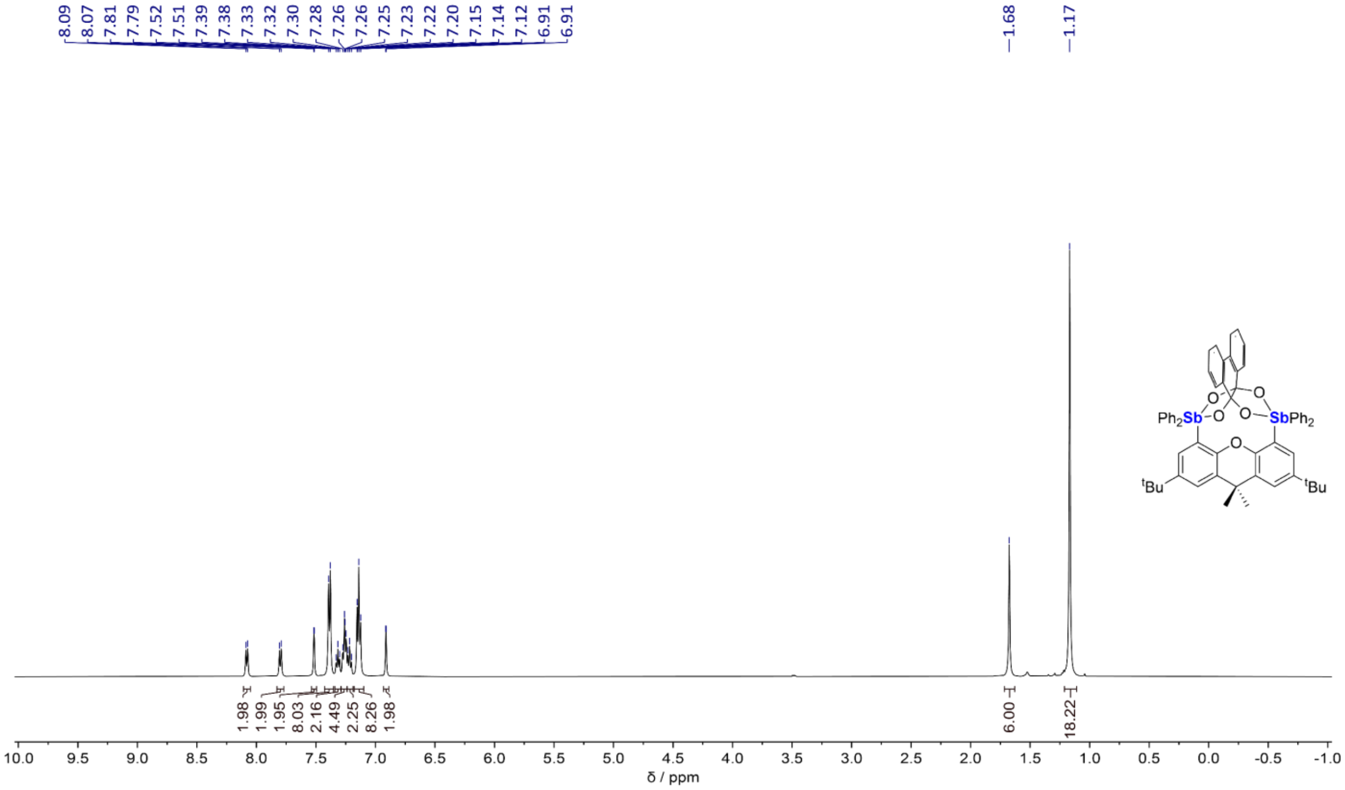

Figure S6. ^{1}H NMR ($CDCl_3$, 500.1 MHz) spectrum of $\mathbf{3_O}$.

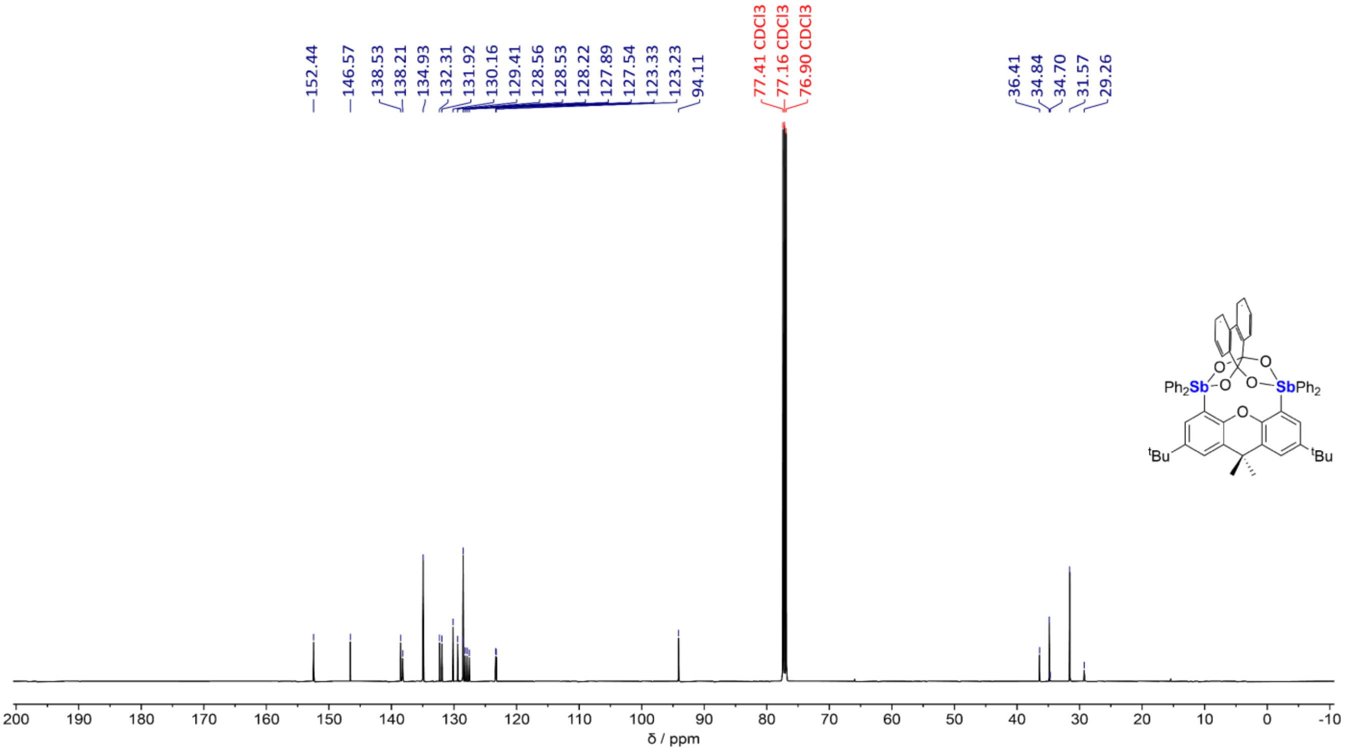

Figure S7. ^{13}C{^{1}H} NMR ($CDCl_3$, 125.8 MHz) spectrum of $\mathbf{3_O}$.

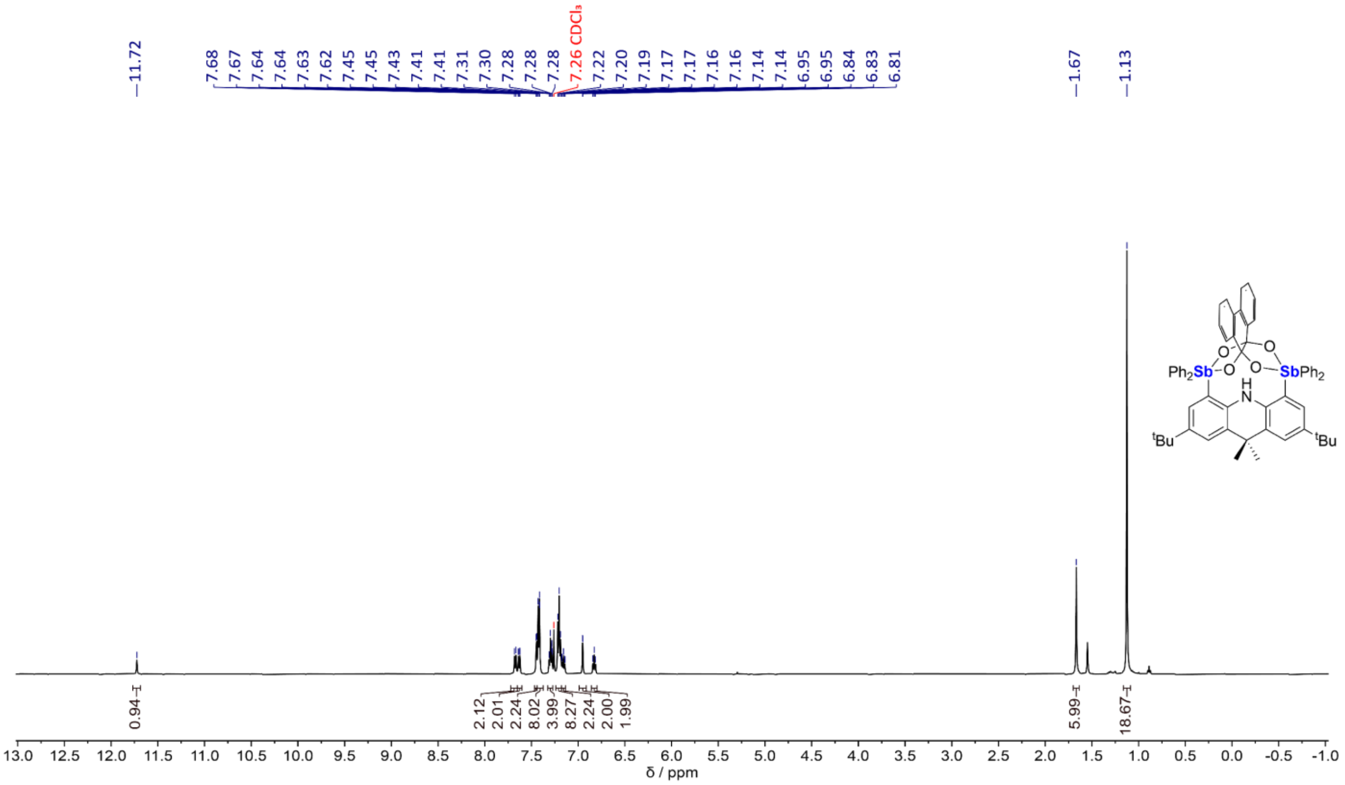

Figure S8. ^{1}H NMR ($CDCl_3$, 500.1 MHz) spectrum of $\mathbf{3_{NH}}$.

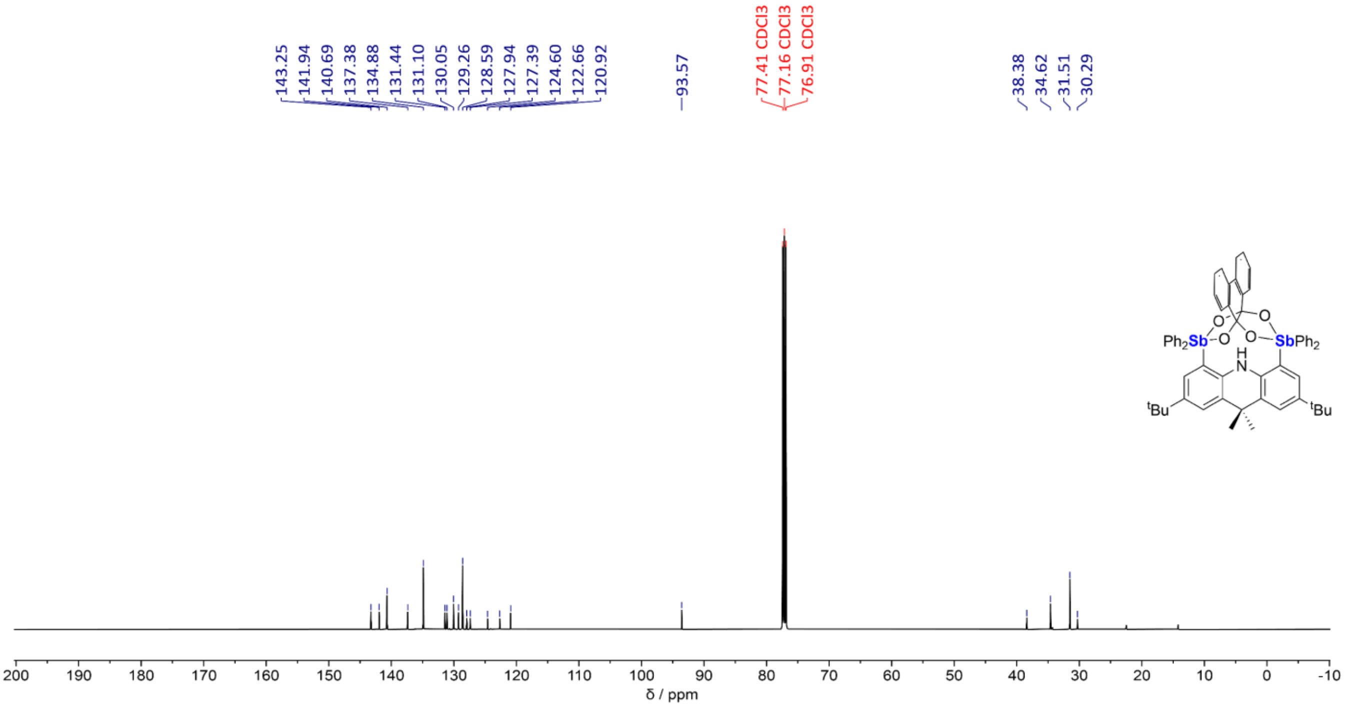

Figure S9. ^{13}C{^{1}H} NMR ($CDCl_3$, 125.8 MHz) spectrum of $\mathbf{3_{NH}}$.

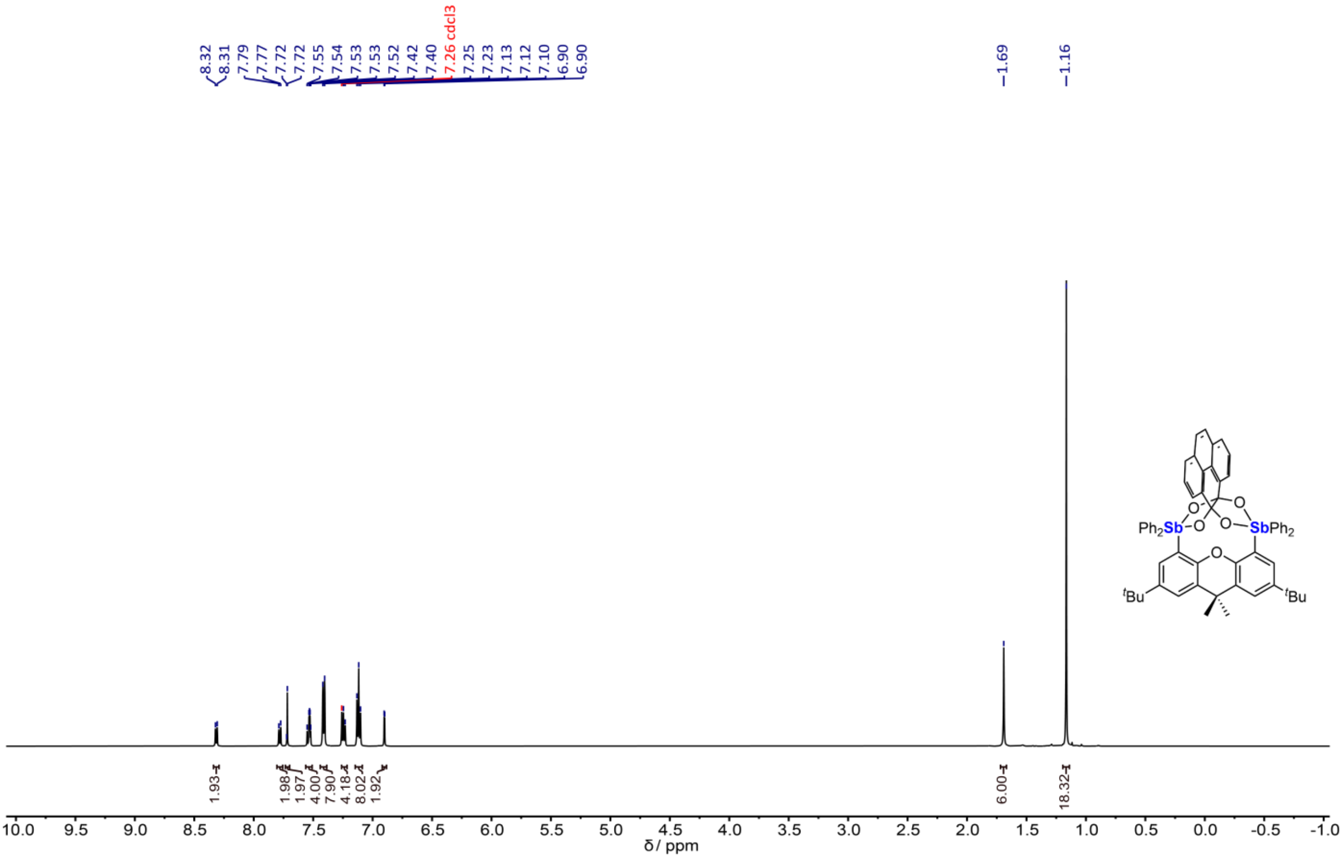

Figure S10. ^{1}H NMR ($CDCl_3$, 500.1 MHz) spectrum of **4$_{O}$**.

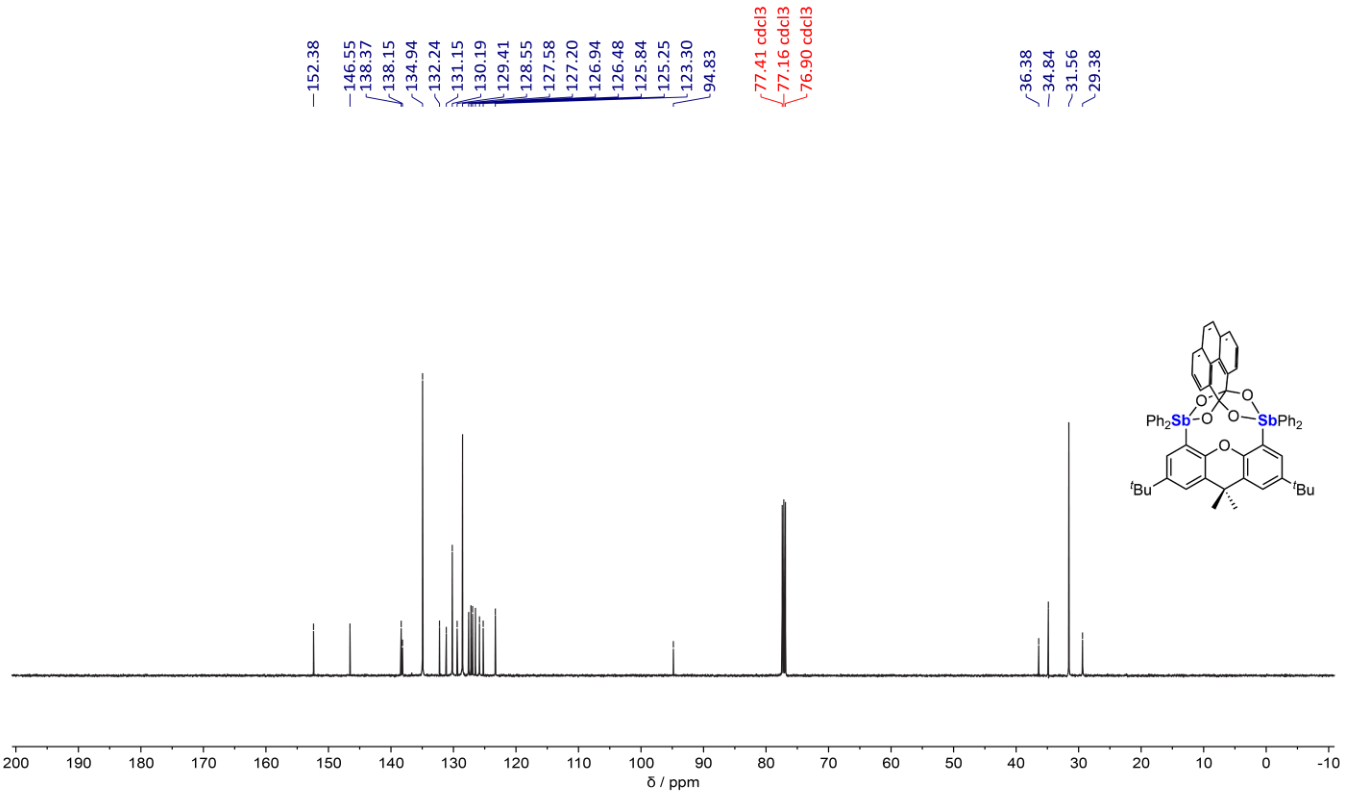

Figure S11. ^{13}C{^{1}H} NMR ($CDCl_3$, 125.8 MHz) spectrum of **4$_{O}$**.

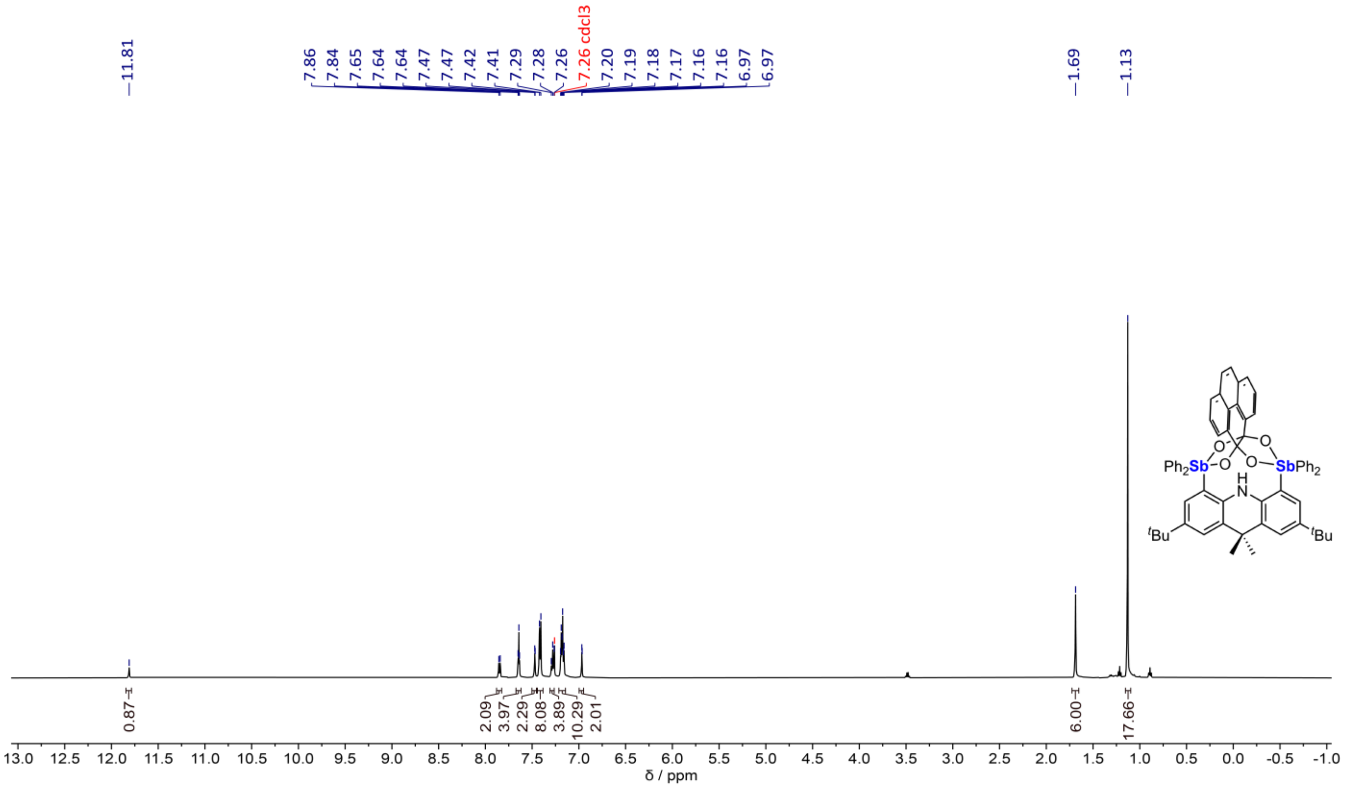

Figure S12. ^{1}H NMR ($CDCl_3$, 500.1 MHz) spectrum of $\mathbf{4_{NH}}$.

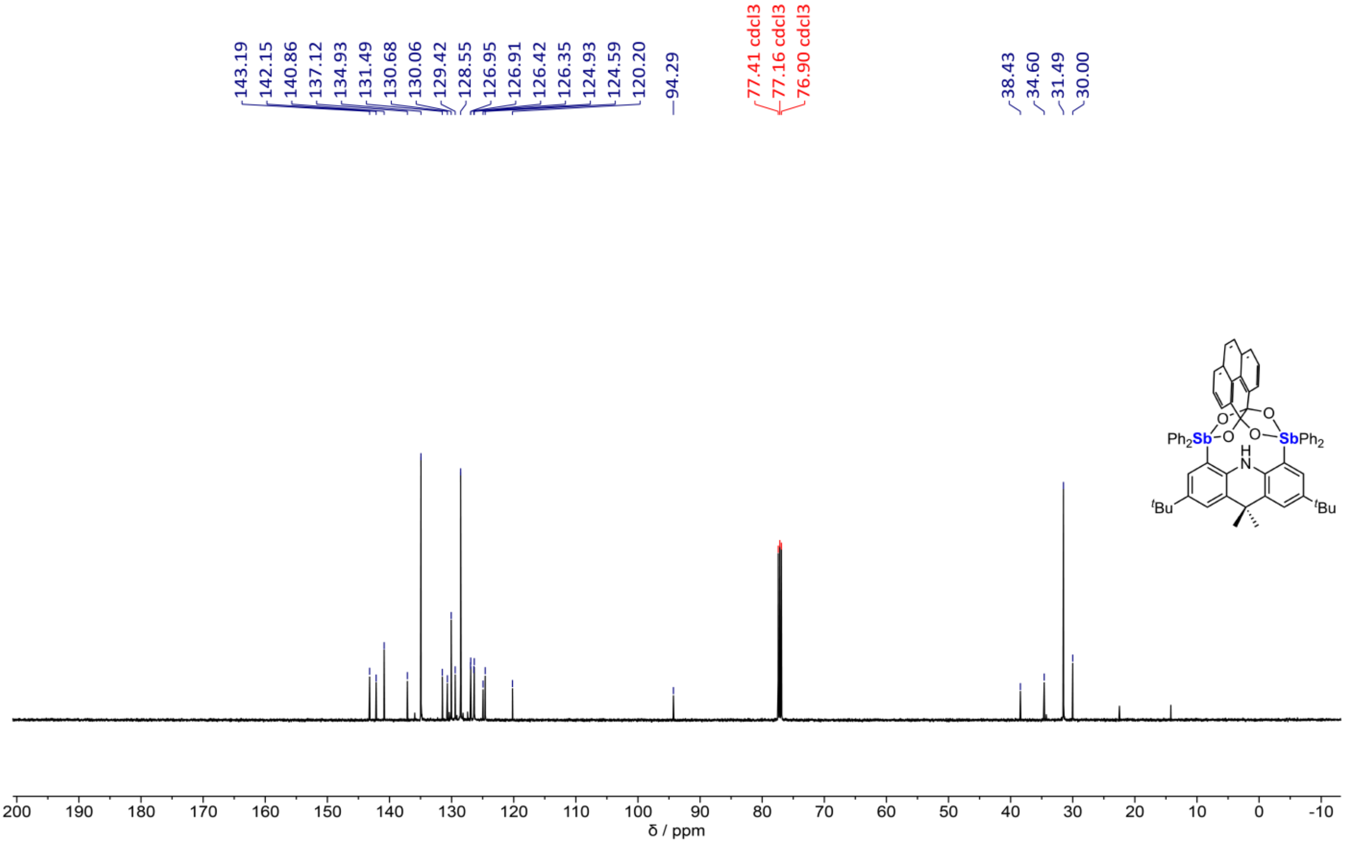

Figure S13. ^{13}C{^{1}H} NMR ($CDCl_3$, 125.8 MHz) spectrum of $\mathbf{4_{NH}}$.

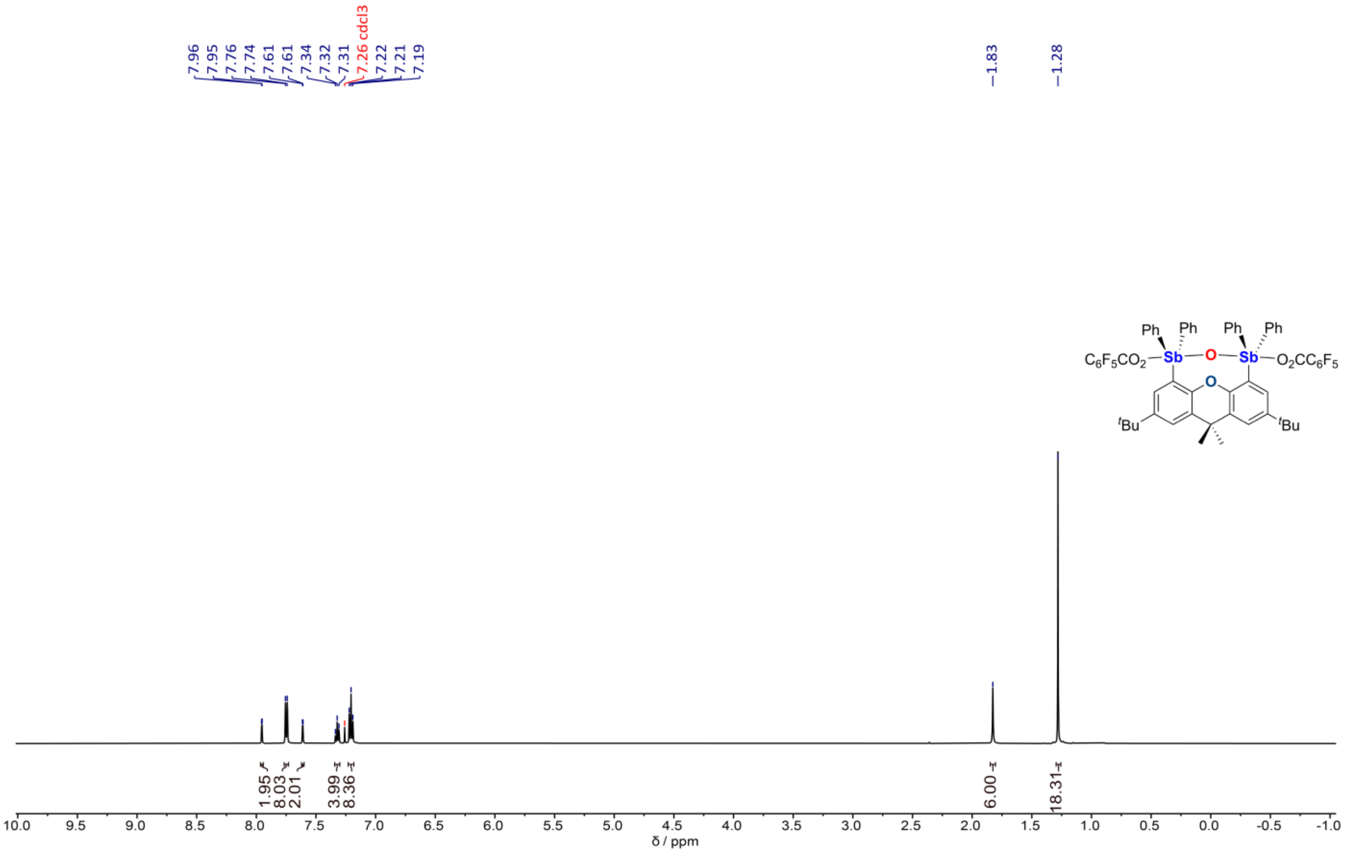

Figure S14. ^{1}H NMR ($CDCl_3$, 500.1 MHz) spectrum of **5**$_{\mathbf{O}}$.

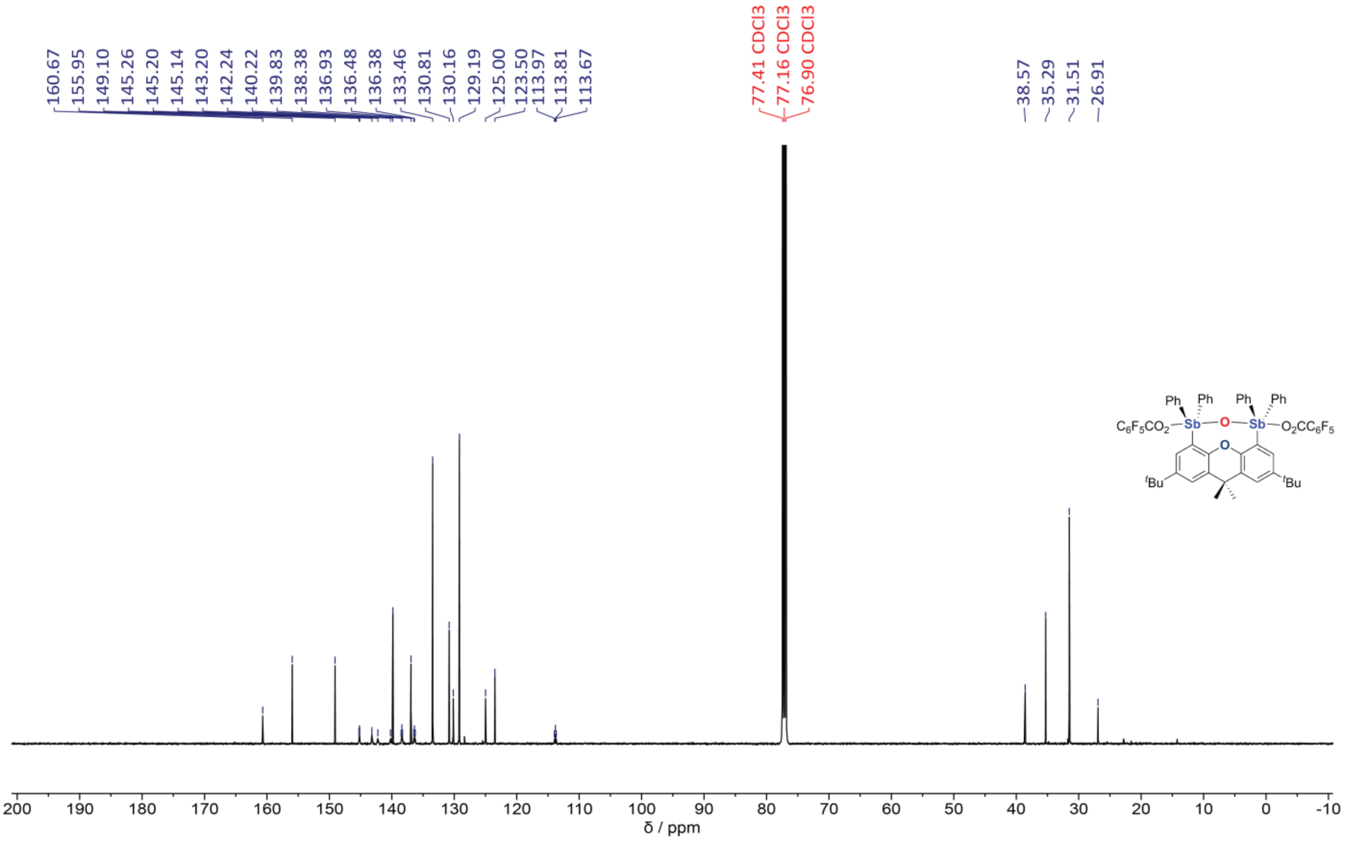

Figure S15. ^{13}C{^{1}H} NMR ($CDCl_3$, 125.8 MHz) spectrum of **5**$_{\mathbf{O}}$.

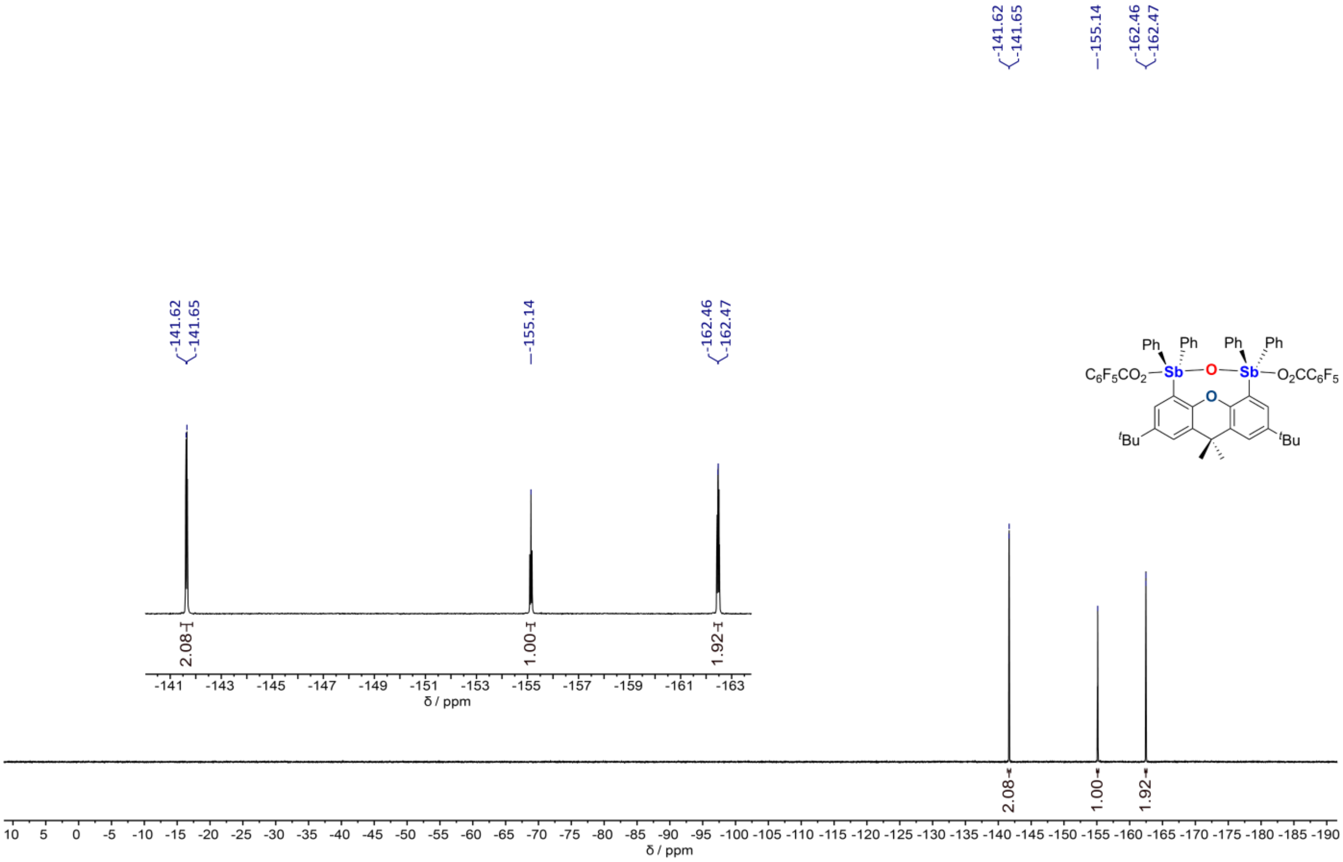

Figure S16. ^{19}F NMR ($CDCl_3$, 470.4 MHz) spectrum of **5_O**.

3 Detection of $\mathbf{2_O}$ and $\mathbf{2_{NH}}$ via *in situ* ^{1}H NMR and determination of their formation constants.

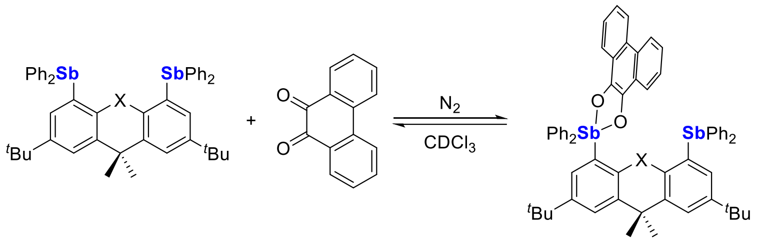

$\mathbf{1_O}$, X = O
$\mathbf{1_{NH}}$, X = NH

phenQ

$\mathbf{2_O}$, X = O
$\mathbf{2_{NH}}$, X = NH

Distibine $\mathbf{1_O}$ (17.6 mg, 0.0202 mmol) or $\mathbf{1_{NH}}$ (17.6 mg, 0.0202 mmol) and varying amounts of 9,10-phenanthraquinone (4.2 mg, 0.020 mmol, 1 equiv.; 8.4 mg, 0.040 mmol, 2 equiv.; 12.6 mg, 0.0605 mmol, 3 equiv.; 21.0 mg, 0.101 mmol, 5 equiv.) were dissolved in $CDCl_3$ (0.5 mL) in a J. Young NMR tube under an N_2 atmosphere. Solutions of $\mathbf{1_O}$ and phenQ are orange in color while the solutions of $\mathbf{1_{NH}}$ and phenQ are dark green. ^{1}H NMR spectra of these solutions were recorded (see **Figure S17** and **Figure S18**). Integrals of the aliphatic peaks arising from the methyl or ^{t}Bu groups of $\mathbf{1_O}$ (or $\mathbf{1_{NH}}$) and $\mathbf{2_O}$ (or $\mathbf{2_{NH}}$) allowed for the determination of equilibrium concentration of these two species, denoted as [$\mathbf{1_O}$] (or [$\mathbf{1_{NH}}$]) and [$\mathbf{2_O}$] (or [$\mathbf{2_{NH}}$]). The concentrations of 9,10-phenanthraquinone at equilibrium ([phenQ]) were calculated by subtracting its initial concentrations by [$\mathbf{2_O}$] (or [$\mathbf{2_{NH}}$]). The results are summarized in **Table S1.**

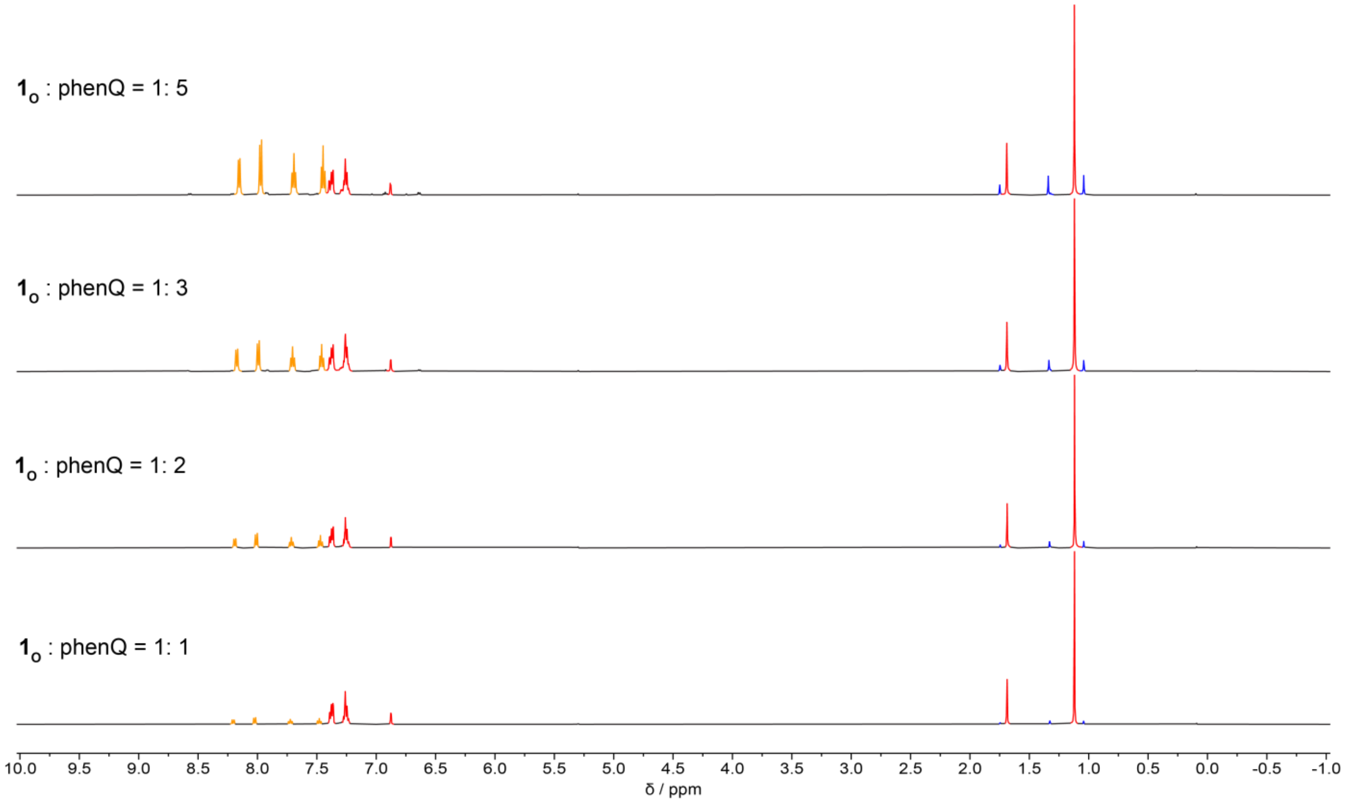

Figure S17. ^{1}H NMR ($CDCl_3$, 500.1 MHz) spectra of 1:x (x = 1, 2, 3, and 5) mixtures of $\mathbf{1_O}$ and 9,10-phenanthraquinone under an N_2 atmosphere: red trace, $\mathbf{1_O}$; orange trace, 9,10-phenanthraquinone; blue trace, $\mathbf{2_O}$.

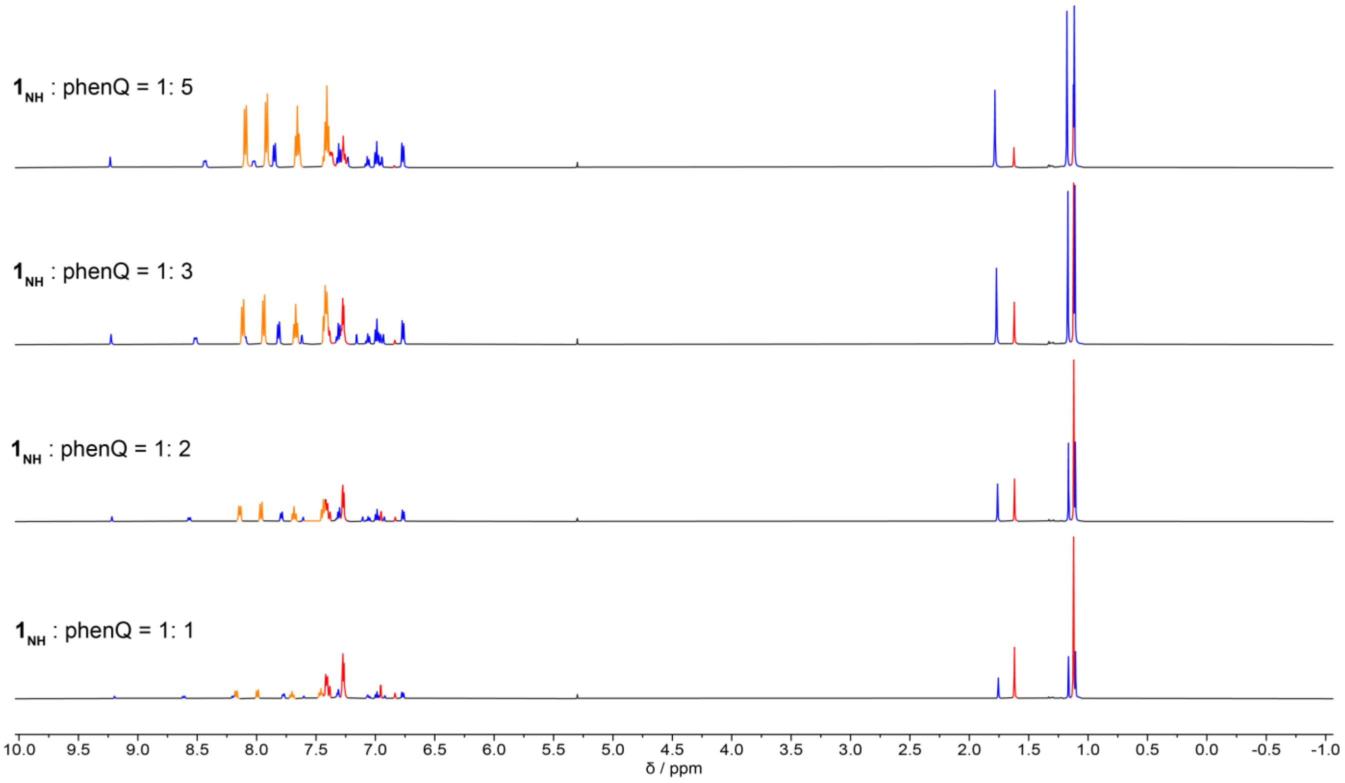

Figure S18. ^{1}H NMR ($CDCl_3$, 500.1 MHz) spectra of 1:x (x = 1, 2, 3, and 5) mixtures of $\mathbf{1_{NH}}$ and 9,10-phenanthraquinone under an N_2 atmosphere: red trace, $\mathbf{1_{NH}}$; orange trace, 9,10-phenanthraquinone; blue trace, $\mathbf{2_{NH}}$.

Table S1. Summarized results for the determination of formation constants for **2**$_{O}$ or **2**$_{NH}$

Mixture	Ratio	[**1**$_{O}$] or [**1**$_{NH}$] / mM	[**2**$_{O}$] or [**2**$_{NH}$] / mM	[phenQ] / mM	K / M^{-1}	$K_{average}$ / M^{-1}	σ
**1**$_{O}$ + phenQ	1 : 1	38.6	1.8	38.6	1.2	1.2	0.13
	1 : 2	37.5	2.9	77.9	1.0		
	1 : 3	35.1	5.3	115.9	1.3		
	1 : 5	32.7	7.7	194.3	1.2		
**1**$_{NH}$ + phenQ	1 : 1	28.3	12.1	28.3	15.1	19.1	5.0
	1 : 2	20.9	19.5	61.3	15.2		
	1 : 3	13.6	26.8	95.4	20.6		
	1 : 5	7.6	32.8	169.2	25.5		

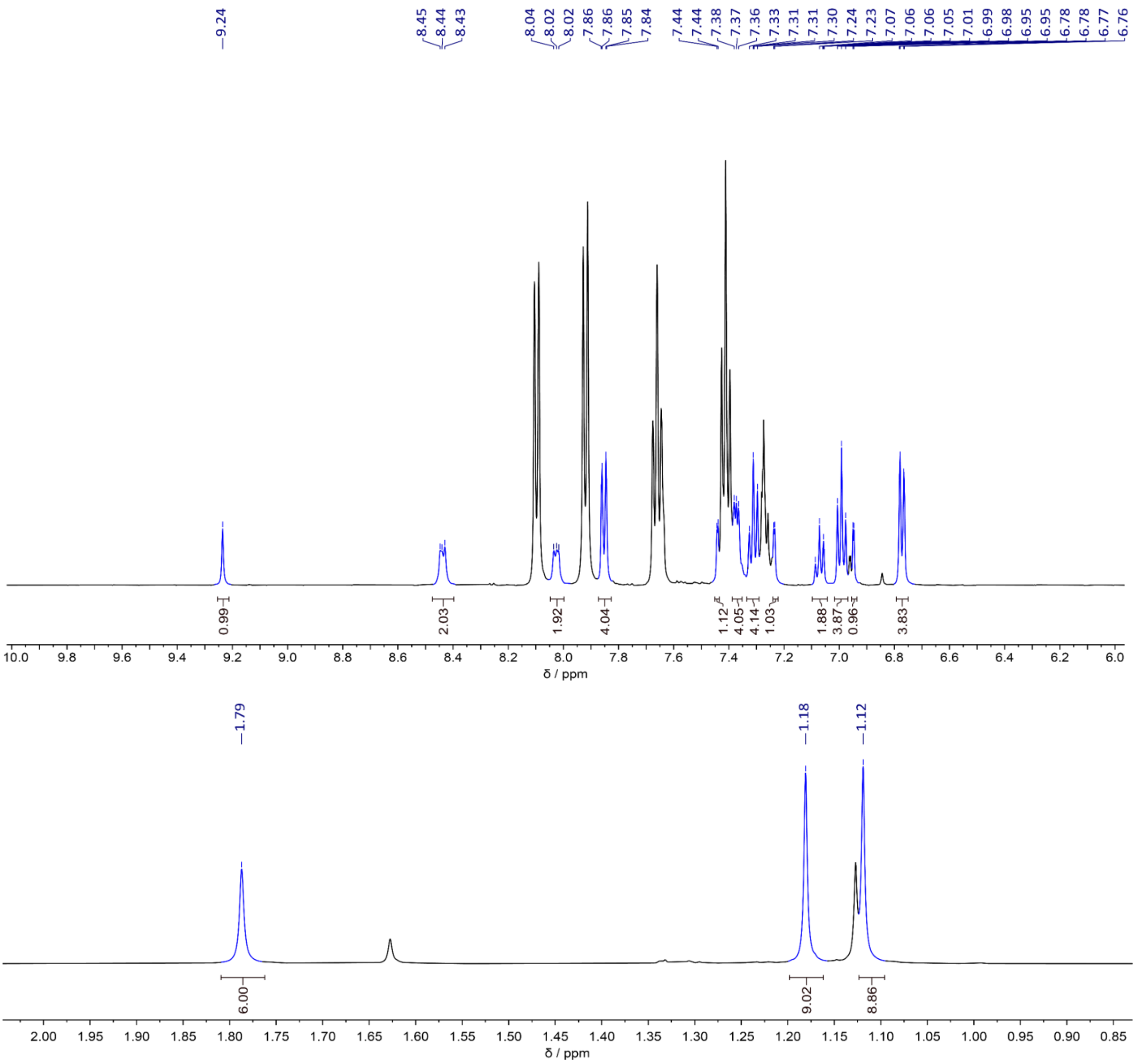

Figure S19. Portions of the ^{1}H NMR ($CDCl_3$, 500.1 MHz) spectrum of a 1:5 mixture of $\mathbf{1}_{\mathbf{NH}}$ and 9,10-phenanthraquinone under an N_2 atmosphere: blue trace, $\mathbf{2}_{\mathbf{NH}}$. Peaks are assigned as follows: δ 9.24 (s, 1H, N*H*), 8.47-8.40 (m, 2H, Phenanthrene-*H*), 8.05-8.00 (m, 2H, Phenanthrene-*H*), 7.87-7.83 (m, 4H, Ph-2,6-*H*), 7.44 (d, $^{4}J_{H\text{-}H}$ = 2.0 Hz, 1H, Acridine-*H*), 7.39-7.35 (m, 4H, Phenanthrene-*H* & Ph-4-*H*), 7.33-7.29 (m, 4H, Ph-3,5-*H*), 7.24 (d, $^{4}J_{H\text{-}H}$ = 2.0 Hz, 1H, Acridine-*H*), 7.09-7.05 (m, 2H, Phenanthrene-*H*), 7.02-6.97 (m, 4H, Ph-3,5-*H*), 6.95 (d, $^{4}J_{H\text{-}H}$ = 2.0 Hz, 1H, Acridine-*H*), 6.79-6.75 (m, 4H, Ph-3,5-*H*), 1.79 (s, 6H, C(CH_3)$_2$), 1.18 (s, 9H, C(CH_3)$_3$), 1.12 (s, 9H, C(CH_3)$_3$). Two protons from the Ph groups and one proton from the acridine backbone are missing, likely buried under the peaks of free 9,10-phenanthraquinone.

4 Formation of 3_O and 3_{NH} under O_2 atmosphere detected via *in situ* ^{1}H NMR

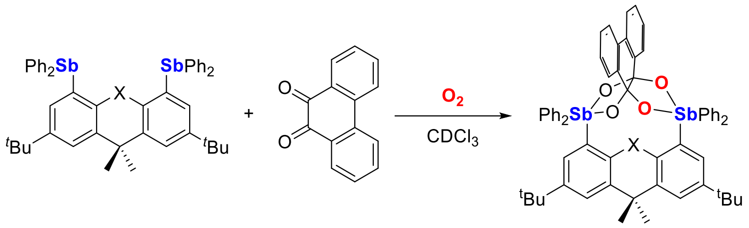

1$_O$, X = O
1$_{NH}$, X = NH

phenQ

3$_O$, X = O
3$_{NH}$, X = NH

In an N_2 glove box, a J. Young NMR tube was charged with **1**$_O$ (17.6 mg, 0.0202 mmol) or **1**$_{NH}$ (17.6 mg, 0.0202 mmol), 9,10-phenanthraquinone (4.2 mg, 0.020 mmol), and $CDCl_3$ (0.5 mL). The NMR tube was then taken out of the glove box and the solution phase was cooled down to -78 °C. After the solution was completely frozen, the gas phase above the frozen solution was evacuated, and then backfilled with O_2 (25 psi). The solution was thawed to room temperature and the ^{1}H NMR spectra were recorded periodically. The results are summarized in **Figure S20** and **Figure S21**.

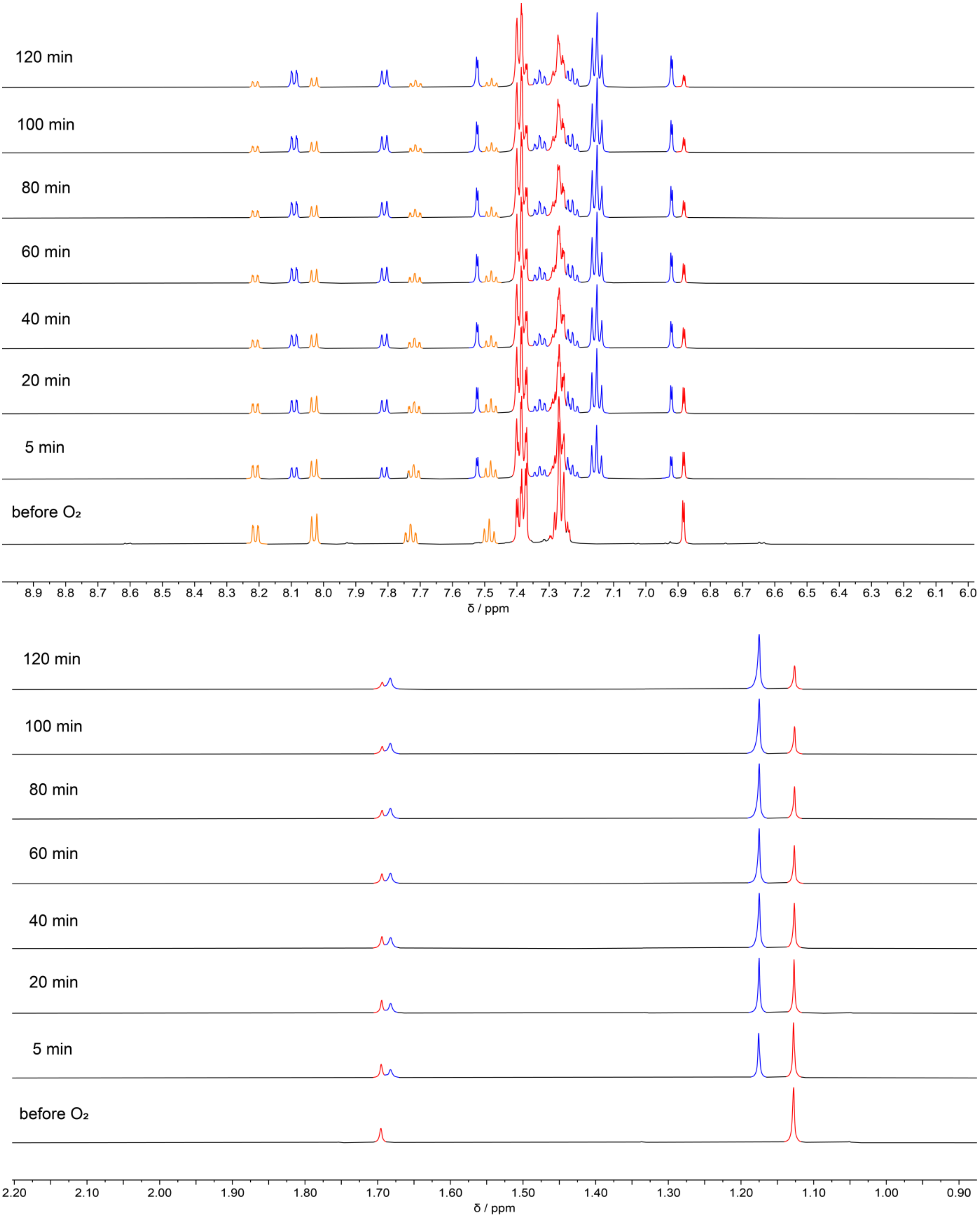

Figure S20. Portions of ^{1}H NMR ($CDCl_3$, 500.1 MHz) spectra of a 1:1 mixture of $\mathbf{1_O}$ and 9,10-phenanthraquinone under an O_2 atmosphere: red trace, $\mathbf{1_O}$; orange trace, 9,10-phenanthraquinone; blue trace, $\mathbf{3_O}$.

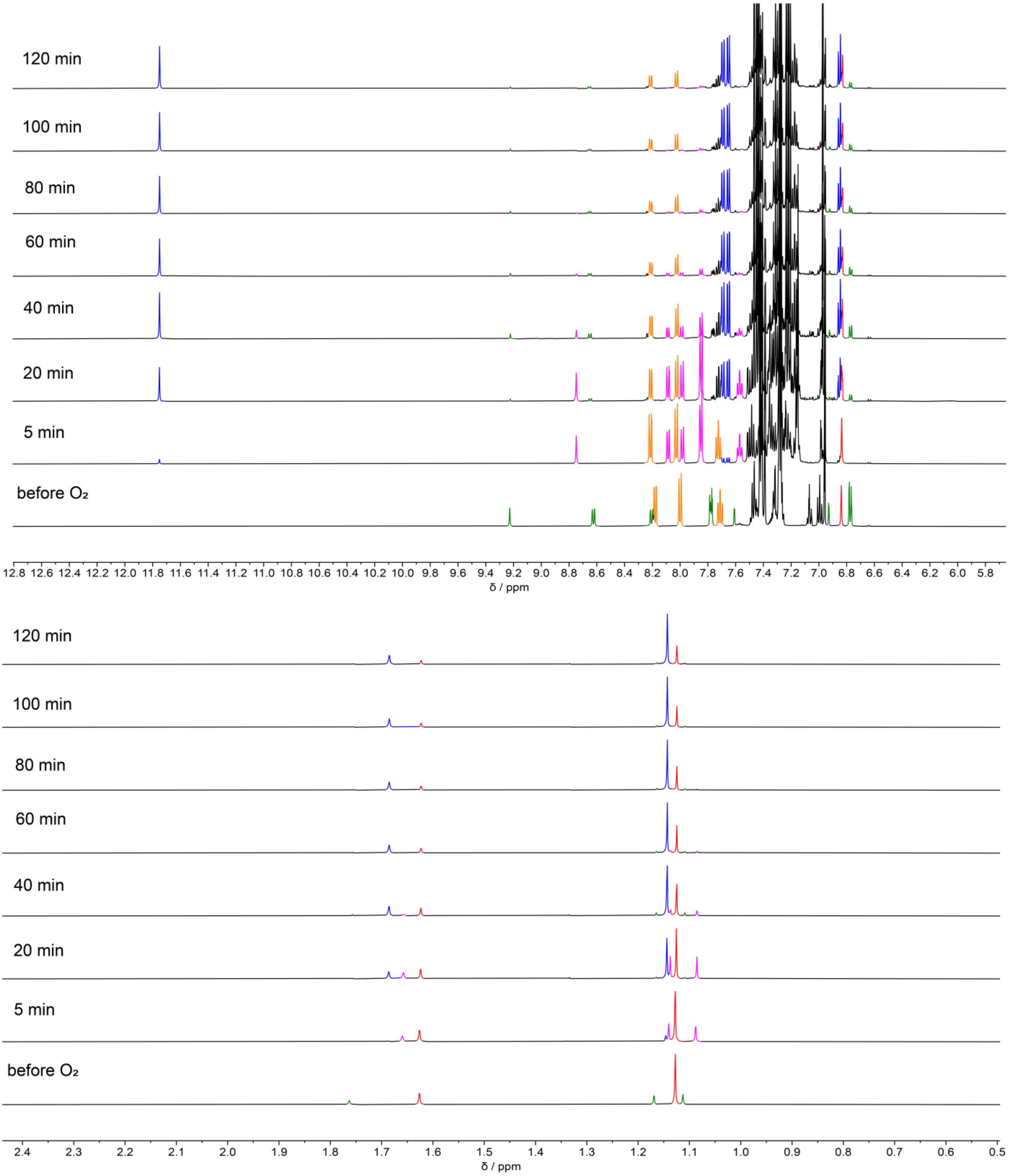

Figure S21. Portions of ^{1}H NMR ($CDCl_3$, 500.1 MHz) spectra of a 1:1 mixture of $\mathbf{1_O}$ and 9,10-phenanthraquinone under an O_2 atmosphere: red trace, $\mathbf{1_{NH}}$; orange trace, 9,10-phenanthraquinone; blue trace, $\mathbf{3_{NH}}$, green trace, $\mathbf{2_{NH}}$; magenta trace, $\mathbf{Int_{NH}}$.

5 ^{18}O isotope labeling study using $^{18}O_2$ as the oxygen source.

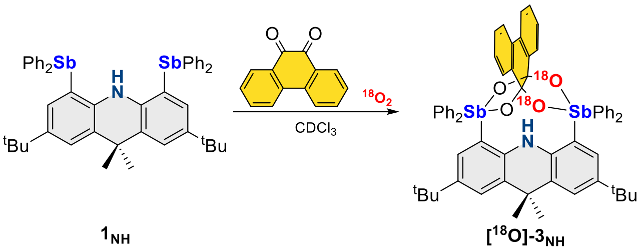

In an N_2 glove box, a J-Young NMR tube was charged with **1_{NH}** (30.0 mg, 0.0344 mmol), 9,10-phenanthraquinone (7.2 mg, 0.034 mmol), and $CDCl_3$ (0.5 mL), affording a dark green solution. The NMR tube was then taken out of the glove box and the solution phase was cooled down to -78 °C. After the solution was completely frozen, the J-Young NMR tube was evacuated to remove the N_2 atmosphere and backfilled with $^{18}O_2$ (97% ^{18}O enrichment, 25 psi). The frozen solution was thawed to room temperature, and then the NMR tube was inverted a couple of times to ensure sufficient mixing. An immediate color change from dark green to orange was seen after the mixing. Full conversion of **1_{NH}** into **[^{18}O]-3_{NH}** was confirmed the next day via ^{1}H NMR spectroscopy, which showed the same signals as those of **3_{NH}** (**Figure S22**). The resulting solution was directly subjected to mass spectrometry analysis using Electrospray Ionization in the negative mode (ESI$^-$). The anion arising from the deprotonation of **[^{18}O]-3_{NH}** was detected with a calculated monoisotopic mass of 1112.2365, while the same ESI$^-$ analysis of compound **3_{NH}** yielded a monoisotopic mass of 1108.2280 (**Figure S23** and **Figure S24**).

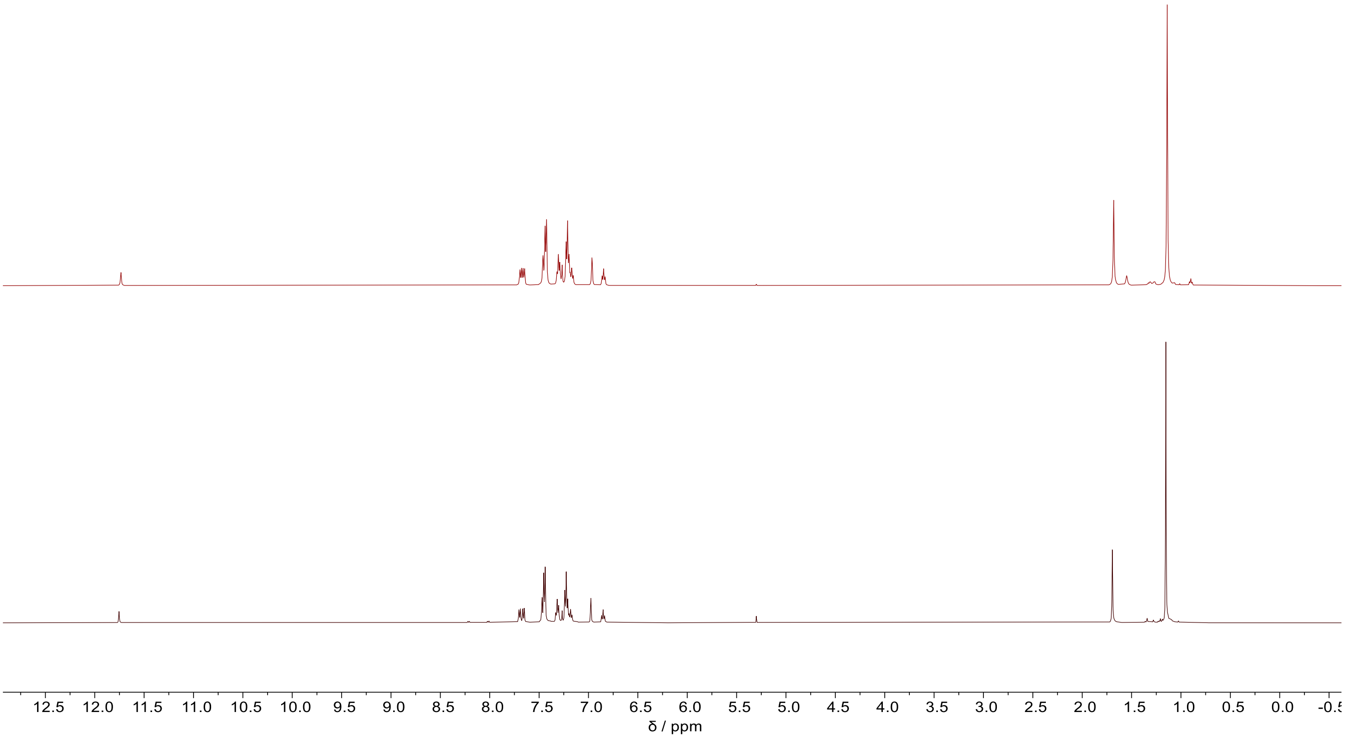

Figure S22. ^{1}H NMR ($CDCl_3$, 500.1 MHz) spectra of **3_{NH}** (top) and the reaction mixture of **1_{NH}** and 9,10-phenanthraquinone under an $^{18}O_2$ atmosphere overnight (bottom).

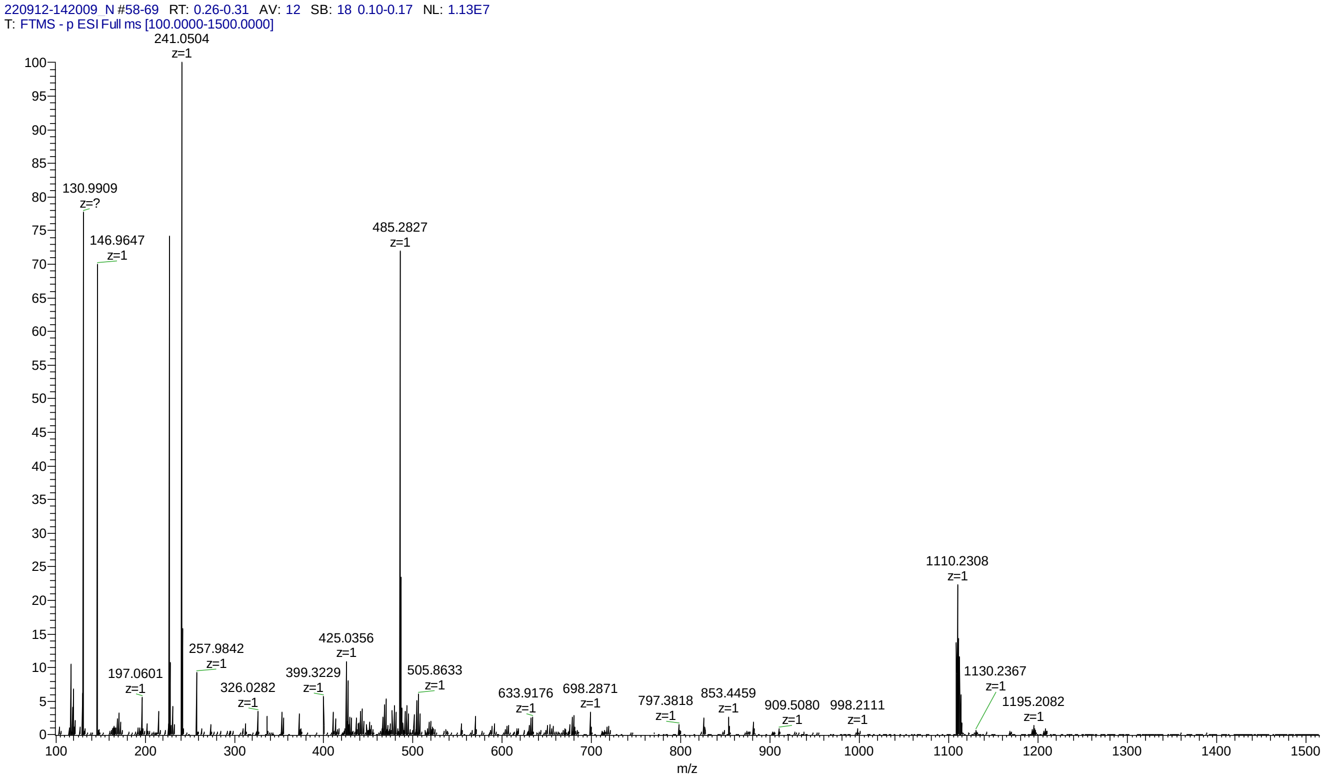

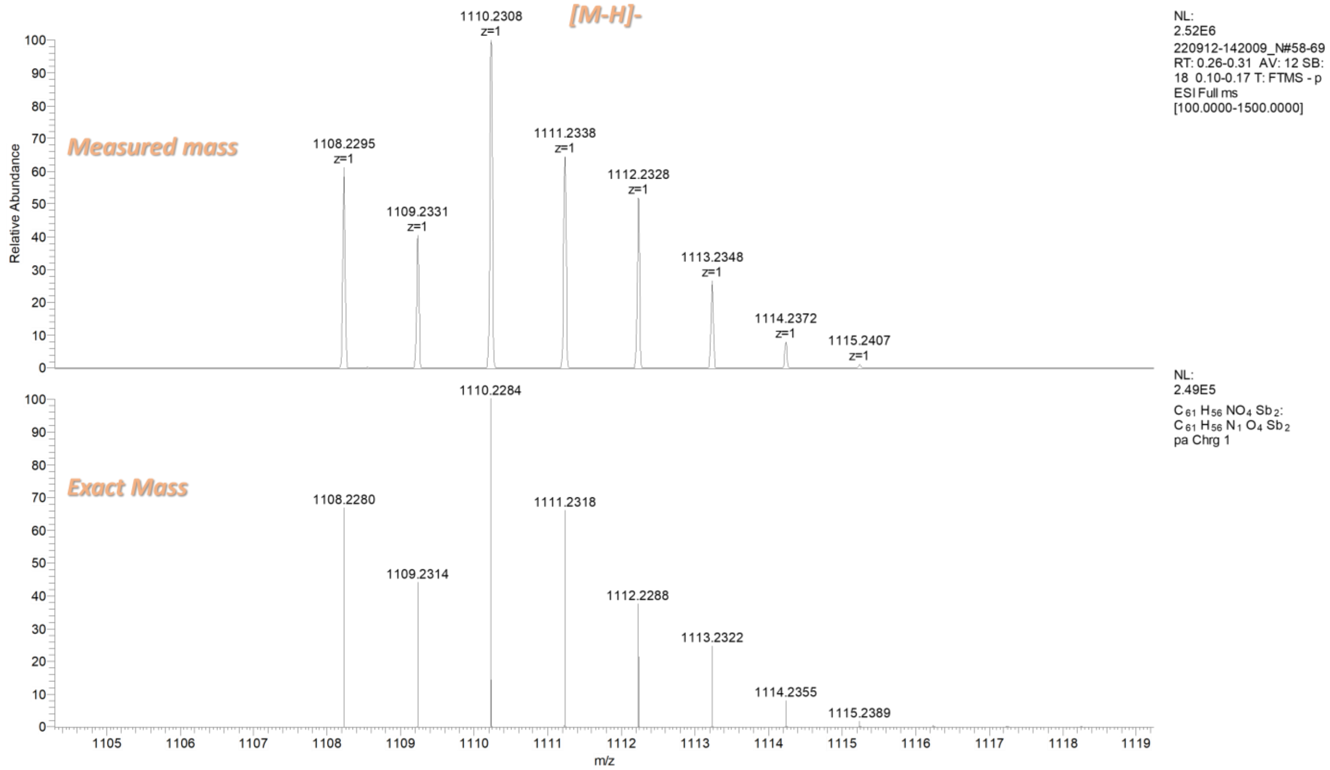

Figure S23. Top: ESI$^-$ mass spectrum of compound $\mathbf{3_{NH}}$. Bottom: the deprotonated anion was detected at a monoisotopic mass of 1108.2295 corresponding to $[C_{61}H_{56}NO_4Sb_2]^-$.

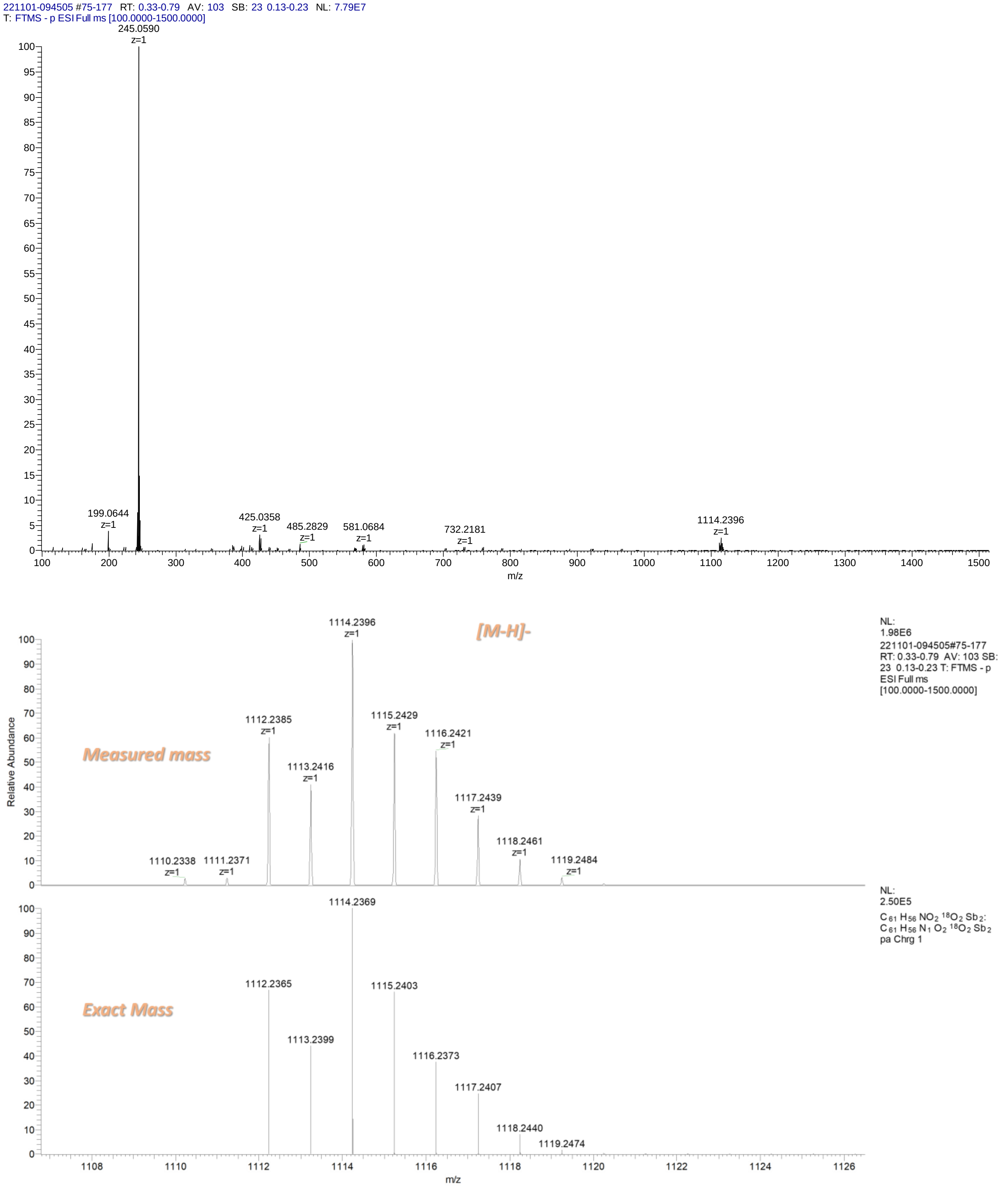

Figure S24. Top: ESI^- mass spectrometry of the reaction mixture of $\mathbf{1_{NH}}$ and 9,10-phenanthraquinone under an $^{18}O_2$ atmosphere overnight. Bottom: the deprotonated anion was detected at a monoisotopic mass of 1112.2385 corresponding to $[C_{61}H_{56}N^{16}O_2{}^{18}O_2Sb_2]^-$.

6 Reactivities of compounds **3**$_O$ and **5**$_O$.

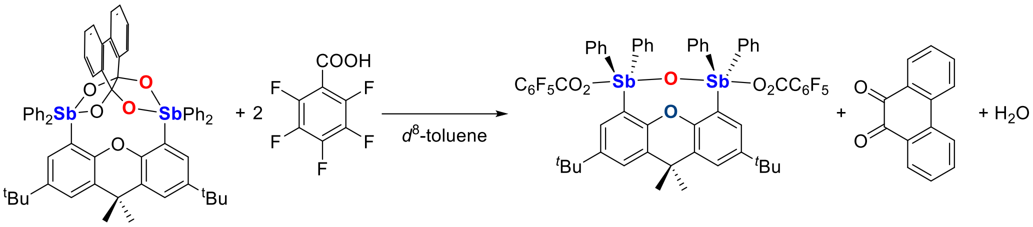

3$_O$ **5**$_O$

Two solutions were prepared as follows:

1. Compound **3**$_O$ (30.0 mg, 0.0270 mmol) and pentafluorobenzoic acid (11.4 mg, 0.0538 mmol, 2 equiv.) in d^8-toluene (0.7 mL).
2. Compound **3**$_O$ (30.0 mg, 0.0270 mmol) in d^8-toluene (0.7 mL).

An instant color change from light yellow into orange was seen upon mixing **3**$_O$ with C_6F_5COOH, the ^{1}H NMR spectra of both solutions were recorded immediately (see **Figure S25**). The spectrum collected in the presence of pentafluorobenzoic acid showed **5**$_O$ as a the only antimony-containing product.

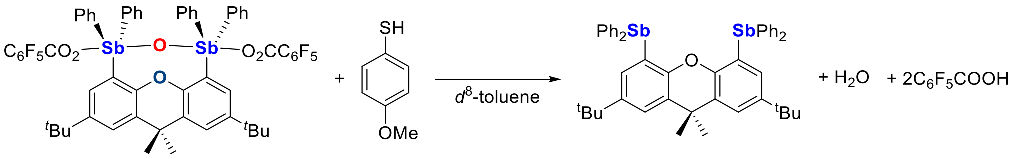

5$_O$ **1**$_O$

Two solutions were prepared as follows:

1. Compound **5**$_O$ (30.0 mg, 0.0229 mmol) and 4-methoxybenzenethiol (14.0 µL, 16.0 mg, 0.114 mmol, 5 equiv.) in d^8-toluene (0.7 mL).
2. Compound **5**$_O$ (30.0 mg, 0.0229 mmol) in d^8-toluene (0.7 mL).

The ^{1}H NMR spectra of both solutions were recorded 30 min after preparation (**Figure S26**).

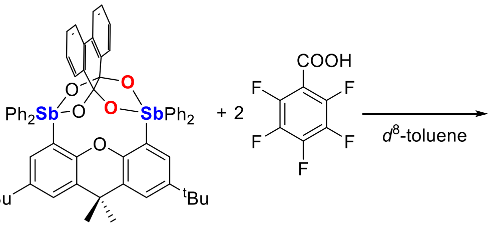

3$_{NH}$

The acidiolysis of **3**$_{NH}$ was also tested by preparing two solutions:

1. Compound **3**$_{NH}$ (30 mg, 0.0270 mmol) and pentafluorobenzoic acid (11.4 mg, 0.0538 mmol, 2 equiv.) in d^8-toluene (0.7 mL).
2. Compound **3**$_{NH}$ (30 mg, 0.0270 mmol) in d^8-toluene (0.7 mL).

The 1H NMR spectra of both solutions were recorded immediately (**Figure S27**). The results showed that the reaction between **3**$_{NH}$ and 2 equiv. of C_6F_5COOH was in complete and not clean, showing at least two products and some **3**$_{NH}$ leftover.

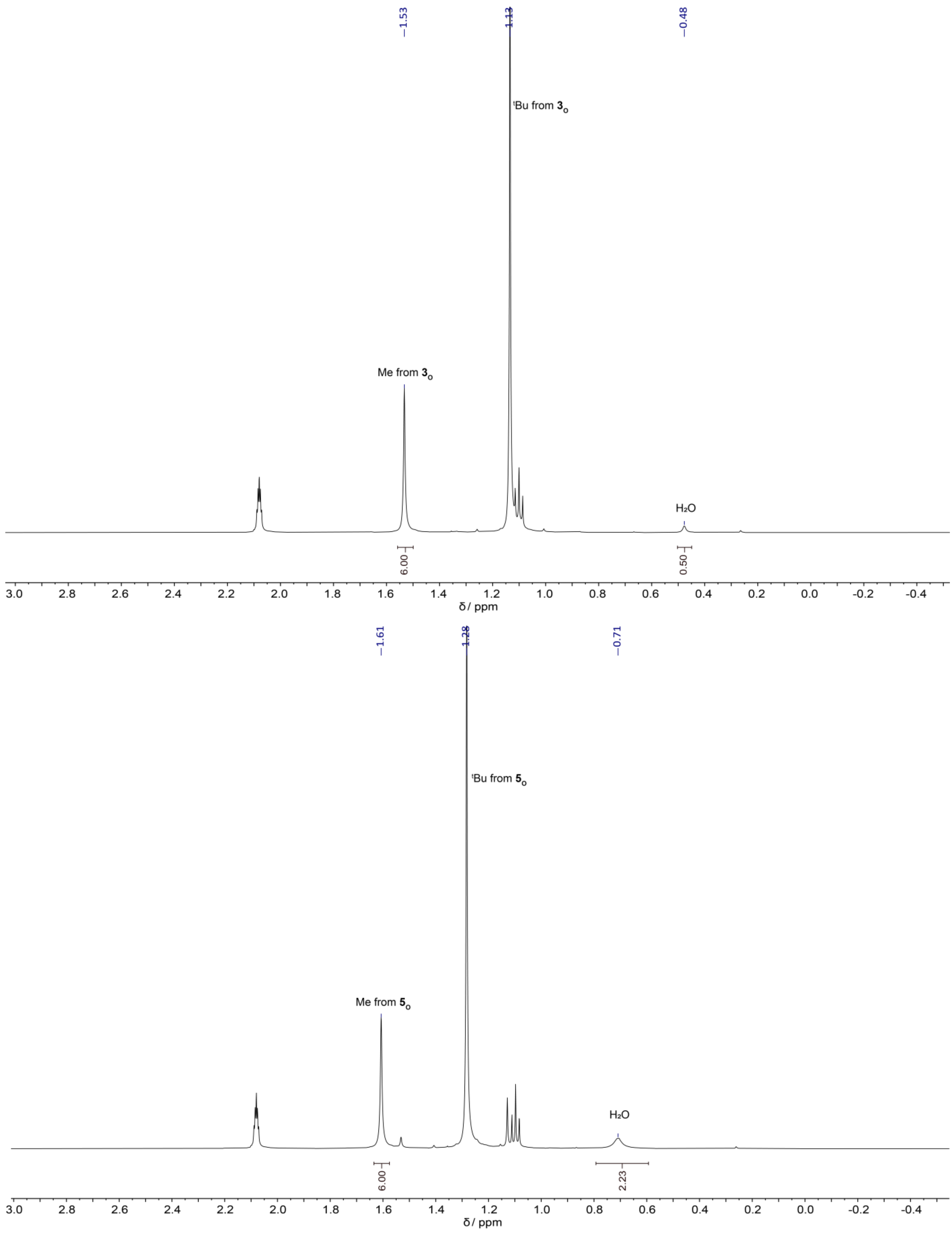

Figure S25. Portions of the ^{1}H NMR (d^8-toluene, 500.1 MHz) spectra of a solution of **3o** (top) and a mixture of **3**$_O$ and C_6F_5COOH (bottom).

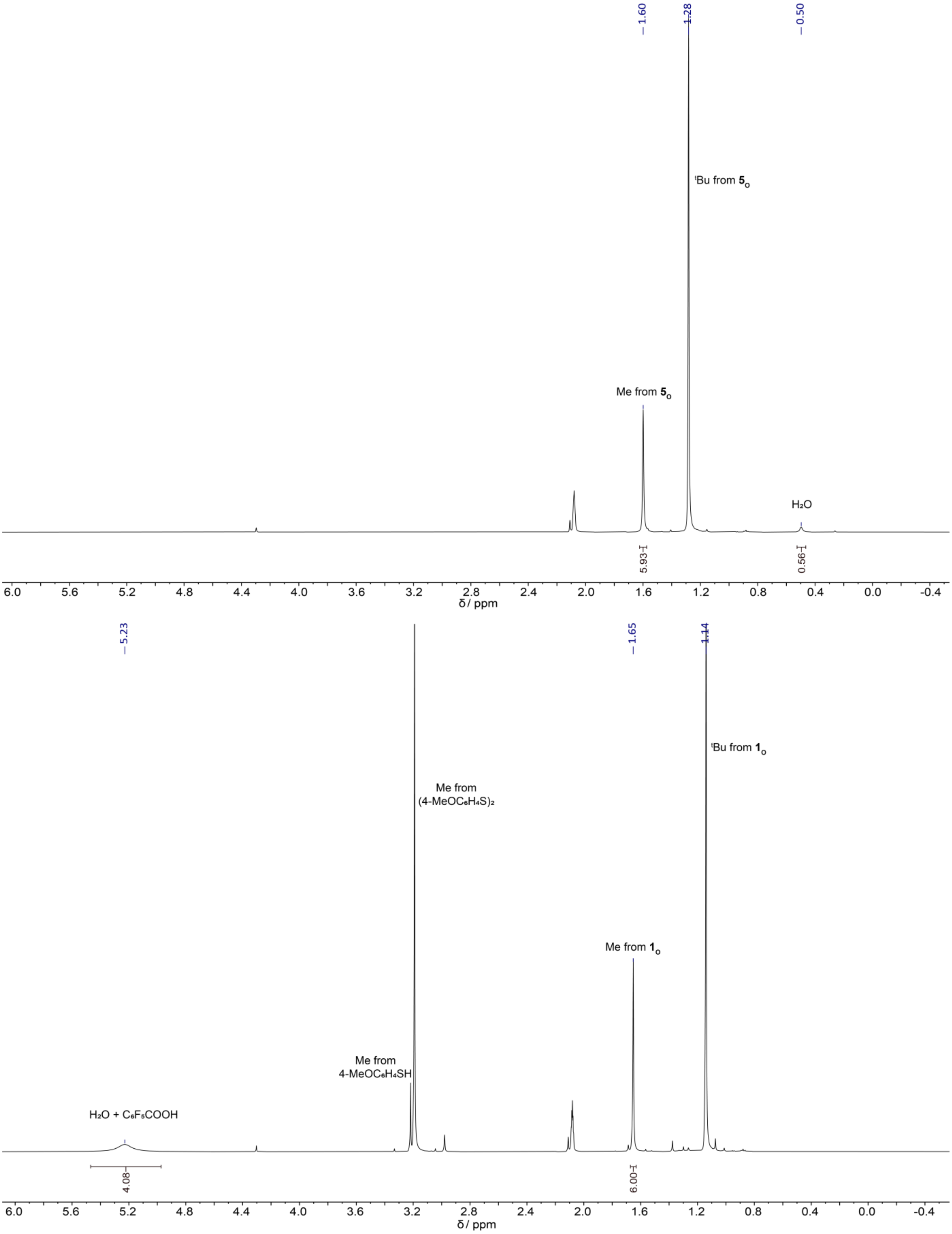

Figure S26. Portions of the ^{1}H NMR (d^8-toluene, 500.1 MHz) spectra of a solution of $\mathbf{5_O}$ (top) and a mixture of $\mathbf{5_O}$ and 4-methoxybenzenethiol (bottom) 30 min after mixing.

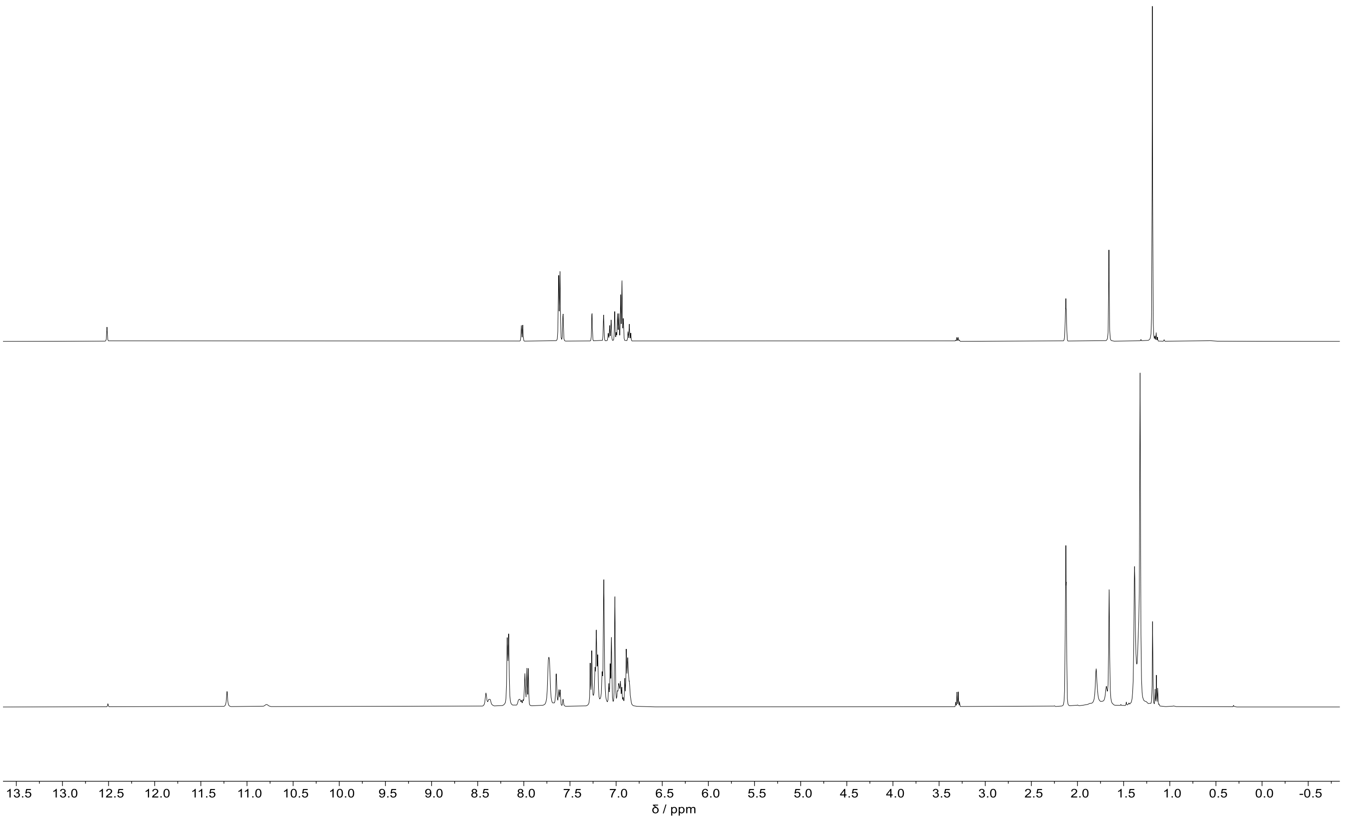

Figure S27. ^{1}H NMR (d^8-toluene, 500.1 MHz) spectra of a solution of $\mathbf{3_{NH}}$ (top) and a mixture of $\mathbf{3_{NH}}$ and C_6F_5COOH (bottom).

7 Coordinates and energies of all the computed local minima and the transition states.

7.1 9,10-phenanthraquinone

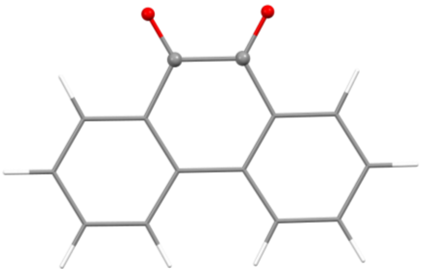

C	-4.712774	-4.553148	-0.004026
C	-3.324434	-4.562048	-0.005023
C	-2.607618	-3.361379	-0.003609
C	-3.270972	-2.116974	-0.001149
C	-4.673216	-2.131496	-0.000259
C	-5.382705	-3.328696	-0.001664
C	-1.121467	-3.451552	-0.004910
C	-2.499732	-0.844357	0.000457
C	-1.089612	-0.856528	-0.001034
C	-0.321920	-2.132235	-0.004023
C	-0.356968	0.334548	0.000332
H	0.731360	0.260477	-0.000964
C	-1.007343	1.561159	0.003273
C	-2.402818	1.588394	0.004869
C	-3.135778	0.405417	0.003491
H	-5.272120	-5.489161	-0.005106
H	-2.755123	-5.492547	-0.006897
H	-5.234490	-1.198888	0.001517
H	-6.473403	-3.302639	-0.000913
H	-0.436437	2.490166	0.004356
H	-2.930662	2.543211	0.007236
H	-4.222257	0.471388	0.004867
O	0.880342	-2.175660	-0.005744
O	-0.526617	-4.497201	-0.006680

7.2 O_2

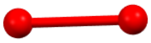

O	-3.018366	-0.140449	0.000000
O	-4.201286	-0.140449	0.000000

7.3 4,5-bis(diphenylstibino)-9,9-dimethylxanthene, **XanSb$_2$**

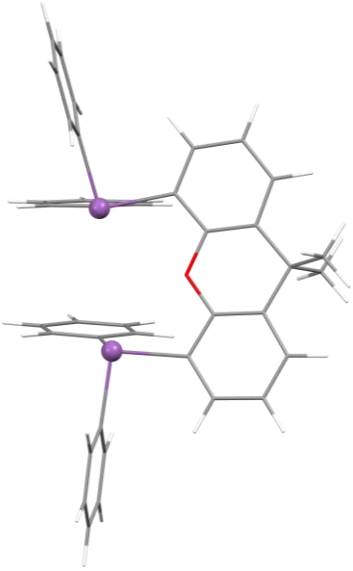

C	6.543455	-0.788523	-2.573457
C	6.333896	-1.049915	-1.219677
C	5.042334	-1.019623	-0.690032
C	3.942731	-0.729138	-1.508158
C	4.166998	-0.476569	-2.868305
C	5.457263	-0.500564	-3.399106
Sb	1.906005	-0.624201	-0.791871
C	2.043466	1.454936	-0.205004
C	3.262130	2.103300	-0.011416
C	3.299991	3.426421	0.433956
C	2.111812	4.098800	0.691974
C	0.863574	3.491035	0.504316
C	0.863181	2.170458	0.043983
O	-0.300580	1.486328	-0.184886
C	-1.516212	2.086390	0.007120
C	-1.636194	3.389219	0.492971
C	-0.421778	4.252942	0.816639
C	-2.623859	1.286282	-0.307225
C	-3.898075	1.813834	-0.112138
C	-4.056478	3.112254	0.380985
C	-2.936694	3.881238	0.672399
Sb	-2.241413	-0.647359	-1.185167
C	-1.450797	-1.570775	0.589660
C	-1.297121	-0.900978	1.807866
C	-0.812294	-1.575447	2.931622
C	-0.483063	-2.926521	2.851242
C	-0.629473	-3.603495	1.639743
C	-1.101768	-2.926737	0.516184
C	-0.441390	4.615661	2.314015
C	-0.474166	5.538966	-0.029579
C	-4.262694	-1.347704	-0.900953
C	-5.126989	-1.365035	-2.003085
C	-6.457280	-1.766689	-1.863718
C	-6.935175	-2.164309	-0.616330
C	-6.081977	-2.158630	0.488226
C	-4.754869	-1.752333	0.347111
C	2.362803	-1.402091	1.163786
C	2.638370	-2.771040	1.277465
C	2.980267	-3.332646	2.507452
C	3.032424	-2.530604	3.647767
C	2.747490	-1.169340	3.549820
C	2.419463	-0.607388	2.314035
H	2.149266	5.129413	1.050916
H	-3.070813	4.895779	1.053367
H	4.195701	1.570744	-0.206895
H	-0.687999	-1.039396	3.874346
H	-4.763169	-1.061678	-2.989318
H	-7.119058	-1.772525	-2.731561
H	-0.405669	3.706745	2.931148
H	-1.355699	5.171315	2.568163
H	0.421950	5.245264	2.573871
H	-1.564748	0.155613	1.890996
H	2.593438	-3.415733	0.393816
H	-0.098144	-3.449463	3.728111
H	-4.777785	1.207650	-0.341627
H	-6.452523	-2.472571	1.465693
H	-7.973040	-2.482341	-0.504159
H	3.201797	-4.399287	2.576776
H	2.210462	0.463642	2.250021
H	-1.202702	-3.469328	-0.429073
H	3.324495	-0.256802	-3.531020
H	0.384600	6.188582	0.191954
H	-1.389608	6.108943	0.184017
H	-0.458685	5.296393	-1.101269
H	3.297538	-2.967016	4.612396
H	-4.095846	-1.751195	1.219494
H	-0.364034	-4.659996	1.568089
H	7.180883	-1.277546	-0.569889
H	5.613959	-0.298279	-4.460072
H	2.787368	-0.537335	4.439002
H	4.893985	-1.222921	0.373741
H	7.553490	-0.811909	-2.985904
H	-5.055245	3.521784	0.535916
H	4.256452	3.928280	0.583842

7.4 4,5-bis(diphenylstibino)-9,9-dimethyl-9,10-dihydroacridine, **AcrSb$_2$**

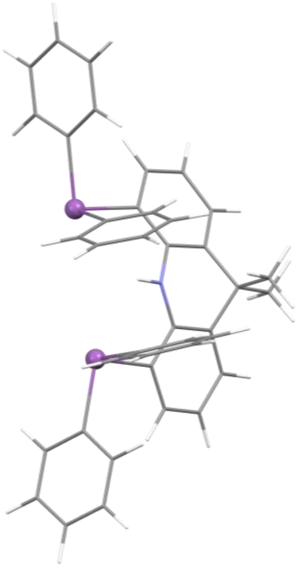

Sb	2.439638	-0.689298	1.216671
Sb	-2.127762	-0.643640	0.867901
C	2.799983	3.995778	-0.329259
H	2.883508	5.051897	-0.585321
C	3.931131	3.315784	0.120041
C	3.840114	1.965073	0.449723
H	4.730975	1.436929	0.799725
C	2.622123	1.283310	0.361608
C	-2.111198	1.465482	0.398374
C	-3.244608	2.262077	0.589094
H	-4.169582	1.809914	0.953255
C	-3.208136	3.632514	0.338992
C	-2.042679	4.215534	-0.156509
H	-2.036434	5.282940	-0.376939
C	0.342129	3.975941	-1.115863
C	1.577496	3.341305	-0.479533
C	1.496503	1.982504	-0.115050
C	-0.933318	2.080855	-0.067617
C	-0.901052	3.451919	-0.396539
C	0.410124	5.502006	-1.110363
H	1.287880	5.850828	-1.670583
H	0.463673	5.900952	-0.086766
H	-0.470630	5.928363	-1.609015
C	0.271264	3.490414	-2.582956
H	-0.629744	3.892076	-3.070552
H	0.233821	2.391984	-2.630452
H	1.160539	3.829770	-3.134993
C	4.495935	-1.236913	0.899824
C	5.356484	-1.241855	2.004748
H	4.972182	-1.002307	3.000524
C	6.709903	-1.551109	1.853903
H	7.369713	-1.548116	2.723139
C	7.213218	-1.868039	0.593685
H	8.269535	-2.113528	0.472628
C	6.362573	-1.874871	-0.512744
H	6.753288	-2.126802	-1.500138
C	5.012194	-1.560441	-0.361095
H	4.356190	-1.571218	-1.234919
C	1.620829	-1.634892	-0.542275
C	1.712166	-1.069771	-1.822428
H	2.205747	-0.102354	-1.958473
C	1.171910	-1.727172	-2.928633
H	1.240627	-1.271882	-3.918303
C	0.538789	-2.959436	-2.770169
H	0.107385	-3.468011	-3.633683
C	0.438439	-3.530111	-1.502078
H	-0.068668	-4.487912	-1.372862
C	0.969482	-2.867232	-0.395008
H	0.868376	-3.318083	0.596737
C	-4.207779	-0.754702	1.396136
C	-4.553154	-0.813724	2.751941
H	-3.771840	-0.828214	3.517060
C	-5.892237	-0.854775	3.144988
H	-6.147577	-0.898445	4.205057
C	-6.899809	-0.843999	2.181983
H	-7.947171	-0.878620	2.486223
C	-6.566678	-0.791175	0.827835
H	-7.353505	-0.782956	0.071588
C	-5.228522	-0.746461	0.436408
H	-4.978407	-0.701925	-0.626774
C	-2.392297	-1.310061	-1.165938
C	-2.150647	-0.488739	-2.272014
H	-1.823788	0.545002	-2.129408
C	-2.344233	-0.971293	-3.569405
H	-2.152802	-0.318524	-4.423132
C	-2.791952	-2.274881	-3.773248
H	-2.953712	-2.646652	-4.786619
C	-3.037654	-3.102798	-2.676309
H	-3.392721	-4.123721	-2.828872
C	-2.829359	-2.624224	-1.383920
H	-3.025392	-3.285980	-0.534105
N	0.251052	1.361248	-0.217481
H	0.221214	0.351122	-0.153042
H	4.883225	3.839506	0.212116
H	-4.093811	4.244121	0.514110

7.5 Intermediate **I1$_O$**.

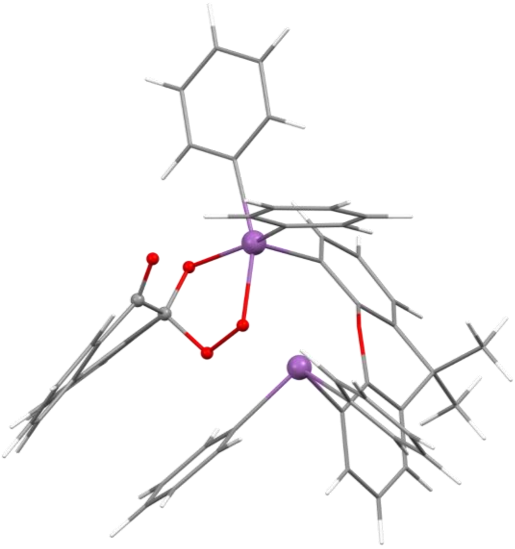

Sb	-1.898678	-0.945177	-0.793937
Sb	2.375127	0.013291	-0.567325
O	-1.836470	1.632062	-1.883545
O	-3.056545	0.443541	0.118852
O	-0.488622	0.268163	0.145738
O	-1.123483	1.122933	1.080761
O	0.535291	-2.243276	0.898568
C	-1.873892	-3.959780	3.036494
H	-1.909317	-4.538716	3.959625
C	-3.054744	-3.736689	2.328401
C	-3.030886	-2.970748	1.165735
H	-3.960312	-2.763311	0.628654
C	-1.826211	-2.437177	0.696770
C	2.322193	-0.765289	1.453408
C	3.187527	-0.276239	2.440044
H	3.843523	0.566215	2.203657
C	3.216268	-0.833342	3.716949
C	2.402517	-1.925124	4.019583
H	2.458286	-2.375671	5.010664
C	0.693321	-3.711291	3.253102
C	-0.651382	-3.463759	2.580766
C	-0.646497	-2.718357	1.394444
C	1.472727	-1.811093	1.816670
C	1.525672	-2.444355	3.066993
C	0.539609	-4.093372	4.723550
H	-0.033958	-5.024986	4.823230
H	0.032618	-3.302538	5.295336
H	1.523509	-4.278601	5.175146
C	1.401739	-4.862148	2.503523
H	2.398509	-5.034976	2.935226
H	1.524543	-4.618418	1.438577
H	0.810833	-5.786811	2.583416
C	-3.631826	-1.513926	-1.910578
C	-4.881091	-0.903880	-1.750511
H	-4.985316	-0.065642	-1.061971
C	-5.980559	-1.366677	-2.475145
H	-6.951586	-0.884331	-2.351402
C	-5.841903	-2.439413	-3.355523
H	-6.704200	-2.798003	-3.920044
C	-4.599203	-3.050731	-3.517996
H	-4.484894	-3.887096	-4.209280
C	-3.496823	-2.586158	-2.800172
H	-2.523347	-3.062528	-2.948303
C	-0.438879	-1.385131	-2.274629
C	0.279691	-2.578726	-2.154032
H	0.086543	-3.264312	-1.326180
C	1.284876	-2.884484	-3.072017
H	1.855559	-3.808106	-2.964552
C	1.568370	-2.002130	-4.113757
H	2.366360	-2.232994	-4.821221
C	0.824869	-0.830149	-4.255950
H	1.035080	-0.146332	-5.079999
C	-0.183090	-0.520952	-3.342745
H	-0.747122	0.405766	-3.449217
C	2.640842	2.060738	0.077217
C	1.762536	2.604346	1.026018
H	0.982184	1.983416	1.471952
C	1.875776	3.939752	1.413650
H	1.188904	4.344332	2.159932
C	2.857909	4.754510	0.849154
H	2.945189	5.799730	1.152946
C	3.729516	4.226736	-0.101924
H	4.506187	4.855085	-0.542339
C	3.622227	2.886759	-0.482968
H	4.330079	2.483194	-1.212221
C	4.493077	-0.361797	-0.907714
C	5.393682	-0.955139	-0.013773
H	5.061384	-1.254090	0.982654
C	6.723009	-1.179648	-0.378210
H	7.408012	-1.644837	0.333338
C	7.178109	-0.812339	-1.643928
H	8.218256	-0.985255	-1.925271
C	6.293007	-0.231911	-2.551546
H	6.636581	0.049682	-3.548763
C	4.962652	-0.022051	-2.185998
H	4.275536	0.411873	-2.921264
C	-3.946593	1.964995	2.300789
H	-4.174689	0.899708	2.253874
C	-4.565866	2.802895	3.225079
H	-5.296832	2.399666	3.926894
C	-4.250770	4.160713	3.239828
H	-4.738951	4.828858	3.950712
C	-3.319721	4.676151	2.342003
H	-3.108818	5.745200	2.356699
C	-1.106184	5.649271	0.583570
H	-1.354504	6.271307	1.443138
C	-0.188646	6.124913	-0.349730
H	0.258126	7.109996	-0.204772
C	0.179580	5.351587	-1.452171
H	0.916219	5.722413	-2.165101
C	-0.388533	4.094946	-1.616439
H	-0.120848	3.452078	-2.456371
C	-1.852497	2.256023	-0.841721
C	-2.328152	1.545990	0.443608
C	-3.016871	2.482171	1.403032
C	-2.676325	3.846958	1.413665
C	-1.682508	4.380652	0.444093
C	-1.304568	3.613985	-0.676929
H	-3.996351	-4.145344	2.695944
H	3.886243	-0.427351	4.475879

7.6 Transition state **TS1$_O$**.

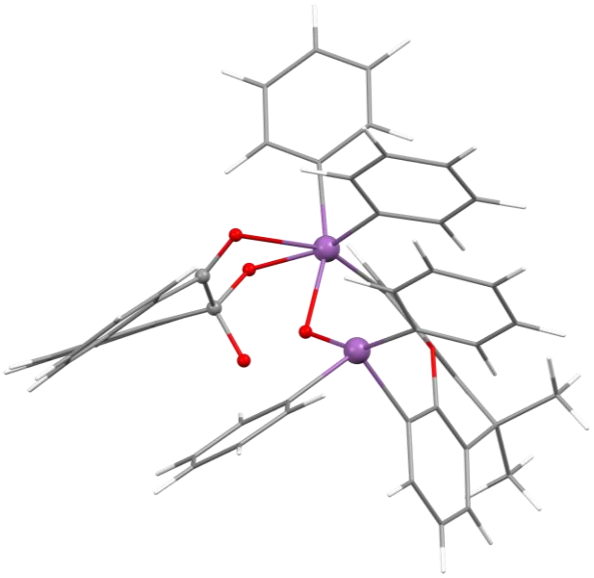

Sb	-0.651652	-1.608874	-0.736395
Sb	1.427407	1.400321	-0.062357
O	-1.991598	0.319329	-1.722879
O	-2.460815	-1.464078	0.094368
O	-0.338249	0.237615	0.395712
O	-1.829915	0.250279	1.422867
O	2.044134	-1.326533	1.050206
C	1.084872	-4.213791	3.044065
H	1.383518	-4.753768	3.943120
C	-0.009712	-4.670955	2.307315
C	-0.431371	-3.982125	1.171450
H	-1.310534	-4.323653	0.618925
C	0.247182	-2.833017	0.753181
C	2.441827	0.886644	1.749352
C	2.909116	1.770217	2.725100
H	2.853335	2.848587	2.554910
C	3.409941	1.276903	3.931045
C	3.445911	-0.098367	4.176585
H	3.828299	-0.457900	5.132499
C	3.048815	-2.534069	3.334633
C	1.799658	-3.084204	2.642735
C	1.361581	-2.430263	1.485734
C	2.512626	-0.475928	2.022621
C	2.999660	-1.010206	3.217644
C	3.144978	-2.991199	4.788518
H	3.209617	-4.086346	4.845803
H	2.275453	-2.657321	5.372950
H	4.056937	-2.594867	5.255619
C	4.287343	-3.034605	2.558701
H	5.204777	-2.624716	3.006305
H	4.243542	-2.718046	1.506684
H	4.333906	-4.133226	2.588782
C	-1.508577	-3.134716	-1.992432
C	-2.885479	-3.369698	-2.079265
H	-3.571469	-2.750531	-1.501249
C	-3.374643	-4.391203	-2.896077
H	-4.450530	-4.562159	-2.962821
C	-2.495146	-5.192911	-3.622287
H	-2.880274	-5.992595	-4.257306
C	-1.121141	-4.968749	-3.537580
H	-0.427429	-5.591681	-4.104646
C	-0.633249	-3.939967	-2.731777
H	0.445891	-3.764837	-2.690811
C	0.841080	-1.092049	-2.190683
C	2.154153	-1.545040	-2.023456
H	2.434408	-2.127013	-1.143694
C	3.132296	-1.226010	-2.968670
H	4.158610	-1.565631	-2.819505
C	2.801332	-0.470897	-4.091708
H	3.569402	-0.207963	-4.820513
C	1.482513	-0.054901	-4.279752
H	1.213778	0.522462	-5.166349
C	0.500702	-0.368915	-3.339462
H	-0.526030	-0.035904	-3.496129
C	0.581617	3.271316	0.499957
C	-0.567186	3.248814	1.306388
H	-0.997718	2.294441	1.624876
C	-1.174359	4.446672	1.684067
H	-2.066035	4.422301	2.313310
C	-0.654954	5.666859	1.249571
H	-1.137059	6.601729	1.541833
C	0.482673	5.692488	0.443156
H	0.894773	6.645496	0.106540
C	1.103987	4.497875	0.072676
H	2.003242	4.528150	-0.547948
C	3.127681	2.032250	-1.254710
C	4.426797	1.612912	-0.946328
H	4.624151	1.087307	-0.007581
C	5.477183	1.861372	-1.832017
H	6.487919	1.533230	-1.582506
C	5.237130	2.530517	-3.032345
H	6.059311	2.725576	-3.722931
C	3.944586	2.951019	-3.348267
H	3.753162	3.473091	-4.287649
C	2.894251	2.690564	-2.468421
H	1.879379	2.999012	-2.740680
C	-4.675652	-1.109999	1.801343
H	-4.124561	-2.050849	1.823876
C	-5.905952	-0.969233	2.439090
H	-6.343835	-1.812474	2.975103
C	-6.578609	0.251117	2.381838
H	-7.547892	0.367014	2.868712
C	-6.022037	1.324769	1.691699
H	-6.576661	2.261716	1.634039
C	-4.620274	3.657416	0.432918
H	-5.390641	3.901647	1.164245
C	-4.067319	4.679800	-0.335696
H	-4.416948	5.703611	-0.191515
C	-3.069862	4.414219	-1.276475
H	-2.634765	5.223239	-1.863653
C	-2.622390	3.108695	-1.434216
H	-1.829343	2.857375	-2.141081
C	-2.616784	0.727008	-0.754537
C	-2.737181	-0.175403	0.499654
C	-4.120824	-0.036983	1.108795
C	-4.781480	1.204639	1.051999
C	-4.184923	2.333696	0.290283
C	-3.163809	2.086051	-0.650320
H	-0.547981	-5.560989	2.634438
H	3.762860	1.969197	4.696341

7.7 Intermediate **I2$_O$**.

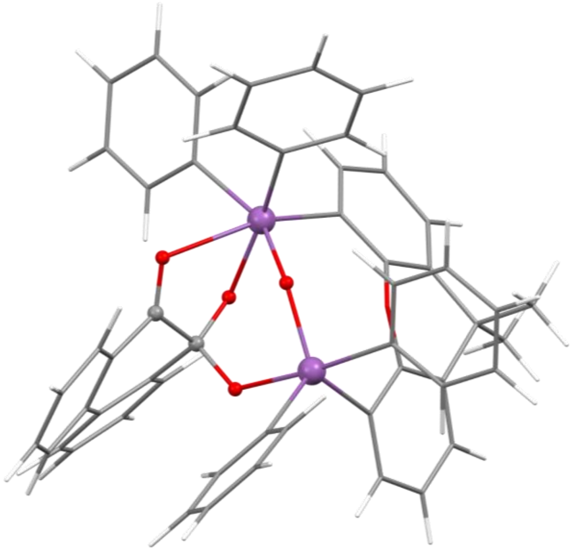

Sb	0.963350	1.557010	0.640774
Sb	-0.570316	-1.723779	0.129969
O	-1.416198	1.983641	1.203976
O	-0.124449	1.436328	-1.063764
O	0.115781	-0.196386	1.087762
O	-1.563889	-0.370702	-1.061384
O	2.046612	-1.130484	-0.776902
C	4.748805	0.680212	-2.400690
H	5.518030	0.543766	-3.161398
C	4.652844	1.904483	-1.739627
C	3.648826	2.120134	-0.794452
H	3.550720	3.105536	-0.333486
C	2.742739	1.104078	-0.474440
C	0.342150	-2.457838	-1.651908
C	-0.141489	-3.302724	-2.648379
H	-1.158384	-3.697944	-2.597141
C	0.681609	-3.624301	-3.732198
C	1.983486	-3.123382	-3.819136
H	2.601776	-3.397521	-4.674732
C	3.932562	-1.764377	-2.723183
C	3.875085	-0.366781	-2.098925
C	2.910023	-0.124217	-1.115291
C	1.639891	-1.974912	-1.775251
C	2.500046	-2.287676	-2.824622
C	4.632461	-1.757185	-4.080542
H	5.674551	-1.425251	-3.978057
H	4.119595	-1.096021	-4.794054
H	4.663262	-2.772168	-4.499971
C	4.700031	-2.690353	-1.752772
H	4.733143	-3.714046	-2.154180
H	4.208159	-2.720630	-0.769514
H	5.728638	-2.324832	-1.616670
C	0.980509	3.701710	0.465238
C	0.239003	4.364539	-0.519427
H	-0.384720	3.789472	-1.205862
C	0.300615	5.753680	-0.631253
H	-0.286345	6.260597	-1.399113
C	1.111125	6.493477	0.230294
H	1.158998	7.579917	0.138946
C	1.858812	5.840744	1.208462
H	2.495017	6.412804	1.885957
C	1.789491	4.450858	1.327270
H	2.373704	3.953405	2.106075
C	1.589329	1.500325	2.688943
C	2.708889	0.738618	3.035364
H	3.280761	0.216914	2.261710
C	3.099267	0.628606	4.371944
H	3.976249	0.034514	4.635241
C	2.367436	1.276351	5.366587
H	2.670591	1.190363	6.411401
C	1.247428	2.035986	5.025120
H	0.673647	2.542735	5.803009
C	0.859158	2.148981	3.689619
H	-0.017333	2.747689	3.427359
C	-2.487943	-2.489010	0.645705
C	-3.621998	-2.184814	-0.121502
H	-3.520877	-1.555388	-1.006169
C	-4.871075	-2.671996	0.260664
H	-5.749004	-2.435110	-0.342917
C	-5.001202	-3.446274	1.414388
H	-5.982195	-3.818527	1.715034
C	-3.877629	-3.745780	2.182623
H	-3.975146	-4.349554	3.086171
C	-2.621234	-3.275854	1.795672
H	-1.744343	-3.522787	2.398110
C	0.588539	-3.008167	1.426932
C	0.821259	-4.335734	1.051081
H	0.429299	-4.716575	0.102107
C	1.564302	-5.187858	1.870274
H	1.742531	-6.220431	1.564810
C	2.081607	-4.714955	3.076419
H	2.664893	-5.378122	3.717701
C	1.854363	-3.392658	3.458840
H	2.260158	-3.017050	4.400336
C	1.111266	-2.541394	2.638631
H	0.943103	-1.502708	2.931170
C	-1.615820	1.968403	-3.338773
H	-0.527091	1.918170	-3.381262
C	-2.370624	2.410349	-4.425525
H	-1.872862	2.718206	-5.346261
C	-3.759722	2.465552	-4.328192
H	-4.355751	2.821426	-5.169724
C	-4.392595	2.082496	-3.147329
H	-5.477703	2.164508	-3.072641
C	-5.656281	0.926892	-0.665420
H	-6.278963	0.899183	-1.560143
C	-6.222797	0.619096	0.571672
H	-7.280879	0.356562	0.624431
C	-5.454515	0.637180	1.736222
H	-5.904544	0.388296	2.697567
C	-4.103717	0.962777	1.655305
H	-3.465214	0.974785	2.540350
C	-2.083562	1.496837	0.307673
C	-1.423790	1.007435	-1.006706
C	-2.247569	1.578456	-2.162235
C	-3.649674	1.627969	-2.051726
C	-4.299431	1.249362	-0.769534
C	-3.534562	1.249118	0.414174
H	0.304195	-4.274898	-4.521965
H	5.348400	2.707934	-1.984055

7.8 Transition state **TS2$_O$**.

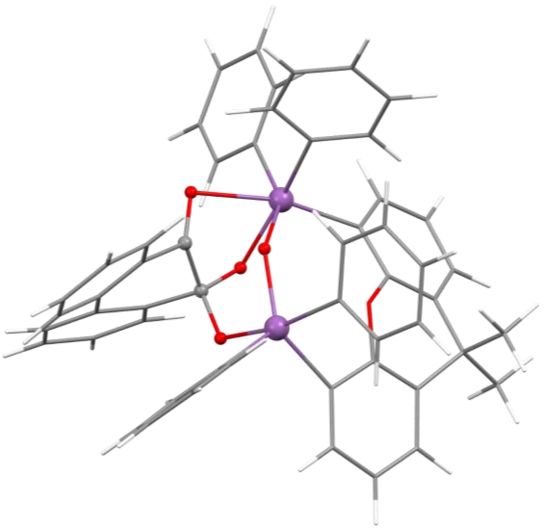

Sb	1.551951	1.242423	0.441046	H	1.626213	3.692946	-1.526080	C	-0.616227	-4.909371	2.265685
Sb	-1.309305	-1.215936	-0.066810	C	3.133930	5.118996	-0.916058	H	-0.746509	-5.977138	2.080992
O	-0.194272	2.576981	0.977236	H	2.927927	5.804242	-1.740068	C	-0.112612	-4.466079	3.489109
O	0.542146	1.486096	-1.292002	C	4.122413	5.429001	0.018857	H	0.151465	-5.187560	4.264305
O	-0.285842	0.164054	0.871953	H	4.691762	6.355291	-0.074859	C	0.056071	-3.100671	3.717039
O	-1.454946	0.323898	-1.407251	C	4.377436	4.557925	1.076927	H	0.456856	-2.746978	4.668957
O	1.368870	-1.790327	-0.716529	H	5.144145	4.800838	1.814546	C	-0.278316	-2.175288	2.725467
C	4.720466	-1.581756	-2.122945	C	3.644892	3.374896	1.197405	H	-0.128513	-1.107779	2.901660
H	5.409808	-2.147409	-2.750178	H	3.843611	2.702269	2.037048	C	-0.934981	3.070378	-3.119313
C	5.117570	-0.346096	-1.615647	C	1.929546	0.796064	2.494085	H	0.045223	2.684885	-3.404172
C	4.243595	0.408666	-0.831907	C	2.781452	-0.262968	2.816401	C	-1.610318	4.005019	-3.904814
H	4.558182	1.392377	-0.477935	H	3.293390	-0.820449	2.027241	H	-1.157361	4.373708	-4.826345
C	2.962409	-0.067446	-0.534904	C	2.973209	-0.630187	4.151140	C	-2.861129	4.471198	-3.504118
C	-0.649349	-2.484943	-1.638443	H	3.635173	-1.462875	4.394802	H	-3.389062	5.211696	-4.106698
C	-1.369561	-3.235953	-2.563420	C	2.313534	0.061000	5.165205	C	-3.436801	4.005943	-2.322958
H	-2.460659	-3.189866	-2.587112	H	2.460472	-0.225916	6.207911	H	-4.400766	4.405214	-2.004330
C	-0.676719	-4.039780	-3.474330	C	1.464544	1.123289	4.845855	C	-4.723657	2.635646	0.048279
C	0.718040	-4.115694	-3.445079	H	0.948662	1.666921	5.639282	H	-5.419917	3.042975	-0.686011
H	1.228797	-4.761645	-4.160065	C	1.270146	1.491451	3.514802	C	-5.213522	2.152969	1.261837
C	2.967330	-3.483447	-2.276294	H	0.596280	2.313879	3.266214	H	-6.285612	2.193123	1.460907
C	3.457086	-2.102705	-1.834157	C	-3.420497	-1.297990	0.123106	C	-4.351719	1.609850	2.214185
C	2.617679	-1.323287	-1.029797	C	-4.235488	-0.657293	-0.818116	H	-4.743932	1.226046	3.156534
C	0.735612	-2.575030	-1.645976	H	-3.776652	-0.079001	-1.622511	C	-2.984852	1.550969	1.946307
C	1.462168	-3.388879	-2.510010	C	-5.623350	-0.745989	-0.706874	H	-2.277341	1.122081	2.659897
C	3.705950	-3.987112	-3.514758	H	-6.256845	-0.246354	-1.442006	C	-1.071615	1.934297	0.359752
H	4.779423	-4.096278	-3.309458	C	-6.200321	-1.457476	0.344772	C	-0.824909	1.520853	-1.112849
H	3.576361	-3.303147	-4.365950	H	-7.286604	-1.519121	0.430902	C	-1.511717	2.601984	-1.944507
H	3.337091	-4.981291	-3.801758	C	-5.389933	-2.089512	1.287614	C	-2.777496	3.061529	-1.528687
C	3.194715	-4.466317	-1.105333	H	-5.838597	-2.644376	2.113100	C	-3.359950	2.564501	-0.251206
H	2.824544	-5.466753	-1.374390	C	-4.001307	-2.016639	1.174723	C	-2.507461	2.007032	0.722234
H	2.659899	-4.131691	-0.204039	H	-3.371175	-2.518102	1.912818	H	-1.229790	-4.619844	-4.213917
H	4.267374	-4.531753	-0.870079	C	-0.783891	-2.611863	1.495121	H	6.109609	0.042438	-1.847879
C	2.660210	3.055790	0.256947	C	-0.948821	-3.984667	1.274471				
C	2.404427	3.935884	-0.799776	H	-1.332969	-4.346245	0.315160				

7.9 Product **P**$_{\mathbf{O}}$.

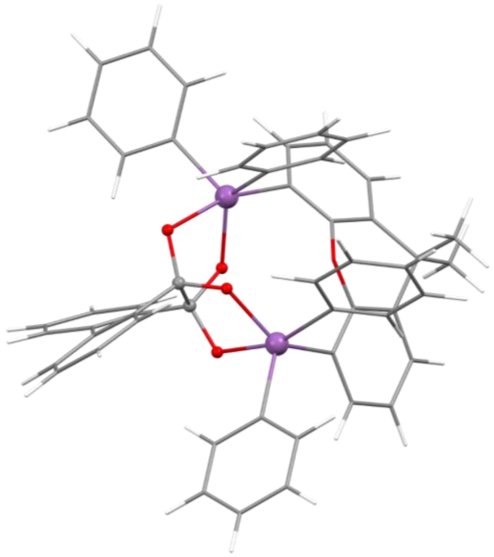

Sb	1.969917	0.591713	0.529111
Sb	-1.929718	-0.589989	-0.186606
O	0.729389	2.144794	1.006543
O	0.835888	0.689575	-1.105504
O	-1.006755	0.699414	1.025018
O	-1.392976	0.857048	-1.509014
O	0.759978	-2.123897	-0.602046
C	3.948629	-3.165391	-2.053124
H	4.371509	-3.968108	-2.657360
C	4.768595	-2.120284	-1.635365
C	4.241289	-1.082605	-0.864839
H	4.891189	-0.258166	-0.563760
C	2.890650	-1.071109	-0.509687
C	-1.383383	-2.075051	-1.609948
C	-2.280472	-2.567765	-2.557540
H	-3.283353	-2.140145	-2.635912
C	-1.893347	-3.600112	-3.413624
C	-0.623586	-4.164914	-3.298690
H	-0.343210	-4.983868	-3.961461
C	1.640420	-4.337971	-2.042022
C	2.595738	-3.197488	-1.705019
C	2.096623	-2.134825	-0.943892
C	-0.112487	-2.638915	-1.531468
C	0.285588	-3.706748	-2.341408
C	2.146767	-5.198870	-3.196568
H	3.108038	-5.663267	-2.938062
H	2.276017	-4.607396	-4.114602
H	1.444364	-6.018360	-3.401126
C	1.481637	-5.218004	-0.780899
H	0.765409	-6.030342	-0.975133
H	1.109480	-4.624221	0.067431
H	2.451630	-5.655538	-0.501703
C	3.612472	1.905989	0.626452
C	3.543547	3.131023	-0.045151

H	2.639948	3.406111	-0.595837
C	4.627358	4.007463	0.006696
H	4.575879	4.962070	-0.518831
C	5.768105	3.669856	0.735560
H	6.611294	4.361171	0.777792
C	5.831221	2.454855	1.417602
H	6.718881	2.196121	1.996745
C	4.753751	1.570076	1.361557
H	4.806710	0.619289	1.900391
C	1.624990	-0.365307	2.388984
C	1.899050	-1.724680	2.560664
H	2.260400	-2.327378	1.724684
C	1.708147	-2.322124	3.808062
H	1.908368	-3.386988	3.935590
C	1.263450	-1.558142	4.885078
H	1.122221	-2.024836	5.861778
C	0.988170	-0.200201	4.714281
H	0.635034	0.396718	5.557056
C	1.156319	0.397190	3.466195
H	0.916634	1.450920	3.314249
C	-3.979108	-0.141926	-0.349691
C	-4.386255	1.036881	-0.985966
H	-3.637946	1.704255	-1.418121
C	-5.744403	1.347173	-1.054134
H	-6.063154	2.270311	-1.540345
C	-6.690928	0.483036	-0.502954
H	-7.752414	0.729438	-0.562000
C	-6.284993	-0.692490	0.128836
H	-7.025112	-1.365252	0.564538
C	-4.927737	-1.003017	0.213624
H	-4.609976	-1.912295	0.731248
C	-1.896272	-1.941435	1.489110
C	-1.962727	-3.324240	1.280372
H	-1.988837	-3.730704	0.265836

C	-1.988376	-4.205576	2.363698
H	-2.036930	-5.281444	2.186652
C	-1.952819	-3.710361	3.665876
H	-1.973719	-4.397741	4.513429
C	-1.888377	-2.333814	3.882050
H	-1.850932	-1.940670	4.899529
C	-1.856873	-1.452431	2.801686
H	-1.781116	-0.378675	2.976467
C	0.822472	2.733501	-2.985433
H	1.282399	1.776643	-3.235508
C	1.083725	3.877167	-3.737088
H	1.749653	3.824826	-4.599722
C	0.500714	5.090230	-3.370832
H	0.714252	5.996535	-3.939710
C	-0.348106	5.150831	-2.269209
H	-0.774401	6.110914	-1.976023
C	-2.498614	5.058347	-0.182473
H	-2.594567	5.835429	-0.941723
C	-3.347906	5.075606	0.920444
H	-4.084836	5.873649	1.023379
C	-3.264420	4.068737	1.881334
H	-3.930620	4.074852	2.745155
C	-2.335603	3.042807	1.721475
H	-2.268225	2.230718	2.446679
C	-0.501024	1.877939	0.449341
C	-0.281046	1.538531	-1.063806
C	-0.026380	2.792160	-1.880872
C	-0.635608	4.003852	-1.516253
C	-1.546978	4.042217	-0.345586
C	-1.483015	3.022741	0.618839
H	-2.587625	-3.973553	-4.166998
H	5.821803	-2.108907	-1.918100

7.10 Intermediate **I1$_{NH}$**.

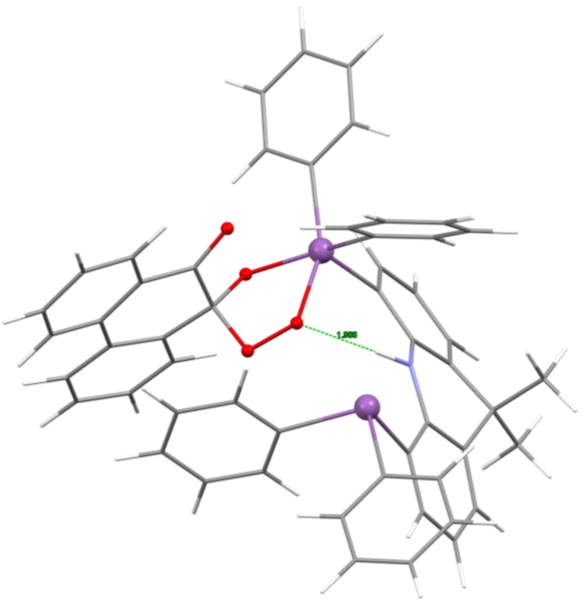

Sb	-1.849036	-1.305631	-0.532350
Sb	2.688279	0.630358	-0.900903
O	-2.775958	0.995705	-1.516202
O	-2.829792	-0.171568	0.837284
O	-0.510176	0.244677	-0.195126
O	-0.933918	1.088067	0.856920
C	-0.182642	-4.639677	2.642142
H	0.145011	-5.391915	3.359596
C	-1.545173	-4.526820	2.363324
C	-1.971356	-3.592477	1.425648
H	-3.034613	-3.518688	1.181893
C	-1.055592	-2.741131	0.798870
C	3.237860	-0.606061	0.804015
C	4.421634	-0.362402	1.507577
H	5.018549	0.520880	1.268593
C	4.859685	-1.229895	2.507270
C	4.148775	-2.400416	2.764148
H	4.528268	-3.097232	3.511430
C	2.270943	-4.052313	2.142631
C	0.765316	-3.835351	2.012892
C	0.318560	-2.842190	1.111885
C	2.494927	-1.757944	1.138315
C	2.978388	-2.700639	2.067148
C	2.637445	-4.827614	3.407307
H	2.161794	-5.817423	3.407745
H	2.332431	-4.287528	4.315605
H	3.720454	-5.003043	3.449054
C	2.720913	-4.874356	0.911458
H	3.809953	-5.028854	0.938805
H	2.471173	-4.346158	-0.020839
H	2.217031	-5.852846	0.904113
C	-3.695260	-2.141582	-1.175495
C	-4.903143	-1.848169	-0.532742
H	-4.914332	-1.139202	0.295722
C	-6.082178	-2.459292	-0.959938
H	-7.023922	-2.227565	-0.459808
C	-6.060370	-3.362213	-2.023335
H	-6.985156	-3.837339	-2.354622
C	-4.858206	-3.654591	-2.665748
H	-4.838849	-4.356150	-3.501073
C	-3.676375	-3.044003	-2.243751
H	-2.740247	-3.268738	-2.761820
C	-0.664129	-1.523351	-2.294771
C	0.076302	-2.699659	-2.454991
H	0.015727	-3.498973	-1.709861
C	0.917818	-2.853987	-3.558216
H	1.498449	-3.770462	-3.674231
C	1.019927	-1.834038	-4.502895
H	1.681472	-1.951477	-5.362669
C	0.281342	-0.660597	-4.346342
H	0.366767	0.140078	-5.082706
C	-0.556878	-0.501031	-3.243583
H	-1.118667	0.424958	-3.112940
C	1.757320	2.238059	0.169474
C	1.011296	3.167235	-0.566262
H	0.961768	3.091321	-1.657169
C	0.301128	4.179767	0.076886
H	-0.285747	4.892633	-0.507898
C	0.317512	4.262425	1.469967
H	-0.257120	5.040339	1.978029
C	1.058626	3.344052	2.211497
H	1.066532	3.401859	3.301499
C	1.779294	2.338845	1.563553
H	2.341844	1.611784	2.154993
C	4.634260	1.550437	-1.067892
C	5.647745	0.845812	-1.732267
H	5.443906	-0.146928	-2.145250
C	6.924842	1.388576	-1.871372
H	7.704451	0.823684	-2.385662
C	7.202623	2.655183	-1.356215
H	8.200027	3.084133	-1.466306
C	6.200870	3.370933	-0.702437
H	6.412784	4.362738	-0.298758
C	4.924714	2.821439	-0.557437
H	4.150058	3.389120	-0.035560
C	-2.826215	1.308919	3.240435
H	-2.607971	0.241481	3.288965
C	-3.144402	2.039080	4.381254
H	-3.179435	1.548594	5.354682
C	-3.423882	3.400730	4.267647
H	-3.685584	3.982564	5.152508
C	-3.378735	4.025261	3.025393
H	-3.620839	5.085606	2.965781
C	-3.060453	5.359577	0.388143
H	-3.145290	5.999941	1.264844
C	-3.008414	5.955367	-0.868829
H	-3.060911	7.042517	-0.946354
C	-2.886650	5.180045	-2.025017
H	-2.846256	5.653686	-3.006161
C	-2.811731	3.799214	-1.904000
H	-2.711307	3.153858	-2.777763
C	-2.713258	1.737508	-0.550637
C	-2.363439	1.107926	0.810376
C	-2.782423	1.935801	1.996954
C	-3.045965	3.309179	1.865910
C	-2.984737	3.967328	0.534924
C	-2.847830	3.200318	-0.640387
H	-2.264463	-5.176341	2.862141
H	5.773559	-1.011510	3.060844
N	1.250922	-1.987041	0.546701
H	0.855795	-1.173524	0.081695

7.11 Transition state $\mathbf{TS1_{NH}}$.

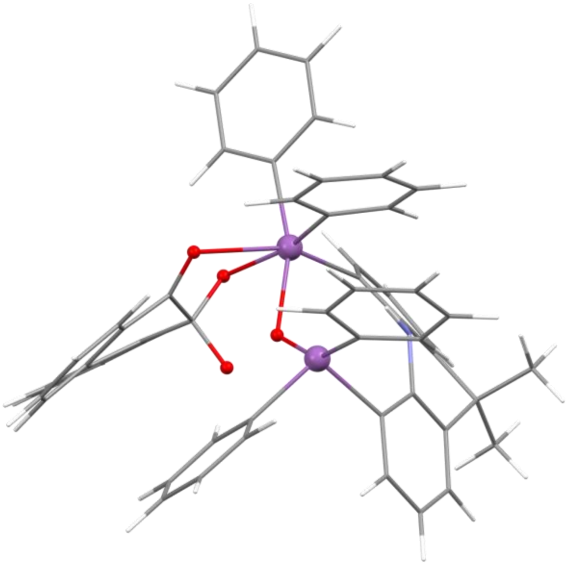

Sb	-0.919192	-1.551259	-0.793852
Sb	1.702818	1.188849	-0.045349
O	-1.872426	0.577285	-1.776737
O	-2.649082	-1.064655	0.060291
O	-0.216328	0.250904	0.316221
O	-1.698270	0.519730	1.360009
C	0.160907	-4.224261	3.144245
H	0.277352	-4.728857	4.103845
C	-0.957435	-4.516187	2.357331
C	-1.160838	-3.842049	1.156859
H	-2.069561	-4.027191	0.577600
C	-0.233180	-2.892099	0.711634
C	2.587546	0.453570	1.760533
C	3.094000	1.278759	2.769100
H	3.223541	2.348593	2.584309
C	3.383283	0.752779	4.027270
C	3.144523	-0.598296	4.298041
H	3.342118	-0.978489	5.300799
C	2.405214	-2.958976	3.485985
C	1.122427	-3.310308	2.719661
C	0.924133	-2.679795	1.476357
C	2.414257	-0.909808	2.040051
C	2.660165	-1.456136	3.311902
C	2.306045	-3.346548	4.960236
H	2.155055	-4.429640	5.065124
H	1.477036	-2.824473	5.459975
H	3.241113	-3.104978	5.483937
C	3.582391	-3.730865	2.847809
H	4.520822	-3.474570	3.361579
H	3.687811	-3.480076	1.783745
H	3.412228	-4.814165	2.936220
C	-2.047307	-2.869812	-2.062570
C	-3.446742	-2.857767	-2.101357
H	-3.993917	-2.147490	-1.481793
C	-4.135017	-3.750541	-2.924803
H	-5.225832	-3.729234	-2.952350
C	-3.435468	-4.667765	-3.707675
H	-3.976465	-5.366349	-4.348178
C	-2.041671	-4.688667	-3.672130
H	-1.487638	-5.402817	-4.283473
C	-1.352856	-3.789625	-2.857800
H	-0.259928	-3.809021	-2.856254
C	0.690149	-1.299200	-2.200678
C	1.789096	-2.159631	-2.104520
H	1.811153	-2.958557	-1.356630
C	2.890349	-1.997084	-2.952168
H	3.753796	-2.656120	-2.848137
C	2.882675	-0.998029	-3.920927
H	3.747165	-0.857186	-4.571327
C	1.760970	-0.176501	-4.057096
H	1.744951	0.597529	-4.826534
C	0.668660	-0.322646	-3.204392
H	-0.191732	0.340118	-3.303782
C	1.112396	3.143331	0.526671
C	-0.053076	3.278168	1.296000
H	-0.634858	2.394065	1.573661
C	-0.476518	4.546920	1.692289
H	-1.381186	4.646647	2.294762
C	0.243686	5.680662	1.313790
H	-0.094963	6.671693	1.621826
C	1.400559	5.548127	0.546351
H	1.970744	6.432321	0.256175
C	1.839773	4.281011	0.157731
H	2.757401	4.184998	-0.427786
C	3.463444	1.578433	-1.251211
C	4.638226	0.853660	-1.018753
H	4.728626	0.230769	-0.123632
C	5.700511	0.922035	-1.922078
H	6.614127	0.356434	-1.730489
C	5.596607	1.714492	-3.065372
H	6.428248	1.769254	-3.769856
C	4.428167	2.439184	-3.306356
H	4.344418	3.060332	-4.200154
C	3.363308	2.360118	-2.409356
H	2.441329	2.911198	-2.623097
C	-4.729964	-0.295406	1.798297
H	-4.350147	-1.316998	1.838415
C	-5.907389	0.071373	2.445668
H	-6.476859	-0.670941	3.006706
C	-6.358648	1.388707	2.366562
H	-7.285859	1.681985	2.860728
C	-5.633505	2.334092	1.646246
H	-6.017126	3.352063	1.573457
C	-3.862769	4.360770	0.327906
H	-4.574396	4.748185	1.056856
C	-3.143233	5.258026	-0.458474
H	-3.307544	6.329427	-0.330888
C	-2.211601	4.806875	-1.396126
H	-1.645194	5.517791	-1.998066
C	-2.001450	3.440788	-1.533329
H	-1.270255	3.044083	-2.240263
C	-2.410489	1.105989	-0.813727
C	-2.677585	0.257794	0.454653
C	-4.007476	0.649555	1.074160
C	-4.441835	1.985952	0.996759
C	-3.665638	2.979037	0.208259
C	-2.709362	2.540976	-0.731364
H	-1.687857	-5.248563	2.702007
H	3.767997	1.402399	4.814172
N	1.903518	-1.777232	1.072321
H	1.769084	-1.375852	0.154954

7.12 Intermediate $\mathbf{I2_{NH}}$

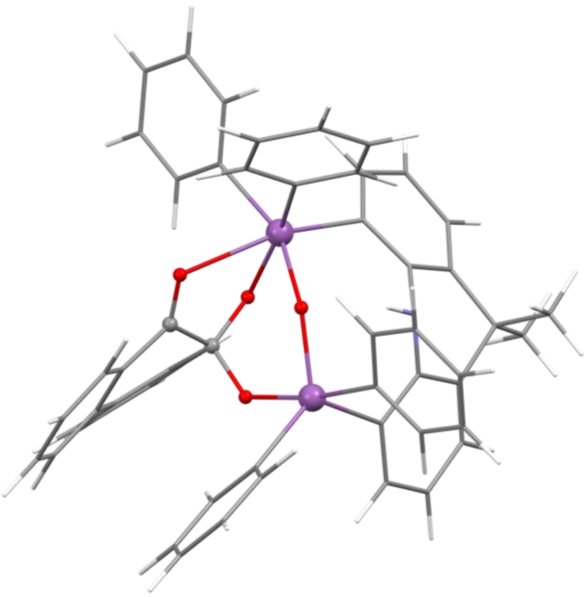

Sb	-1.298064	-1.309402	0.737613
Sb	0.911195	1.634690	-0.008440
O	1.009425	-2.001539	1.314715
O	-0.246851	-1.511722	-0.977128
O	-0.218149	0.383497	0.955891
O	1.477265	0.028716	-1.158018
C	-4.810204	-0.106725	-2.460000
H	-5.483473	0.057410	-3.301994
C	-4.871176	-1.317189	-1.766575
C	-3.964444	-1.582888	-0.741630
H	-3.965597	-2.570423	-0.274583
C	-3.018998	-0.624525	-0.353807
C	-0.109835	2.476525	-1.662304
C	0.517066	3.066152	-2.761687
H	1.598287	3.226182	-2.758733
C	-0.230616	3.410412	-3.886618
C	-1.601623	3.139784	-3.929342
H	-2.158779	3.381490	-4.834983
C	-3.759223	2.251116	-2.772398
C	-3.904533	0.884723	-2.087414
C	-3.057142	0.627348	-0.993909
C	-1.498324	2.269529	-1.696087
C	-2.259731	2.569712	-2.840681
C	-4.399988	2.257494	-4.158936
H	-5.476892	2.052854	-4.085793
H	-3.943221	1.506516	-4.820220
H	-4.295502	3.246968	-4.625078
C	-4.453445	3.322571	-1.901585
H	-4.336216	4.313737	-2.364272
H	-4.021668	3.354877	-0.892846
H	-5.526393	3.095181	-1.816384
C	-1.655638	-3.425941	0.767026
C	-1.093110	-4.283870	-0.184845
H	-0.436261	-3.879532	-0.957000
C	-1.376295	-5.649521	-0.152921
H	-0.929027	-6.311954	-0.895982
C	-2.230995	-6.167801	0.820408
H	-2.452729	-7.236151	0.840982
C	-2.801030	-5.318032	1.766767
H	-3.470822	-5.717249	2.530219
C	-2.510398	-3.952383	1.741846
H	-2.956427	-3.297267	2.495135
C	-1.734138	-0.960959	2.805743
C	-2.706874	-0.012571	3.134581
H	-3.278160	0.488289	2.346451
C	-2.956184	0.307658	4.470863
H	-3.718017	1.048465	4.719654
C	-2.230054	-0.318589	5.483308
H	-2.422385	-0.068913	6.528099
C	-1.259526	-1.268122	5.159634
H	-0.692316	-1.760393	5.951538
C	-1.011763	-1.589946	3.824474
H	-0.249838	-2.333312	3.576682
C	3.000444	1.974501	0.247776
C	3.939660	1.436491	-0.644465
H	3.600059	0.840511	-1.492002
C	5.300335	1.648311	-0.429638
H	6.026784	1.232180	-1.129756
C	5.733727	2.373262	0.681568
H	6.800868	2.527767	0.850771
C	4.803607	2.899204	1.575518
H	5.137461	3.463530	2.447549
C	3.437905	2.709125	1.355522
H	2.715845	3.133309	2.055992
C	0.260538	3.116621	1.435439
C	0.316016	4.469301	1.079181
H	0.671113	4.762787	0.085444
C	-0.091192	5.459315	1.974632
H	-0.048305	6.510232	1.683391
C	-0.559686	5.101208	3.239165
H	-0.881308	5.873074	3.940536
C	-0.623087	3.755874	3.602004
H	-0.996446	3.470503	4.587398
C	-0.216523	2.765884	2.704604
H	-0.284429	1.711801	2.984575
C	1.041863	-2.442274	-3.239999
H	-0.018718	-2.189115	-3.267043
C	1.663633	-3.100597	-4.300675
H	1.087032	-3.375887	-5.184945
C	3.019702	-3.413331	-4.223179
H	3.509908	-3.939602	-5.043391
C	3.751748	-3.069072	-3.089041
H	4.803646	-3.350797	-3.028289
C	5.296394	-1.988694	-0.742391
H	5.879308	-2.170433	-1.645682
C	5.957967	-1.675301	0.444368
H	7.047980	-1.620196	0.449998
C	5.245632	-1.423270	1.617557
H	5.771152	-1.169863	2.538552
C	3.855932	-1.489438	1.595959
H	3.261703	-1.292067	2.490034
C	1.720879	-1.763335	0.352789
C	1.104087	-1.298780	-0.988264
C	1.774123	-2.092395	-2.109582
C	3.144661	-2.398178	-2.020603
C	3.899524	-2.045178	-0.789921
C	3.194767	-1.783691	0.402490
H	0.259326	3.864956	-4.748197
H	-5.597876	-2.075832	-2.058757
N	-2.173392	1.649078	-0.642610
H	-1.665728	1.494357	0.221377

7.13 Transition state **TS2**$_{\mathbf{NH}}$

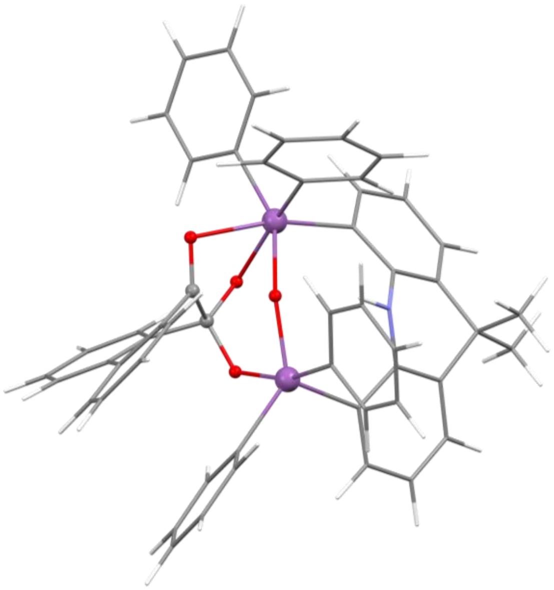

Sb	1.940180	0.930654	0.359571	C	4.107397	4.579316	-0.783644	H	-3.146097	-4.960360	2.836173
Sb	-1.588649	-0.903723	-0.074051	H	3.971323	5.380808	-1.511552	C	-1.882414	-3.678197	4.032125
O	0.422522	2.441068	1.029034	C	5.209142	4.593839	0.072559	H	-1.939466	-4.315208	4.916435
O	0.930226	1.433831	-1.316171	H	5.935807	5.405756	0.012526	C	-1.123459	-2.509330	4.059881
O	-0.083438	0.067498	0.771225	C	5.379770	3.574440	1.008602	H	-0.577695	-2.228930	4.962732
O	-1.213742	0.556020	-1.447985	H	6.237516	3.587035	1.682974	C	-1.045095	-1.693174	2.928271
C	4.314384	-2.399825	-2.410698	C	4.447226	2.538652	1.086309	H	-0.425700	-0.793376	2.945544
H	4.812802	-3.057716	-3.123117	H	4.581070	1.746733	1.829518	C	-0.393905	3.335716	-2.975786
C	4.909975	-1.182868	-2.077112	C	2.168725	0.359273	2.406228	H	0.544661	2.889274	-3.308227
C	4.265043	-0.301708	-1.210407	C	2.649970	-0.921020	2.693918	C	-1.004188	4.372800	-3.683754
H	4.717646	0.671121	-1.008294	H	2.984495	-1.582001	1.888895	H	-0.537356	4.765276	-4.588380
C	3.029348	-0.634504	-0.642432	C	2.703594	-1.373688	4.014645	C	-2.208187	4.906998	-3.229642
C	-0.985927	-2.486290	-1.334317	H	3.073851	-2.377422	4.229234	H	-2.684713	5.724128	-3.773055
C	-1.845962	-3.076029	-2.263171	C	2.282110	-0.544555	5.052280	C	-2.801545	4.412363	-2.068370
H	-2.898059	-2.781352	-2.302650	H	2.322587	-0.896855	6.084479	H	-3.724179	4.866092	-1.703559
C	-1.350656	-4.011754	-3.170173	C	1.812284	0.739754	4.769600	C	-4.134928	3.034365	0.273104
C	0.006785	-4.343245	-3.164423	H	1.487461	1.392996	5.581382	H	-4.824485	3.499751	-0.432266
H	0.380452	-5.049970	-3.905909	C	1.752625	1.192596	3.451258	C	-4.611627	2.593756	1.507198
C	2.378217	-4.104960	-2.121315	H	1.369522	2.190972	3.233276	H	-5.667804	2.722689	1.748789
C	3.099000	-2.779927	-1.844423	C	-3.667307	-0.522076	-0.371669	C	-3.757069	1.988140	2.429456
C	2.487977	-1.894644	-0.936663	C	-4.094935	0.044894	-1.581057	H	-4.136228	1.651034	3.394852
C	0.359033	-2.875632	-1.300622	H	-3.359440	0.316704	-2.340060	C	-2.418410	1.795401	2.096208
C	0.883150	-3.789359	-2.231759	C	-5.451539	0.286399	-1.794216	H	-1.722264	1.304140	2.779617
C	2.907094	-4.786108	-3.381820	H	-5.779873	0.723647	-2.738613	C	-0.558817	1.962960	0.415519
H	3.973950	-5.023846	-3.271447	C	-6.384199	-0.018937	-0.801608	C	-0.414457	1.628902	-1.091830
H	2.777973	-4.150898	-4.270417	H	-7.444804	0.175849	-0.970264	C	-0.990064	2.837975	-1.824230
H	2.384983	-5.737988	-3.550606	C	-5.960150	-0.568229	0.406642	C	-2.207039	3.369717	-1.350883
C	2.596970	-5.050405	-0.918613	H	-6.684809	-0.798559	1.189391	C	-2.792227	2.860292	-0.079563
H	2.062746	-5.997531	-1.085028	C	-4.603806	-0.822831	0.620995	C	-1.957168	2.206314	0.847281
H	2.226503	-4.601230	0.012328	H	-4.279629	-1.244632	1.574874	H	-2.017451	-4.466493	-3.903241
H	3.669952	-5.263299	-0.802220	C	-1.733952	-2.041151	1.760126	H	5.866910	-0.905186	-2.519871
C	3.346391	2.517042	0.223904	C	-2.482774	-3.225141	1.737954	N	1.258991	-2.291431	-0.407532
C	3.176694	3.542701	-0.711162	H	-3.009969	-3.526905	0.826398	H	0.880641	-1.697479	0.322881
H	2.310824	3.526941	-1.376389	C	-2.560960	-4.039764	2.867173				

7.14 Product $\mathbf{P_{NH}}$

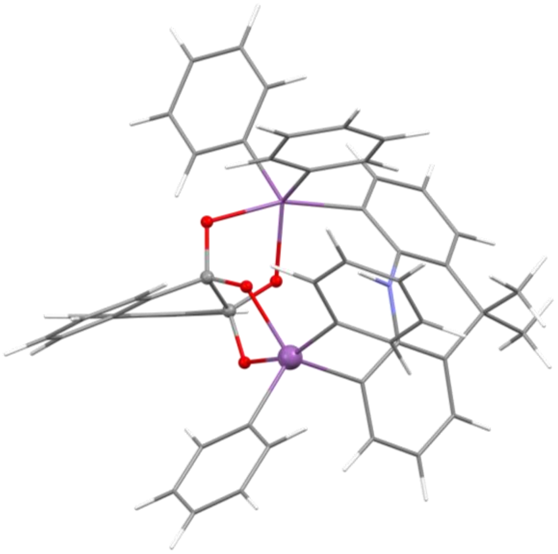

Sb	2.081978	0.317449	0.589603	C	5.027612	3.481277	0.039821	H	-3.067343	-4.883449	2.247249
Sb	-2.077235	-0.341491	-0.186514	H	5.049521	4.448759	-0.463655	C	-2.636191	-3.359770	3.715859
O	0.981397	1.981703	1.028836	C	6.153627	3.020362	0.722277	H	-2.765757	-4.030790	4.566796
O	0.904275	0.439445	-1.025420	H	7.059157	3.628491	0.750973	C	-2.296350	-2.022978	3.924165
O	-0.942469	0.774787	1.031914	C	6.124062	1.787945	1.375029	H	-2.153244	-1.646354	4.938565
O	-1.257888	0.966749	-1.513042	H	7.001349	1.432883	1.917626	C	-2.126103	-1.162778	2.839405
C	3.534880	-3.556997	-2.127874	C	4.967697	1.008883	1.335361	H	-1.839642	-0.124223	3.006910
H	3.842704	-4.341513	-2.819243	H	4.948774	0.043177	1.849023	C	1.304111	2.430647	-2.926586
C	4.479155	-2.631473	-1.687896	C	1.591098	-0.545686	2.471179	H	1.614041	1.408858	-3.149932
C	4.092517	-1.599516	-0.833048	C	1.803484	-1.908889	2.698854	C	1.783537	3.508285	-3.669025
H	4.830912	-0.848914	-0.542807	H	2.207409	-2.546291	1.908005	H	2.472332	3.338164	-4.497777
C	2.770633	-1.486003	-0.395046	C	1.498956	-2.466937	3.942880	C	1.389835	4.804215	-3.335927
C	-1.651349	-1.941246	-1.528300	H	1.651899	-3.533902	4.110716	H	1.775150	5.656850	-3.897314
C	-2.549461	-2.237649	-2.555260	C	1.007251	-1.658627	4.964796	C	0.510231	5.015499	-2.277306
H	-3.476093	-1.665469	-2.653805	H	0.777058	-2.093195	5.939333	H	0.231788	6.035384	-2.008918
C	-2.255810	-3.240442	-3.477974	C	0.802978	-0.294889	4.742716	C	-1.735260	5.288113	-0.304176
C	-1.063636	-3.956774	-3.367355	H	0.416758	0.337283	5.544425	H	-1.676080	6.057441	-1.075128
H	-0.843788	-4.733232	-4.100180	C	1.080140	0.262285	3.496422	C	-2.624015	5.451370	0.755306
C	1.123920	-4.497941	-2.088066	H	0.895226	1.319663	3.302239	H	-3.235773	6.352883	0.814419
C	2.210134	-3.493796	-1.694828	C	-4.016431	0.423844	-0.442241	C	-2.740418	4.459378	1.727837
C	1.843596	-2.458970	-0.813738	C	-4.205969	1.611509	-1.158528	H	-3.439064	4.580165	2.556892
C	-0.464113	-2.680786	-1.416367	H	-3.346229	2.119284	-1.599976	C	-1.970124	3.302399	1.624902
C	-0.160299	-3.703788	-2.335100	C	-5.492515	2.133525	-1.290634	H	-2.060879	2.502643	2.361449
C	1.525816	-5.326881	-3.306311	H	-5.643468	3.063810	-1.840009	C	-0.263252	1.861289	0.451772
H	2.433321	-5.907950	-3.093097	C	-6.581056	1.471265	-0.722361	C	-0.060443	1.459907	-1.043413
H	1.711971	-4.692767	-4.185516	H	-7.585691	1.883203	-0.831318	C	0.425381	2.640335	-1.864926
H	0.737396	-6.050364	-3.554822	C	-6.390162	0.287160	-0.010035	C	0.004620	3.939414	-1.535151
C	0.883383	-5.456697	-0.899110	H	-7.241456	-0.226934	0.438397	C	-0.942463	4.137750	-0.409886
H	0.086037	-6.171657	-1.151233	C	-5.105648	-0.235702	0.137935	C	-1.078170	3.136817	0.567189
H	0.583702	-4.904961	0.002388	H	-4.954004	-1.151159	0.716004	H	-2.951105	-3.458752	-4.288936
H	1.804731	-6.015716	-0.677687	C	-2.295707	-1.633607	1.530068	H	5.511653	-2.698524	-2.032224
C	3.840870	1.469793	0.647085	C	-2.634938	-2.978365	1.330604	N	0.495306	-2.411161	-0.436486
C	3.864283	2.712473	0.005570	H	-2.757143	-3.371662	0.317679	H	0.240387	-1.777319	0.311975
H	2.973768	3.084436	-0.507644	C	-2.805903	-3.837800	2.417720				

Table S2. Energies for all the species involved in the formation of $\mathbf{I1_O}$ and $\mathbf{I1_{NH}}$.

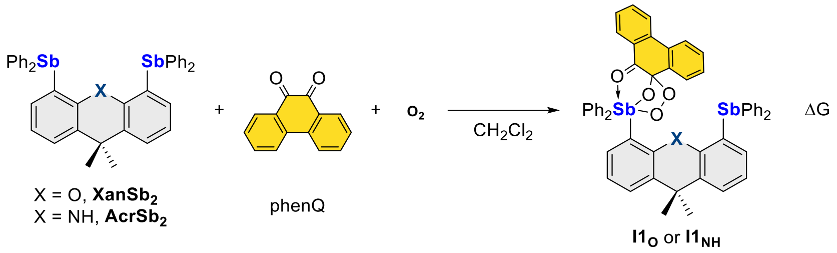

Level of theory	M06-2X/def2-svp for C H N O and aug-cc-PVTZ-PP for Sb		RI-PWPB95-D3(BJ)/def2-tzvpp, SMD(CH_2Cl_2)	
	Single point energy / Hartree	G_{corr} / Hartree	Single point energy / Hartree	G / Hartree
**XanSb₂**	-2059.187519	0.519549	-2060.287691	-2059.768142
**AcrSb₂**	-2039.344123	0.532788	-2040.423125	-2039.890337
**phenQ**	-687.978423	0.144798	-688.557985	-688.413187
**O_2**	-150.082951	-0.0147510	-150.249593	-150.264344
**$I1_O$**	-2897.382822	0.698880	-2899.194994	-2898.496114
**$I1_{NH}$**	-2877.550834	0.711990	-2879.338715	-2878.626725

The free energy change ΔG of forming $\mathbf{I1_O}$ or $\mathbf{I1_{NH}}$ from $\mathbf{XanSb_2}$ or $\mathbf{AcrSb_2}$, phenQ, and O_2 in solution (CH_2Cl_2) are calculated as bellow. The correction term of 1.89 kcal/mol is added to account for the concentration-induced free energy shift associated to transitioning from the gas phase (1 atm) to standard solution phase (1 mol/L).[13]

$\Delta G_{I1O,\ CH2Cl2}$ = [-2898.496114-(-2059.768142)-(-688.413187)-(-150.264344)]×627.51-2×1.89 = -35.43 kcal/mol
$\Delta G_{I1NH,\ CH2Cl2}$ = [-2878.626725-(-2039.890337)-(-688.413187)-(-150.264344)]×627.51-2×1.89 = -40.71 kcal/mol

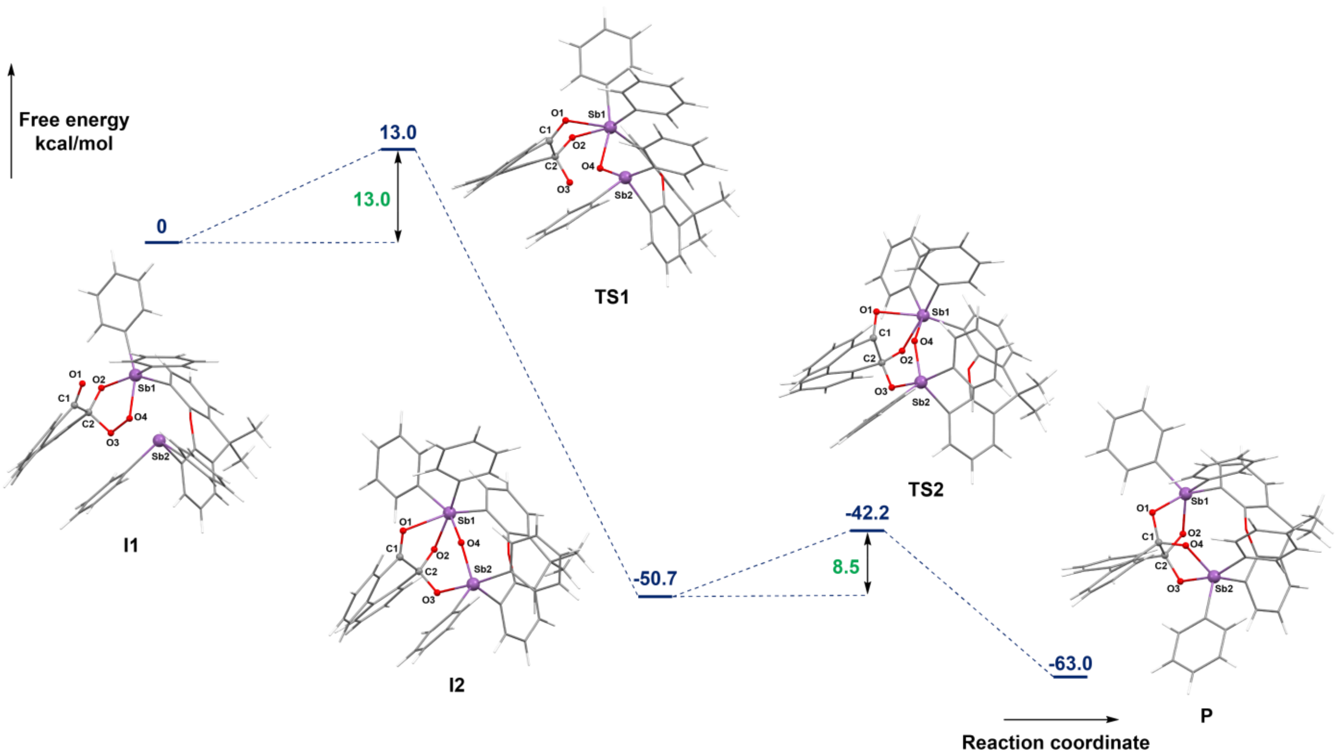

Figure S28 Free energy profile of the proposed reaction mechanism for 4,5-bis(diphenylstibino)-9,9-dimethylxanthene and 9,10-phenanthraquinone.

Table S3. Computed energies of all the involved species for the reaction of compound 4,5-bis(diphenylstibino)-9,9-dimethylxanthene and 9,10-phenanthraquinone.

Level of theory	M06-2X/def2-svp for C H O and aug-cc-PVTZ-PP for Sb		RI-PWPB95-D3(BJ)/def2-tzvpp, SMD(CH_2Cl_2)		
	Single point energy / Hartree	G_{corr} / Hartree	Single point energy / Hartree	G / Hartree	G / kcal·mol^{-1} (relative to **I1$_O$**)
**I1$_O$**	-2897.382822	0.698880	-2899.194994	-2898.496114	0
**TS1$_O$**	-2897.335278	0.697950	-2899.173328	-2898.475378	13.01
**I2$_O$**	-2897.478055	0.701016	-2899.278002	-2898.576986	-50.75
**TS2$_O$**	-2897.459975	0.702705	-2899.266085	-2898.563380	-42.21
**P$_O$**	-2897.498585	0.704091	-2899.300657	-2898.596566	-63.03

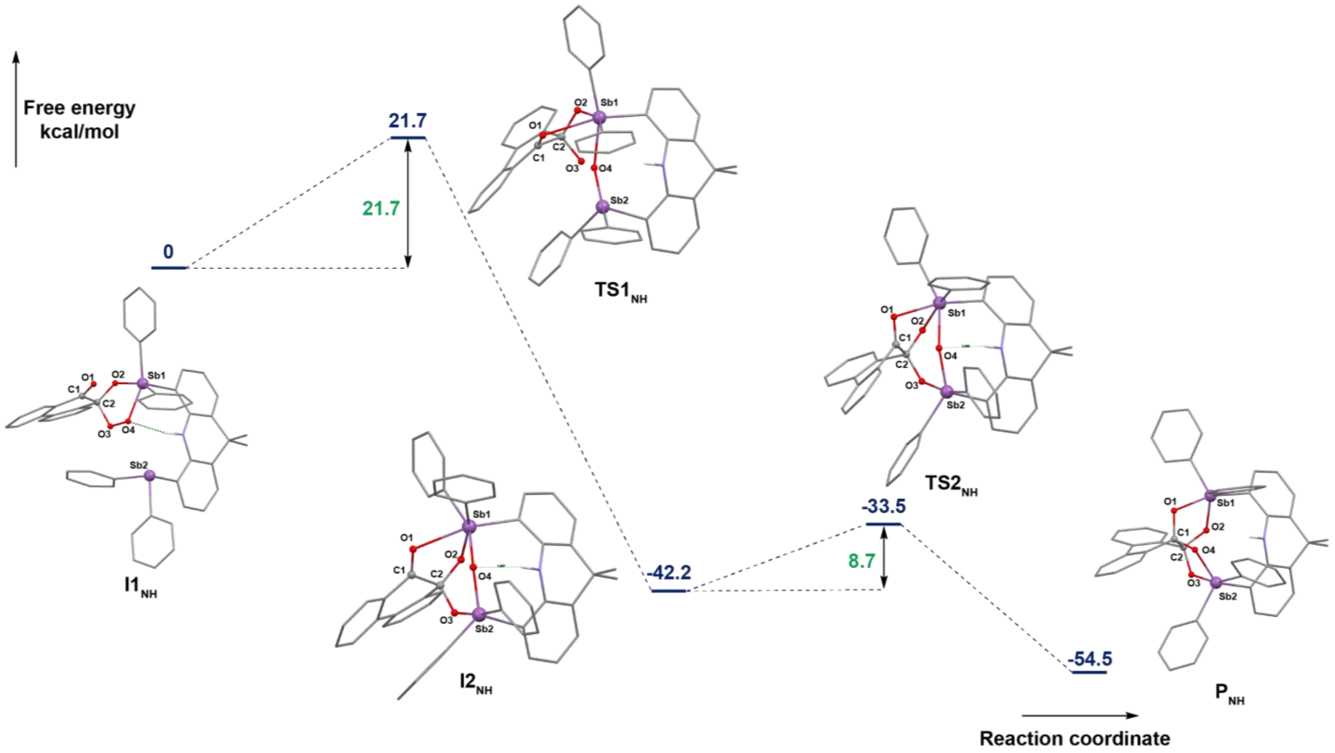

Figure S29 Free energy profile of the proposed reaction mechanism for 4,5-bis(diphenylstibino)-9,9-dimethyl-9,10-dihydroacridine and 9,10-phenanthraquinone.

Table S4. Computed energies of all the involved species for the reaction of compound 4,5-bis(diphenylstibino)-9,9-dimethyl-9,10-dihydroacridine and 9,10-phenanthraquinone.

Level of theory	M06-2X/def2-svp for C H N O and aug-cc-PVTZ-PP for Sb		RI-PWPB95-D3(BJ)/def2-tzvpp, SMD(CH_2Cl_2)		
	Single point energy / Hartree	G_{corr} / Hartree	Single point energy / Hartree	G / Hartree	G / kcal·mol^{-1} (relative to **I1$_{NH}$**)
**I1$_{NH}$**	-2877.550834	0.711990	-2879.338715	-2878.626725	0
**TS1$_{NH}$**	-2877.486949	0.712031	-2879.304234	-2878.592203	21.66
**I2$_{NH}$**	-2877.633525	0.715428	-2879.409379	-2878.693951	-42.19
**TS2$_{NH}$**	-2877.613347	0.717350	-2879.397462	-2878.680113	-33.50
**P$_{NH}$**	-2877.653660	0.717658	-2879.431203	-2878.713545	-54.48

References

1. Nowick, J. S.; Ballester, P.; Ebmeyer, F.; Rebek, J., Jr., Convergent functional groups. 9. Complexation in new molecular clefts. *J. Am. Chem. Soc.* **1990**, *112*, 8902-8906.
2. Pandit, P.; Yamamoto, K.; Nakamura, T.; Nishimura, K.; Kurashige, Y.; Yanai, T.; Nakamura, G.; Masaoka, S.; Furukawa, K.; Yakiyama, Y.; Kawano, M.; Higashibayashi, S., Acid/base-regulated reversible electron transfer disproportionation of N–N linked bicarbazole and biacridine derivatives. *Chem. Sci.* **2015**, *6*, 4160-4173.
3. Hu, J.; Zhang, D.; Harris, F. W., Ruthenium(III) Chloride Catalyzed Oxidation of Pyrene and 2,7-Disubstitued Pyrenes: An Efficient, One-Step Synthesis of Pyrene-4,5-diones and Pyrene-4,5,9,10-tetraones. *J. Org. Chem.* **2005**, *70*, 707-708.
4. Fulmer, G. R.; Miller, A. J. M.; Sherden, N. H.; Gottlieb, H. E.; Nudelman, A.; Stoltz, B. M.; Bercaw, J. E.; Goldberg, K. I., NMR Chemical Shifts of Trace Impurities: Common Laboratory Solvents, Organics, and Gases in Deuterated Solvents Relevant to the Organometallic Chemist. *Organometallics* **2010**, *29*, 2176-2179.
5. Sheldrick, G. M. *SADABS*, *Version 2007/4*, Bruker Analytical X-ray Systems Inc.: Madison, Wisconsin, USA, 2007.
6. Sheldrick, G. M., SHELXT - integrated space-group and crystal-structure determination. *Acta Crystallogr. A* **2015**, *71*, 3-8.
7. Sheldrick, G. M. *SHELXTL*, *Version 6.1*, Bruker Analytical X-ray Systems Inc.: Madison, Wisconsin, USA, 2000.
8. Frisch, M. J.; Trucks, G. W.; Schlegel, H. B.; Scuseria, G. E.; Robb, M. A.; Cheeseman, J. R.; Scalmani, G.; Barone, V.; Petersson, G. A.; Nakatsuji, H.; Li, X.; Caricato, M.; Marenich, A. V.; Bloino, J.; Janesko, B. G.; Gomperts, R.; Mennucci, B.; Hratchian, H. P.; Ortiz, J. V.; Izmaylov, A. F.; Sonnenberg, J. L.; Williams; Ding, F.; Lipparini, F.; Egidi, F.; Goings, J.; Peng, B.; Petrone, A.; Henderson, T.; Ranasinghe, D.; Zakrzewski, V. G.; Gao, J.; Rega, N.; Zheng, G.; Liang, W.; Hada, M.; Ehara, M.; Toyota, K.; Fukuda, R.; Hasegawa, J.; Ishida, M.; Nakajima, T.; Honda, Y.; Kitao, O.; Nakai, H.; Vreven, T.; Throssell, K.; Montgomery Jr., J. A.; Peralta, J. E.; Ogliaro, F.; Bearpark, M. J.; Heyd, J. J.; Brothers, E. N.; Kudin, K. N.; Staroverov, V. N.; Keith, T. A.; Kobayashi, R.; Normand, J.; Raghavachari, K.; Rendell, A. P.; Burant, J. C.; Iyengar, S. S.; Tomasi, J.; Cossi, M.; Millam, J. M.; Klene, M.; Adamo, C.; Cammi, R.; Ochterski, J. W.; Martin, R. L.; Morokuma, K.; Farkas, O.; Foresman, J. B.; Fox, D. J. *Gaussian 16 Rev. C.01*, Wallingford, CT, 2016.
9. Neese, F.; Wennmohs, F.; Becker, U.; Riplinger, C., The ORCA quantum chemistry program package. *J. Chem. Phys.* **2020**, *152*, 224108.
10. Zhao, Y.; Truhlar, D., The M06 suite of density functionals for main group thermochemistry, thermochemical kinetics, noncovalent interactions, excited states, and transition elements: two new functionals and systematic testing of four M06-class functionals and 12 other functionals. *Theor. Chem. Acc.* **2008**, *120*, 215-241.
11. Goerigk, L.; Grimme, S., Efficient and Accurate Double-Hybrid-Meta-GGA Density Functionals—Evaluation with the Extended GMTKN30 Database for General Main Group Thermochemistry, Kinetics, and Noncovalent Interactions. *J. Chem. Theory Comput.* **2011**, *7*, 291-309.
12. Marenich, A. V.; Cramer, C. J.; Truhlar, D. G., Universal Solvation Model Based on Solute Electron Density and on a Continuum Model of the Solvent Defined by the Bulk Dielectric Constant and Atomic Surface Tensions. *J. Phys. Chem. B* **2009**, *113*, 6378-6396.
13. Bursch, M.; Mewes, J.-M.; Hansen, A.; Grimme, S., Best-Practice DFT Protocols for Basic Molecular Computational Chemistry. *Angew. Chem. Int. Ed.* **2022**, *61*, e202205735.
